# Antibody-Based Imaging of Bioreductive Prodrug Release in Hypoxia

**DOI:** 10.1021/jacsau.3c00562

**Published:** 2023-11-01

**Authors:** Çağla Tosun, Antoine L. D. Wallabregue, Maxim Mallerman, Sarah E. Phillips, Claire M. Edwards, Stuart J. Conway, Ester M. Hammond

**Affiliations:** † Department of Oncology, University of Oxford, Old Road Campus Research Building, Oxford OX3 7DQ, U.K.; ‡ Department of Chemistry, Chemistry Research Laboratory, University of Oxford, Mansfield Road, Oxford OX1 3TA, U.K.; § Nuffield Department of Surgical Sciences, University of Oxford, Oxford OX3 7HE, U.K.; ∥ Nuffield Department of Orthopaedics, Rheumatology and Musculoskeletal Sciences, University of Oxford, Oxford OX3 7LD, U.K.; ⊥ Department of Chemistry & Biochemistry, University of California, 607 Charles E. Young Drive East, Los Angeles, California CA90095, United States

**Keywords:** hypoxia, prodrug, imaging, panobinostat

## Abstract

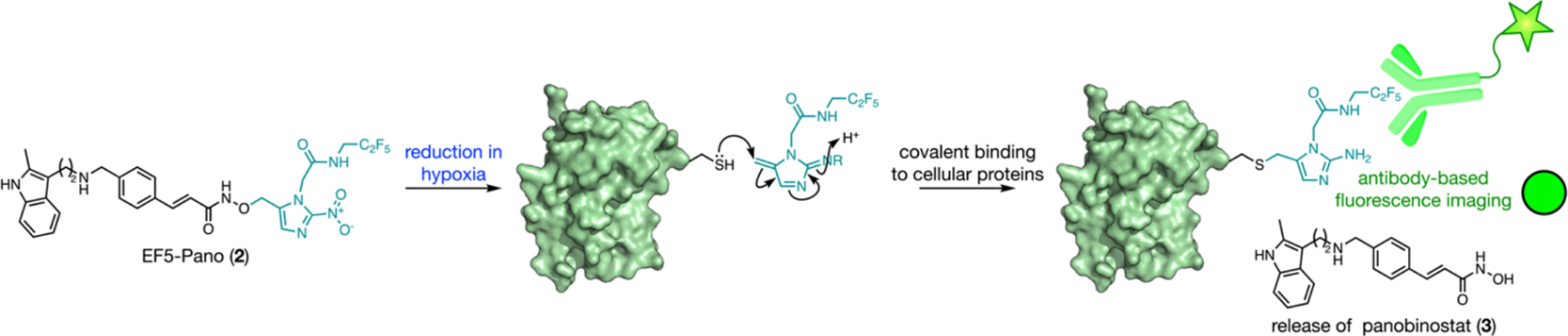

Regions of hypoxia occur in most tumors and are a predictor of poor patient prognosis. Hypoxia-activated prodrugs (HAPs) provide an ideal strategy to target the aggressive, hypoxic, fraction of a tumor, while protecting the normal tissue from toxicity. A key challenge associated with the development of novel HAPs, however, is the ability to visualize the delivery of the prodrug to hypoxic regions and determine where it has been activated. Here, we report a modified version of the commonly used nitroimidazole bioreductive group that incorporates the fluoroethyl epitope of the antibody-based hypoxia imaging agent, EF5. Attachment of this group to the red fluorescent dye, dicyanomethylene (DCM), enabled us to correlate the release of the DCM dye with imaging of the reduced bioreductive group using the EF5 antibody. This study confirmed that the antibody was imaging reduction and fragmentation of the pro-fluorophore. We next employed the modified bioreductive group to synthesize a new prodrug of the KDAC inhibitor Panobinostat, EF5-Pano. Release of EF5-Pano in hypoxic multiple myeloma cells was imaged using the EF5 antibody, and the presence of an imaging signal correlated with apoptosis and a reduction in cell viability. Therefore, EF5-Pano is an imageable HAP with a proven cytotoxic effect in multiple myeloma, which could be utilized in future in vivo experiments.

## Introduction

Hypoxia (insufficient oxygen) is a common feature of the tumor microenvironment, resulting from uncontrolled cell proliferation and aberrant vasculature. Clinically, hypoxia is associated with therapy resistance, metastasis, and poor patient prognosis.^1^ There is, therefore, an unmet need to reverse tumor hypoxia and/or develop strategies to target chemotherapies to hypoxic tumor regions.^2,3^ One such strategy is hypoxia-activated prodrugs (HAPs), which are selectively activated in hypoxia and target drug release to hypoxic regions of tumors while sparing healthy oxygenated tissues.^4^ When present in cells, oxygen inhibits the bioreduction of HAPs that is mediated by endogenous oxidoreductases and prevents the fragmentation and release of the effector compound. When oxygen is sparse, HAPs undergo enzymatically catalyzed bioreduction, which is enhanced by endogenous reductases being upregulated in response to hypoxia in cells.^5,6^

Tirapazamine was the first HAP that showed efficacy in treating a variety of cancers including multiple myeloma.^7^ More recently, the HAP evofosfamide (TH-302) has been reported.^8^ To date, phase I/II clinical trials combining dexamethasone and bortezomib with/without evofosfamide have shown some promising results (limited toxicities, evidence of antitumor activity) in multiple myeloma.^9^ However, phase III trials using evofosfamide failed to improve overall patient survival.^10^ This lack of clinical success has been attributed, at least in part, to the need for hypoxia-based patient stratification to harness the full antitumor potential of HAPs.^11^

**Figure 1:**
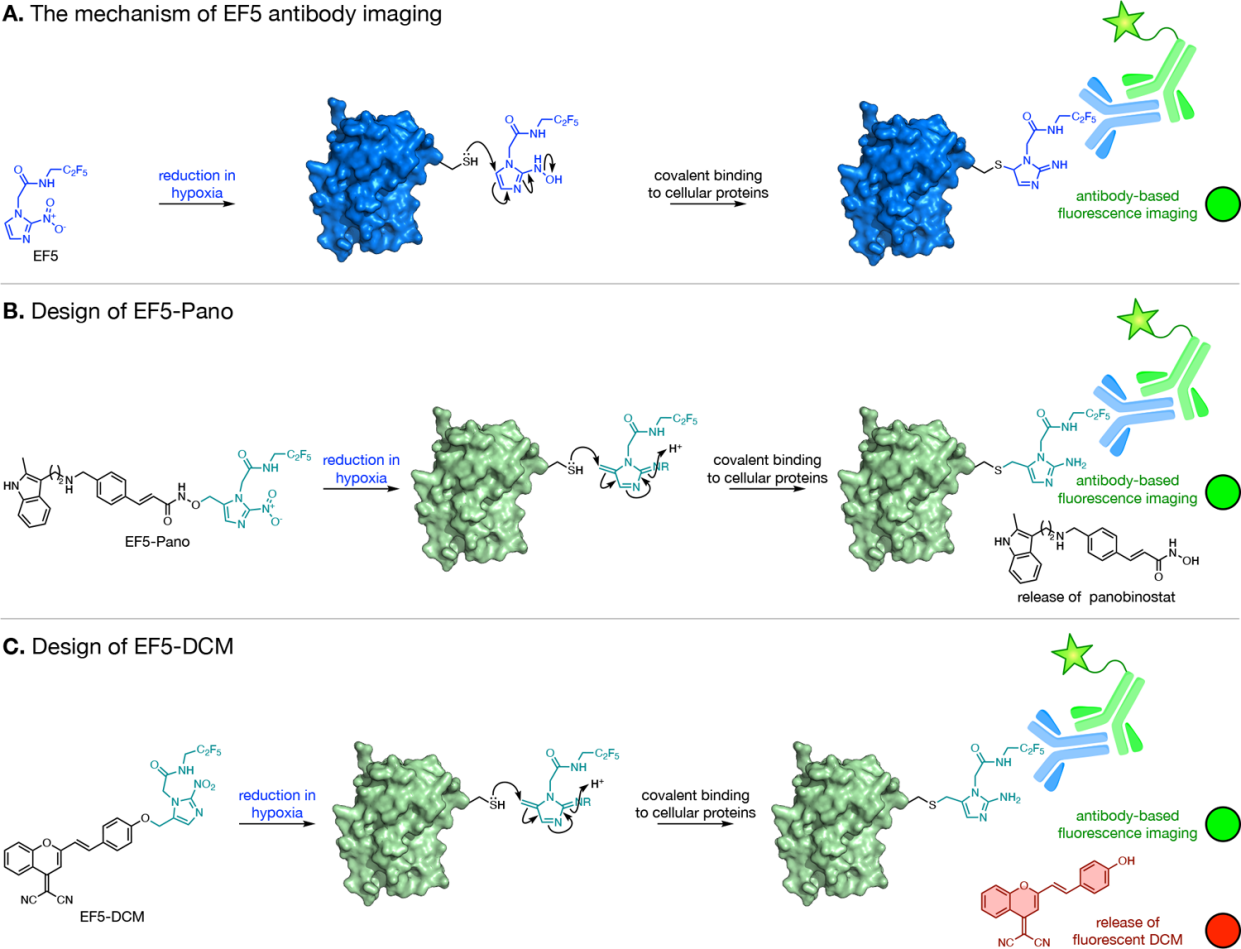
(A) The mechanism for EF5 (**1**) imaging of hypoxia. (B) The proposed design of EF5-Pano (**2**) to enable antibody-based imaging of panobinostat (**3**) release. (C) The design of EF5-DCM (**4**), a pro-fluorophore used to test whether the EF5-derived pro-moiety functioned as intended.

Traditional HAPs, including evofosfamide and tirapazamine, were designed to release a DNA-damaging cytotoxic agent which resulted in overlapping toxicities when combined with other chemotherapies.^6^ As an alternative, a new generation of molecularly targeted HAPs is in development that release a molecular inhibitor of a specific therapeutic target in hypoxia. Examples include the hypoxia-activated Chk1 inhibitor (CH-01), DNA-PK inhibitor (BCCA621C), and HER2 inhibitor (tarloxotinib).^12−15^ We have previously synthesized HAPs of the KDAC inhibitors SAHA and Panobinostat (NI-Pano).^16,17^ NI-Pano demonstrated efficient bioreduction in hypoxia, induced selective cell death in hypoxic esophageal squamous cell carcinoma cells, and led to spheroid growth delay as well as tumor growth delay in a xenograft model.^17^ However, a key challenge to the preclinical development and use of novel HAPs is confirming that the compound reaches the hypoxic areas, enters the cells, and is reduced to release the effector compound. To overcome this problem, we have developed a bioreductive group that incorporates the fluoroethyl epitope of the antibody-based hypoxia imaging agent, EF5 (**1**, Figure 1A).^18^ Attachment of this group to the KDAC inhibitor Panobinostat (**3**) gives a bioreductive prodrug, EF5-Pano (**2**, Figure 1B). The release of this prodrug can be imaged in cells using the EF5 antibody, providing a convenient method to determine whether and where a bioreductive prodrug has been activated.^2^

EF5-Pano (**2**) was validated using multiple myeloma cell lines as the current standard of care for myeloma patients is a combination of KDAC inhibitors including Panobinostat (**3**), bortezomib (proteosome inhibitor), and lenalidomide (immunomodulatory drug).^9,19−22^ However, existing antimyeloma combinations result in gastrointestinal toxicity particularly in elderly patients. As myeloma cells reside in a hypoxic tumor microenvironment, it is likely that the use of EF5-Pano (**2**) would reduce the observed toxic side effects.^23^

## Results and Discussion

To develop a prodrug that enables imaging of its activation, we sought to combine the reactivity of the nitroimidazole group employed in NI-Pano, with the epitope recognized by the EF5 antibody. EF5 (**1**) is a nitroimidazole-based agent that is reduced in hypoxia to give a hydroxylamine derivative (Figure 1A).^2^ This derivative is electrophilic and is thought to conjugate to biological macromolecules via the mechanism shown in Figure 1A. An antibody has been raised against this compound, which binds to the fluoroethyl group-containing epitope of reduced EF5, allowing imaging of regions of hypoxia in vitro and ex vivo.^2^

As our previously developed bioreductive prodrug of panobinostat employed a nitroimidazole group, we reasoned that it should be possible to incorporate the fluoroethyl group into the 1-methyl-nitroimidazole group. When the nitroimidazole group undergoes bioreduction, it is thought to produce the electrophilic quinone-like structure shown in Figure 1B. While we have shown that this group is not toxic, its fate in cells was unknown.^17^ We proposed that this electrophilic group could react covalently with biomacromolecules in a manner similar to EF5 (**1**). If true, that should allow us to image this group using the EF5 antibody, provided that the epitope is sufficiently similar. As conjugation to the biomacromolecules requires elimination of the cargo molecule (e.g., panobinostat), the resulting antibody-based imaging would provide a snapshot of where the cargo is released.

Before synthesizing EF5-Pano (**2**), we wished to determine whether it was possible to image the release of a cargo molecule using the EF5-derived prodrug. We wanted to ensure that antibody imaging correlated with the reduction *and* fragmentation of the prodrug and that we were not imaging the unreduced prodrug or the reduced but unfragmented prodrug. To achieve this, we designed a pro-fluorophore of the fluorescent dye DCM (**5**) conjugated to the EF5-derivative protecting group, EF5-DCM (**4**, Figure 1C). This dye was selected as we have previously used a nitroimidazole-based pro-fluorophore of this dye to image hypoxia.^17^ As the dye is only fluorescent when the phenol group is unsubstituted, this approach enables us to correlate reduction of the pro-fluorophore *and* release of the dye with the appearance of fluorescence using antibody-based imaging.

**Scheme 1:**
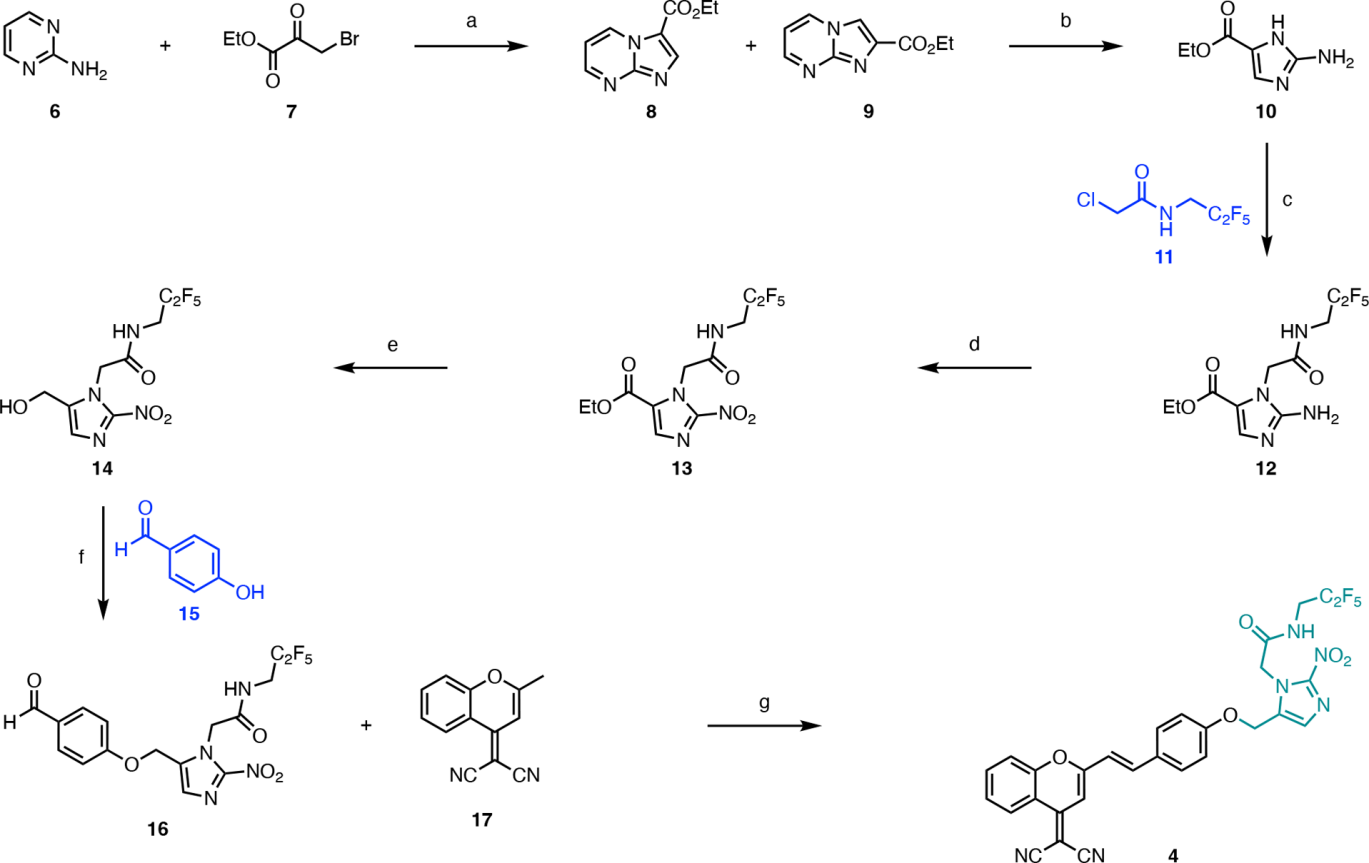
Synthesis of 2-(5-(Hydroxymethyl)-2-nitro-1*H*-imidazol-1-yl)-*N*-(2,2,3,3,3-pentafluoropropyl)acetamide (**14**) and EF5-DCM (**4**) *Reagents and conditions:* (a) EtOH, 75 °C, 85–97%; (b) H_2_NNH_2_·H_2_O, EtOH, 75 °C,72–96%; (c) Cs_2_CO_3_, DMF, 50 °C, (50–55%); (d) NaNO_2 (aq)_, AcOH, 0 °C → RT, (60–85%); (e) NaBH_4_, THF, EtOH, 0 °C, (45–67%); (f) DIAD, PPh_3_, THF, RT, 2.5 h, (50–64%); (g) EtOH, piperidine, reflux, (30–42%).

EF5-DCM (**4**) was synthesized as shown in Scheme 1. Condensation of 2-aminopyrimidine (**6**) with ethyl 3-bromo-2-oxopropanoate (**7**) gave isomeric imidazopyrimidines **8** and **9**, in a combined yield of 85–97%. Treatment of a mixture of **8** and **9** with hydrazine hydrate gave ethyl 2-amino-1*H*-imidazole-4-carboxylate (**10**) in 72–96% yield. Reaction of chloro-*N*-(pentafluoropropyl) acetamide (**11**) with imidazole **10** gave derivatized aminoimidazole **12**. Installation of the nitro group using diazotization gave **13**, which was reduced to give the alcohol **14**. This compound was employed in a Mitsunobu reaction to alkylate 4-hydroxybenzaldehyde **15**. Knovenagal condensation of aldehyde **16** with **17** gave EF5-DCM (**4**).

To ensure that **4** was able to undergo bioreduction and fragmentation as expected, we first assessed its reactivity using an in vitro enzyme assay. We have previously shown that compounds that are bioreduced in this assay also undergo bioreduction in a cellular setting.^12,15−17,24−26^ Treatment with NADPH-cytochrome P450 reductase (CYP004, expressed in *Escherichia coli*, purchased from Cypex) in hypoxia resulted in the bioreduction of EF5-DCM (**4**) to yield DCM (**5**) as judged by fluorescent spectroscopy (Figure S1) and HPLC analysis (Figure S2). A fluorescence recovery of 61% was observed after 30 h, and a peak consistent with this level of DCM release was detected using HPLC analysis. No increase in fluorescence was seen in normoxia after 30 h.

To determine whether the proposed electrophilic product of bioreduction could be trapped by nucleophiles, we investigated the alkylation of l-glutathione and an L94C-containing mutant of bromodomain-containing protein 4 [BRD4(1)^L94C^].^27^ Treatment of **4** with NADPH-cytochrome P450 reductase (CYP004) for 25 h in the presence of l-glutathione in hypoxia resulted in the bioreduction of EF5-DCM (**4**) to yield DCM (**5**), as judged by fluorescence spectroscopy (Figure S3). Using high-resolution mass spectrometry (Figure S3E), we observed a peak corresponding to the [M – H]^−^ of the alkylated of l-glutathione adduct. Under these conditions, no increase in fluorescence or alkylation of l-glutathione was seen in normoxia after 25 h. We only observed the mass corresponding to the fully reduced aminoimidazole, with no intermediate products observed under these conditions.

We next investigated whether the electrophilic bioreduction product could alkylate a cysteine-containing protein. While a number of proteins were not stable under the assay conditions, we identified BRD4(1)^L94C^ as being suitable for this experiment. We have recently reported that this protein is akylated by a range of acetyl-lysing-mimicking fragments, making it ideal for this study.^27^ Treatment of **4** with NADPH-cytochrome P450 reductase (CYP004) for 5 h in the presence of BRD4(1)^L94C^ in hypoxia resulted in alkylation of BRD4(1)^L94C^ to generate two protein adducts, as observed in the deconvoluted mass spectrum (Figure S4C). We observed peaks consistent with both the amino- and hydroxylaminoimidazole-labeled BRD4(1)^L94C^, with a total of 84% alkylation detected (32% of the amino-imidazole adduct and 52% of the hydroxylamine adduct). When the experiment was repeated in normoxia, these protein adducts were not observed.

**Figure 2:**
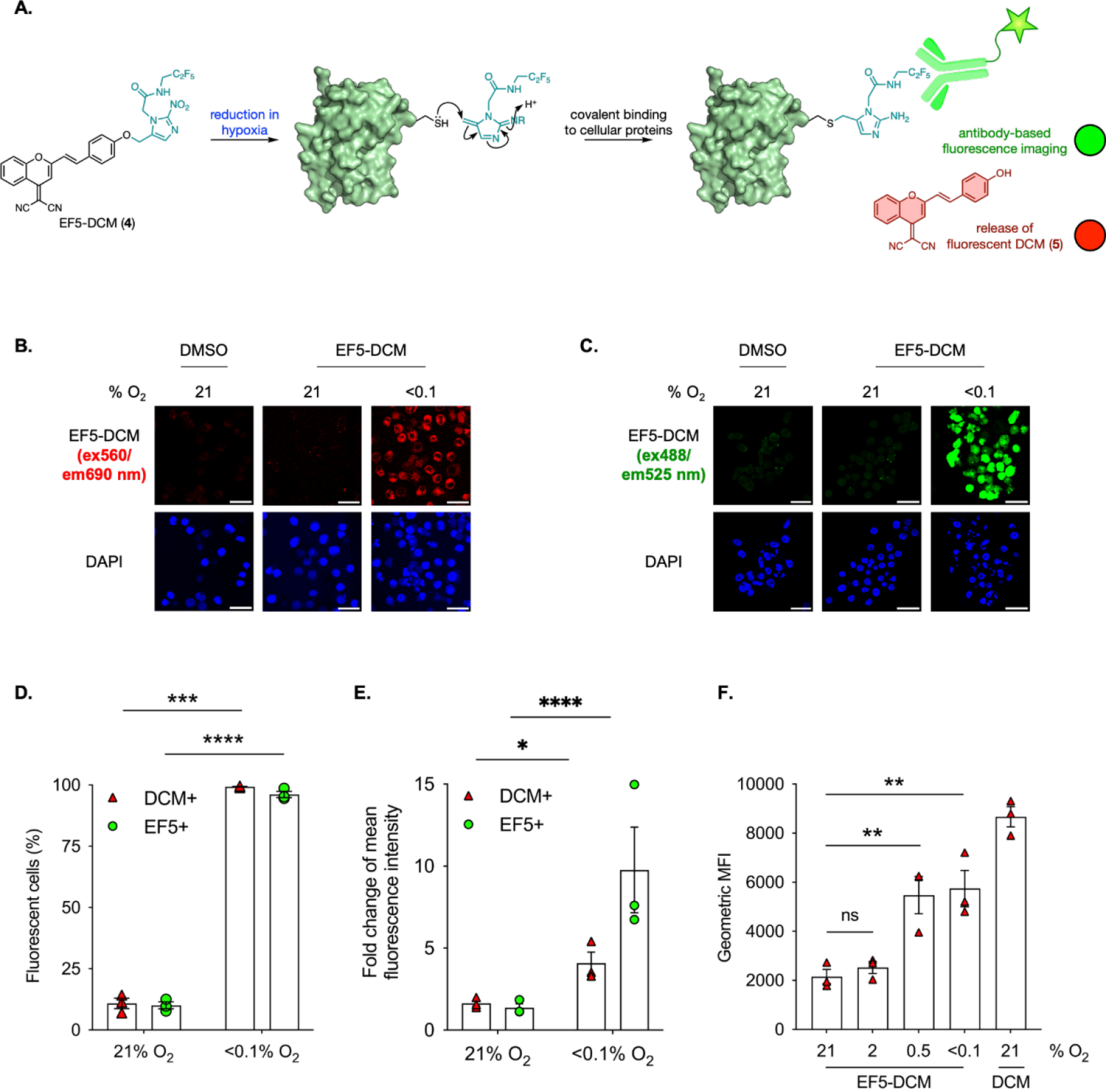
EF5-DCM (**4**) releases fluorescent DCM (**5**) and an antibody-detectable EF5 derivative in hypoxia but not normoxia. (A) The proposed mechanism by which EF5-DCM (**4**) functions. (B) MM.1S cells were exposed to normoxia (21% O_2_) or hypoxia (<0.1% O_2_) for 24 h in the presence of EF5-DCM (**4**, 10 μM) or (C) stained for EF5. Cells were fixed and stained for DAPI. Scale bar: 20 μm. (D) Graph showing the percentage of cells positive for DCM and EF5. Significance: Student’s/Welch’s *t* test. ***: *p* < 0.0005, ****: *p* < 0.00005. (E) Graph showing the fold change in fluorescence intensity in DCM and EF5 signal from B and C, respectively. Significance: Student’s *t* test. ****: *p* < 0.00005, *: *p* < 0.05. (F) MM.1S cells were treated with EF5-DCM (**4**, 10 μM) or DCM (**5**, 2 μM) and exposed to the oxygen concentration shown for 24 h. Cells were processed using flow cytometry, and the geometric mean of the measured fluorescent intensity is shown (APC-A700-A, ex638/em712/25 nm). Data presented are geometric mean fluorescence intensities of DCM (**5**). Significance one-way ANOVA. **: *p* < 0.005, ns: nonsignificant. (A–F) Data from three independent experiments (*n* = 3), mean ± s.e.m are displayed unless otherwise indicated.

Based on these data, we hypothesized that EF5-DCM (**4**) would be reduced in hypoxic conditions to release both DCM (**5**) and an electrophilic EF5 derivative in cells (Figure 2A). We further predicted that the compound would *not* be antibody-detectable in the form of EF5-DCM (**4**). To investigate this, we used radiobiological levels of hypoxia (<0.1% O_2_) as we predicted these levels would be most likely to switch on fluorescence. MM.1S myeloma cells were exposed to EF5-DCM (**4**, 10 μM) in either normoxia (21% O_2_) or hypoxia (<0.1% O_2_). As DCM (**5**) does not bind covalently to cellular macromolecules, any change in fluorescence was visualized by using microscopy without staining for EF5. In parallel, cells treated with EF5-DCM (**4**) were stained for EF5. As expected, in normoxic conditions, we saw no fluorescent resulting from DCM (**5**) release or any antibody-detectable EF5 staining. In contrast, after exposure to hypoxia almost 100% of the cells were positive for red fluorescence resulting from DCM (**5**) release, and also for green fluorescence resulting from the bound EF5 antibody (Figure 2). This result is consistent with the EF5-derivative pro-moiety functioning as outlined in Figure 1A.^28^

Having determined that EF5-DCM (**4**) functioned as expected in radiobiological hypoxia (<0.1% O_2_), we wanted to investigate the oxygen dependency of its activity, as the tumor microenvironment is heterogeneous and often contains gradients of hypoxia.^28^ The generation of fluorescent DCM (**5**) in response to hypoxia enabled the use of flow cytometry as an efficient means to further test EF5-DCM. MM.1S cells were exposed to oxygen levels ranging from 2 to <0.1% O_2_ in the presence of EF5-DCM (**4**). A significant increase in DCM (**5**) was observed at oxygen concentrations below 0.5% (Figure 2F). As the aqueous (dissolved) concentration of oxygen could be significantly lower in the cells exposed to 0.5% O_2_ (gas phase), measurements were made to determine the oxygen concentration in the media and were found to be approximately 0.5% O_2_ for the duration of the experiment (Figure S5).

**Scheme 2:**
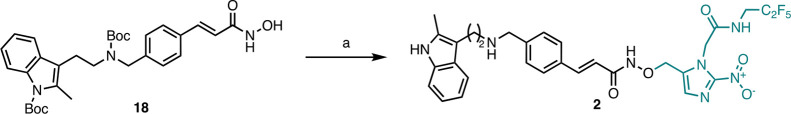
Synthesis of EF5-Pano (**2**) *Reagents and conditions:* (a) i. DIAD, PPh_3_, THF, rt, 17%; ii. TFA, TIPS, CH_2_Cl_2_, rt, 46%.

Importantly, we found that the oxygen dependence with which the EF5 derivative became imageable after treatment with EF5-DCM (**4**) and EF5 (**1**) differed. Specifically, EF5 (**1**) was detectable in cells treated at 1% O_2_ and below, while a positive signal was not observed from EF5-DCM (**4**) at 1% O_2_ (Figure S6). Having demonstrated that EF5-DCM (**4**) reduction and fragmentation could be visualized using the EF5 antibody, we next applied this approach to the imaging of hypoxia-dependent panobinostat release. To achieve this, we synthesized a HAP of panobinostat (**3**), using our EF5-derived pro-moiety, EF5-Pano (**2**). The synthesis of EF5-Pano (**2**) was based on our previously reported synthesis of NI-Pano (see Supporting Information for details), which gave the di-Boc-protected hydroxamic acid **18** (Scheme 2). The EF5-based pro-moiety was added using alcohol **14** under Mitsunobu conditions. Final deprotection, using trifluoroacetic acid (TFA) and triisopropylsilane (TIPS), afforded EF5-Pano (**2**) (Scheme 2).

**Figure 3:**
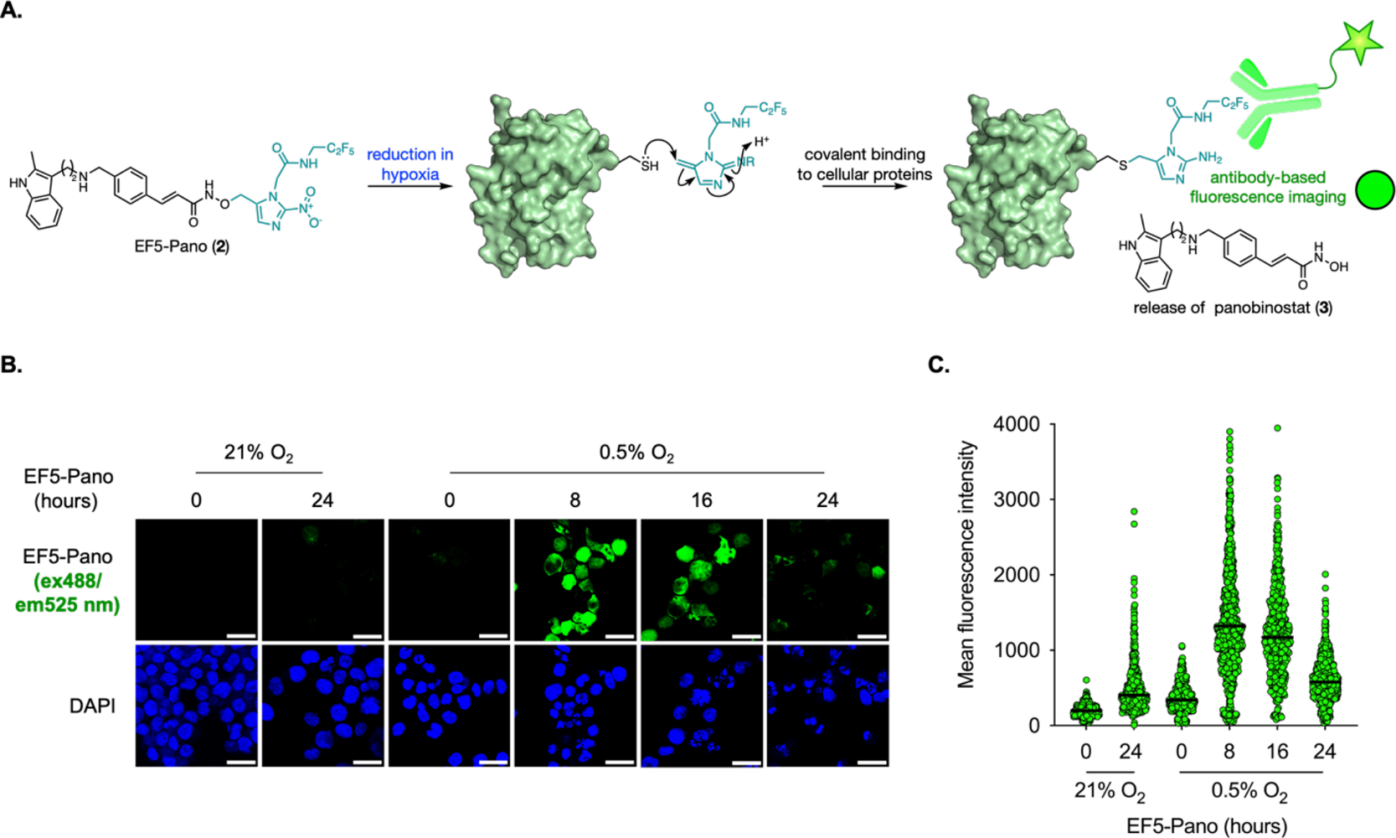
EF5-Pano (**2**) bioreduction is detected by using the EF5 antibody in 0.5% O_2_. (A) The mechanism of EF5-Pano (**2**) bioreduction in hypoxia. (B) MM.1S cells were exposed to 21% O_2_ or 0.5% O_2_ for the indicated time periods in the presence of EF5-Pano (**2**, 20 μM). Cells were fixed and stained for DAPI and EF5. Scale bar represents 20 μm (*n* = 3). (C) Data were from a representative experiment where each dot represents a cell (minimum of 200 cells per treatment). Black lines represent the average fluorescence.

MM.1S cells were treated with EF5-Pano (**2**) and exposed to 21% O_2_ or 0.5% O_2_ followed by staining for EF5. Remarkably, the EF5 antibody was able to detect the reduced product of the prodrug in the majority of cells treated with EF5-Pano (**2**) in hypoxia (0.5% O_2_), but the antibody did not bind in the cells treated in normoxia (21% O_2_) (Figure 3). These data demonstrate that EF5-Pano (**2**) is reduced as soon as 8 h in 0.5% O_2_ to release a derivative that can be detected by the EF5 antibody. In parallel, we used EF5 alone as a control and observed that the EF5 is also detectable after 8 h in hypoxia (0.5% O_2_) (Figure S7).

**Figure 4:**
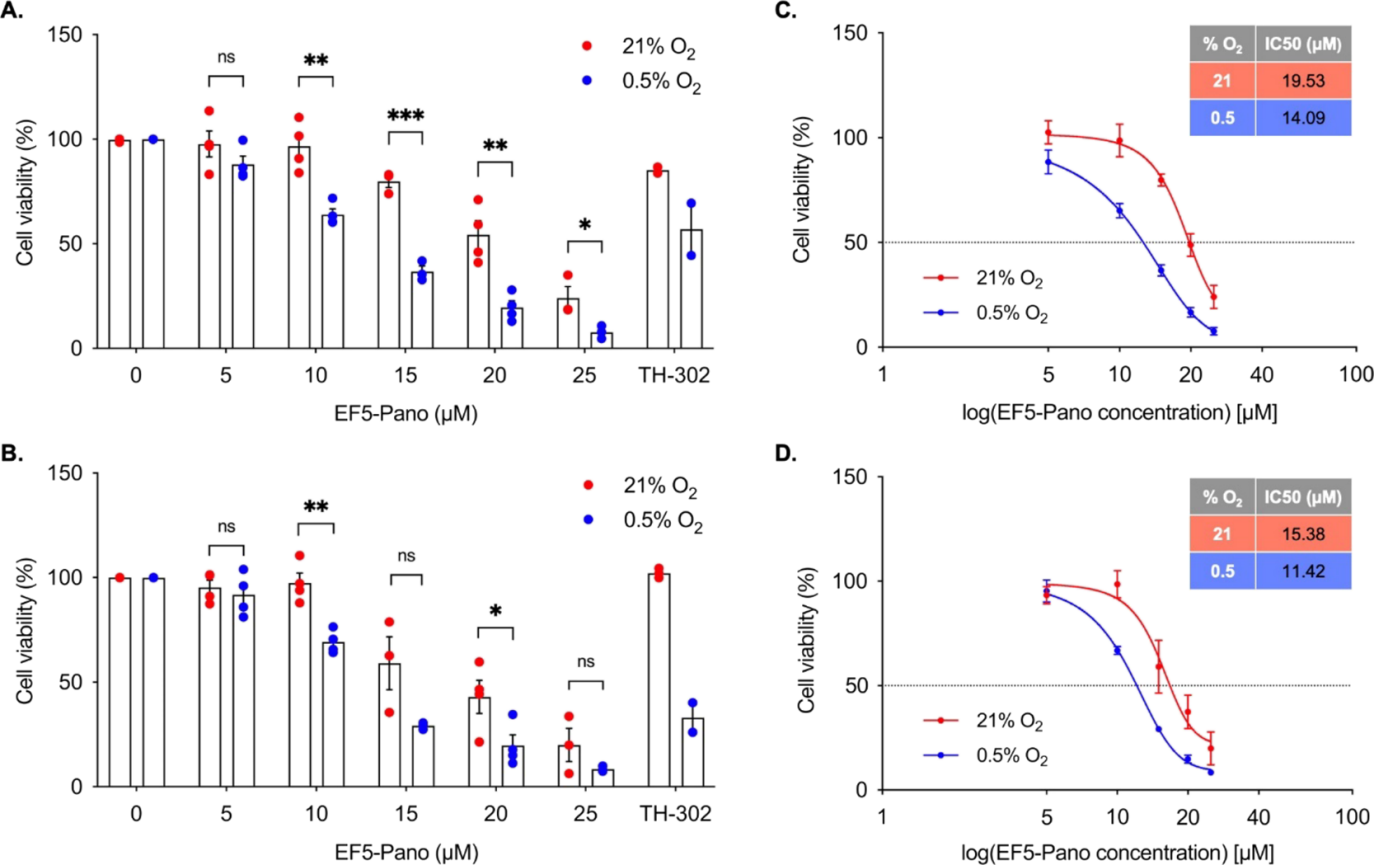
EF5-Pano (**2**) selectively kills multiple myeloma cells at 0.5% O_2_. (A) MM.1S and (B) JJN3 cells were exposed to the concentrations of EF5-Pano (**2**, 0–25 μM) shown for 24 h at either 21% O_2_ or 0.5% O_2_. Cell viability was then assessed using an MTT assay. TH-302 (10 μM, 24 h) was used as a positive control. Data are expressed as percentage viability relative to vehicle control for each oxygen concentration. Data are mean ± s.e.m. (*n* = 3/4). Significance: Two-way ANOVA. *: *p* < 0.05. **: *p* < 0.005. ***: *p* < 0.0005. ns, nonsignificant. Cell viability was also plotted against a logarithmic scale and fitted to a curve to calculate IC_50_ values for (C) MM.1S and (D) JJN3 cells. Data are mean ± s.e.m.

It is well-established that panobinostat leads to a loss of viability in a variety of cancer types including MM.^17,29^ To confirm this, we exposed MM.1S and JJN3 cells to panobinostat and carried out an MTT assay where a dose-dependent decrease in viability was observed in both cell lines with IC_50_ values broadly similar to those previously reported (Figure S8).^30,31^ We next investigated the impact of EF5-Pano (**2**) on the viability of both MM.1S and JJN3 (multiple myeloma) cells, determined by using the MTT assay. In both MM.1S and JJN3 cells, EF5-Pano (**2**) had a greater effect to reduce cell viability in 0.5% O_2_ compared to 21% O_2_. TH-302 was included as a positive control and as expected, reduced cell viability in 0.5% O_2_ compared to 21% O_2_ (Figure 4A,B). IC_50_ values were calculated and found to differ significantly between normoxia and hypoxia in both cell lines (Figure 4C,D. MM.1S: Norm 19.53, Hyp 14.09 μM (*p* = 0.024). JJN3: Norm 15.38, Hyp 11.42 μM (*p* = 0.024).

We next investigated whether treatment with EF5-Pano (**2**) impacted cell viability and specifically through causing apoptotic cell death which has been implicated as the mechanism of death after panobinostat treatment.^21^ MM.1S and JJN3 cells were treated with EF5-Pano (**2**, 10, or 20 μM) in 21% O_2_ or 0.5% O_2_ and stained for EF5. The levels of apoptosis were determined by observing the changes to nuclear morphology (an example of apoptosis cell morphology is shown Figure S9).^32^

**Figure 5:**
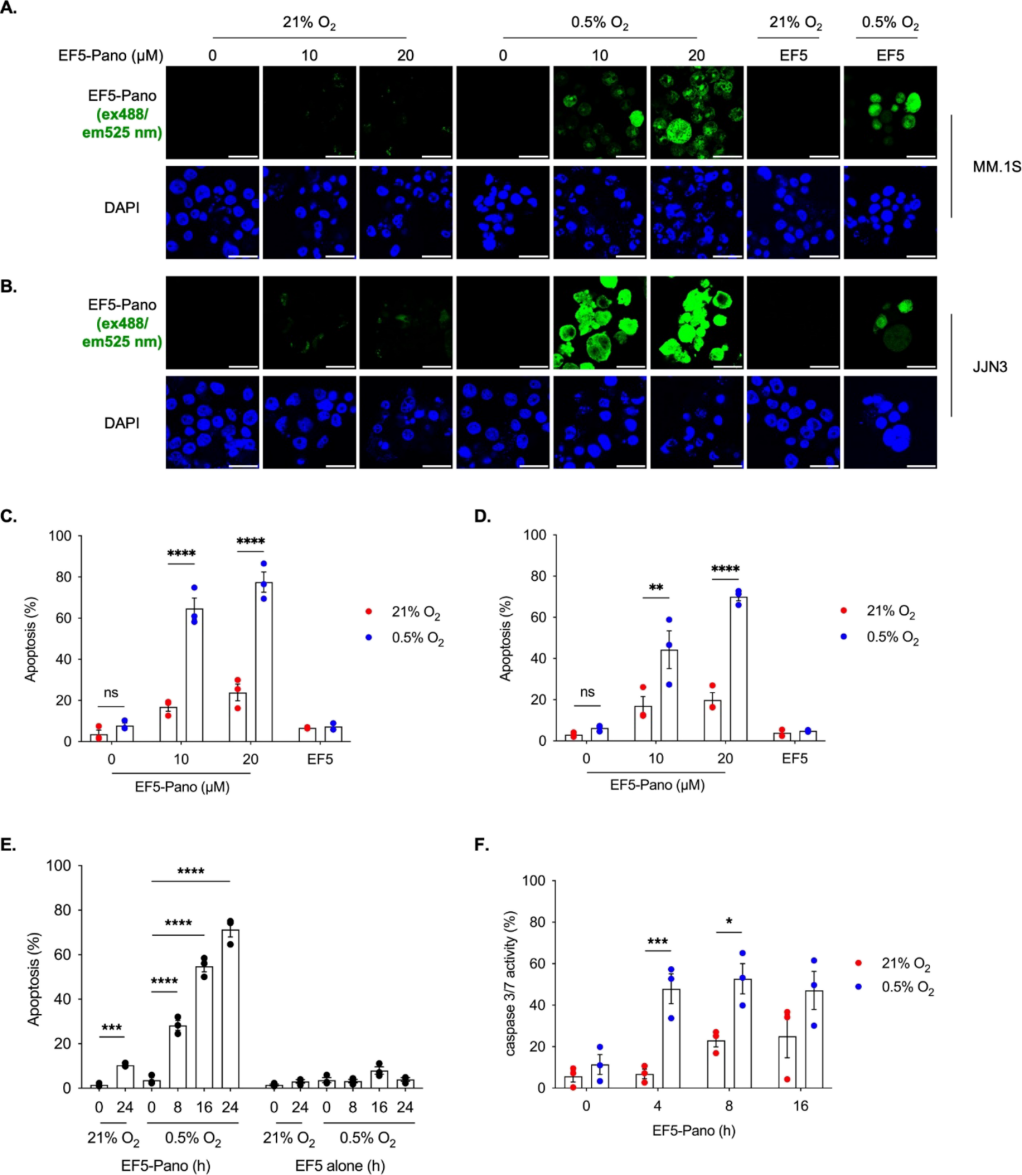
EF5-Pano (**2**) induces apoptosis selectively in hypoxia (0.5% O_2_). (A) MM.1S and (B) JJN3 cells were exposed to 21% O_2_ or 0.5% O_2_ for 24 h in the presence of EF5-Pano (**2**, 0, 10, 20 μM) or EF5 (**1**) alone (20 μM). Cells were fixed and stained for DAPI and EF5. Scale bar represents 20 μM. (C, D) Plots showing the quantification of apoptosis in response to EF5-Pano (**2**) and EF5 (**1**) alone in MM.1S and JJN3 cells, respectively. (E) Plot showing quantification of apoptosis in response to EF5-Pano (20 μM) or EF5 alone (40 μM) in MM.1S cells exposed to 21% O_2_ or 0.5% O_2_ for the indicated time points. (F) Plot showing percentage of caspase 3 activity in response to EF5-Pano (20 μM) in MM.1S cells exposed to 21% O_2_ or 0.5% O_2_ for the indicated time points. (A–F) Data from three independent experiments (*n* = 3), mean ± s.e.m are displayed unless otherwise indicated. Significance: Two-way ANOVA or student’s *t* test. * *p* < 0.05, ** *p* < 0.005, *** *p* < 0.0005, **** *p* < 0.00005.

In both MM.1S and JJN3 cells, treatment with EF5-Pano (**2**) led to significantly more apoptosis in 0.5% O_2_ compared to 21% O_2_ (Figure 5A–D). Importantly, treatment with EF5 (**1**) alone showed no increase in apoptosis in either 21% O_2_ or 0.5% O_2_, and exposure of the cells to 0.5% O_2_ in the absence of EF5-Pano (**2**) did not induce apoptosis. Finally, we explored the kinetics of the induction of apoptosis in response to EF5-Pano and further confirmed the mechanism of death as apoptosis. MM.1S cells treated with EF5-Pano and exposed to hypoxia (0.5% O_2_) underwent a significant increase in apoptosis after 8 h (Figure 5E). Furthermore, an increase in the activity of caspase 3/7 was detected after 4 h treatment in hypoxia confirming both the mechanism of death and also demonstrating that prolonged exposure times are not required to activate EF5-Pano (Figure 5F).

## Conclusions

In conclusion, we have developed a modified version of the commonly used nitroimidazole bioreductive group that incorporates the fluoroethyl epitope of the antibody-based hypoxia imaging agent EF5 (**1**). Attachment of this group to the red fluorescent dye, DCM (**5**), enabled us to correlate the release of the DCM dye (**5**) with imaging of the reduced bioreductive group using the EF5 antibody. This study confirmed that the antibody was imaging reduction and fragmentation of the pro-fluorophore. In addition, this result strongly suggests that the product of fluoroethyl nitroimidazole bioreduction forms covalent bonds with intracellular biomolecules, which fixes its location and imaging signal to regions that have experienced hypoxia. This is interesting as the product of EF5-DCM (**4**)/EF5-Pano (**2**) bioreduction is different to the product of EF5 (**1**) bioreduction, and so covalent reaction with cellular nucleophiles was not a given. We also note that we have previously shown that the bioreductive product of nitroimidazole-based prodrugs is not toxic, despite it apparently acting as an intracellular electrophile.^17^ We next employed the modified bioreductive group to synthesize a new prodrug of the KDAC inhibitor Panobinostat, EF5-Pano (**2**). Activation of EF5-Pano (**2**) in hypoxic multiple myeloma cells was imaged using the EF5 antibody, and the presence of an imaging signal correlated with apoptosis and a reduction in cell viability. The scientific rationale for using a HAP to treat patients with multiple myeloma is supported by the fact that both evofosfamide and tirapazamine have been tested clinically in this context.^24,33^ The lack of success with these agents can be partially attributed to the lack of patient stratification for hypoxia. By building in the ability to image prodrug delivery and activation, as shown here with EF5-Pano, it becomes possible to fully characterize the mechanism of action of EF5-Pano (**2**), and HAPs more widely, preclinically.

## Methods

### Cell Lines and Reagents

MM.1S (ATCC CRL-2974) and JJN3 (DSMZ, ACC 541) human multiple myeloma cells were a kind gift from Prof. Udo Oppermann, University of Oxford. Short tandem repeat DNA (STR) profiling was used for cell line authentication. Cells were grown in RPMI medium supplemented with 10% fetal bovine serum (FBS). Cells were cultured in a humidified incubator at 37 °C and 5% CO_2_ unless otherwise stated. Cell lines were passaged by diluting the cells into a culture flask at the desired density in complete media. Cell lines were routinely mycoplasma tested (MycoStrip, Invitrogen) and were found to be negative. TH-302 was synthesized as described previously.^33^

### Hypoxia Treatment

Hypoxic experiments at 0.5–2% O_2_ were carried out in a Whitley H35 Hypoxystation (Don Whitley). For radiobiological hypoxia treatments at <0.1% O_2_, experiments were carried out in a Bactron II anaerobic chamber (Shel laboratories). Cells were fixed inside the chambers for immunofluorescence experiments by using equilibrated solutions. The absence of trace oxygen was periodically verified by using anaerobic indicator strips (Fisher Scientific). To further verify oxygen levels, an OxyLite probe (Oxford Optronix) was used to determine oxygen levels in the media surrounding cells exposed to hypoxia.

### MTT (3-(4,5-Dimethylthiazol-2-yl)-2,5-diphenyl-2*H*-tetrazolium bromide) Assay

Cells were plated in plastic clear-bottomed 96-well plates with complete media up to a total volume of 100 μL/well. Optimal cell seeding number (50,000 cells/well) was obtained from utilizing a standard curve. Three technical repeats were set up for each treatment condition. Cells were incubated with 0.5 mg/mL MTT reagent in complete media for 3 h at 37 °C protected from light.^34^ Once formazan (purple) crystals were visible inside the cells under the microscope, the plate was gently spun for 5 min. MTT-containing media was removed, and formazan crystals were solubilized with 100 μL of DMSO for 15 min at 37 °C protected from light. Absorbance was read immediately at 570 nm by using a POLARstar plate reader. MTT assays for hypoxic samples remained inside the hypoxia chamber until being transferred to the plate reader. Normoxic cell viability data are expressed as percentage viability relative to normoxic vehicle control. Hypoxic cell viability data are expressed as percentage viability relative to hypoxic vehicle control.

### Immunofluorescence and Microscopy

Cells were fixed in 4% (w/v) paraformaldehyde (PFA) for 10 min at rt and washed twice with ice-cold 1× PBS. Fixed cells were treated with TNB (Tris-NaCl-blocking buffer: 0.1 m Tris-HCl, pH 7.5. 0.15 m NaCl, 0.5% (w/v) blocking reagent (PerkinElmer FP1020)) for 1 h at rt. Cells were washed three times with 1× PBS with 0.3% Tween-20 and incubated overnight at 4 °C with prediluted (1:300) anti-EF5 488 antibody (EF55010, Sigma-Aldrich). Cells were washed twice with 1× PBS with 0.3% Tween-20 and a final wash was carried out with 1× PBS. Cells were dried and mounted onto microscopy slides (ThermoFisher Scientific) using mounting medium containing DAPI (ThermoFisher Scientific). Prior to fixing, cells were incubated with 20 μM EF5 (**1**) (2-(2-nitro-1*H*-imidazol-1-yl)-*N*-(2,2,3,3,3-pentafluoropropyl) acetamide). Images were acquired with a 60× objective Zeiss 710 confocal microscope. Images were analyzed using ImageJ-win64.

### Flow Cytometry

Cells were seeded onto glass 6 cm^2^ dishes. Cells were treated with indicated concentrations of probe for 24 h and the indicated oxygen concentration. Cells were collected into a 1.5-mL tube and fixed inside the hypoxia chamber with 4% PFA for 10 min. Samples were washed three times with 1× PBS. Samples were run on a CytoFlex (Beckman Coulter) instrument, and data were analyzed using FloJo software. Filter set used for the detection of DCM (**5**) fluorescence was (APC-A700-A, ex638/em712/25 nm).

### Apoptosis Assay

Apoptosis by nuclear morphology was assessed in cells fixed in 4% paraformaldehyde and stained with DAPI to visualize the nuclei. Apoptotic cells were scored and measured as the percentage of cells with fragmented or condensed nuclei in every treatment. At least 200 cells were counted per condition.

### Quantification and Statistical Analysis

Statistical analysis was performed by using GraphPad Prism 8 software (GraphPad Software Inc.). For the MTT assays and apoptosis data, the two-way ANOVA test was used. Unless otherwise indicated, all data represent the mean ± standard error of the mean (sem) from three independent experiments.

## Supporting Information Available

Supplementary figures, biochemical and chemical experimental details, NMR spectra, HPLC traces (PDF)
