# Supplementary material for: Antibody-Based Imaging of Bioreductive Prodrug Release in Hypoxia
Source: JACS Au. 2023 Nov 1;3(11):3237–46. doi: 10.1021/jacsau.3c00562 (PMC10685431; doi:10.1021/jacsau.3c00562)
Supplement: Supplementary file 1 — au3c00562_si_001.pdf [file au3c00562_si_001.pdf]

Antibody-based Imaging of Bioreductive Prodrug Release in Hypoxia

Çağla Tosun,[†,1] Antoine L. D. Wallabregue,[†,2] Maxim Mallerman,[†,2] Sarah Phillips,[2] Claire M. Edwards,[3,4] Stuart J. Conway*[,2,5] and Ester M. Hammond*[,1]

[1]Department of Oncology, University of Oxford, Old Road Campus Research Building, Oxford, OX3 7DQ, United Kingdom.

[2]Department of Chemistry, Chemistry Research Laboratory, University of Oxford, Mansfield Road, Oxford, OX1 3TA, United Kingdom.

[3]Nuffield Department of Surgical Sciences, University of Oxford, Oxford, OX3 7HE, United Kingdom

[4]Nuffield Department of Orthopaedics, Rheumatology and Musculoskeletal Sciences, University of Oxford, Oxford, OX3 7LD, United Kingdom.

[5]Department of Chemistry & Biochemistry, University of California, 607 Charles E. Young Drive East, Los Angeles, California, CA90095, United States.

[†]These authors contributed equally to this work.

[*]Corresponding authors

Stuart J. Conway

stuartconway@chem.ucla.edu

ORCID – 0000-0002-5148-117X

Ester M. Hammond

ester.hammond@oncology.ox.ac.uk

ORCID – 0000-0002-2335-3146

Çağla Tosun, ORCID – 0009-0001-4025-6866

Antoine L. D. Wallabregue, ORCID – 0000-0002-3181-8636

Claire M. Edwards, ORCID – 0000-0002-1257-5659

Contents

1. Supplementary figures 3

2. Enzymatic reduction of DCM-based probes and biomolecule modification 12

General procedure for the HPLC-based assay of DCM-based and DCM probes 12

Normoxia control procedure for the HPLC-based assay of DCM-based and DCM probes: 12

General procedure for the fluorescence-based assay of DCM-based and DCM probes............13

Normoxia control procedure for the fluorescence-based assay of DCM-based and DCM probes 13

Normoxia control procedure:...14

Normoxia control procedure:...15

3. Chemistry experimental section 16

4. NMR spectra 30

5. HPLC Traces 54

1. Supplementary figures

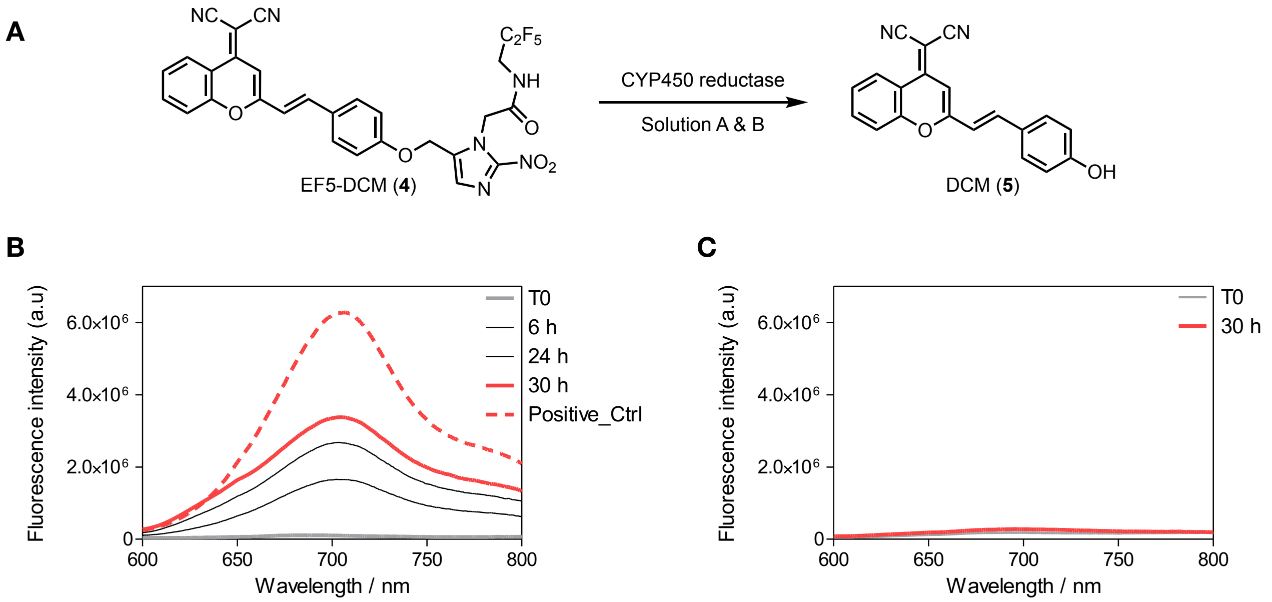

Figure S1. EF5-DCM (4) can be reduced to the corresponding fluorescent reporter DCM (5). (**A**) Enzymatic reduction of compound 5 to 4 using CYP004 and regenerative solutions A & B. (**B**) Compound 4 (50 µM) was treated with NADPH cytochrome P450 reductase (CYP004, C=92 pM) and solution A (100 µL), and solution B (20 µL) under hypoxia (0.1% O_2) over 30 hours, as described in the general procedure for fluorescence-based assay. Fluorescence intensity data were collected after the time indicated in the figure with excitation at 545 nm. Slits 3, 3 nm (a representative graph is shown, n = 3). (**C**) Normoxic (21% O_2) conditions as described in fluorescence-based assay normoxia control (a representative graph is shown, n = 3).

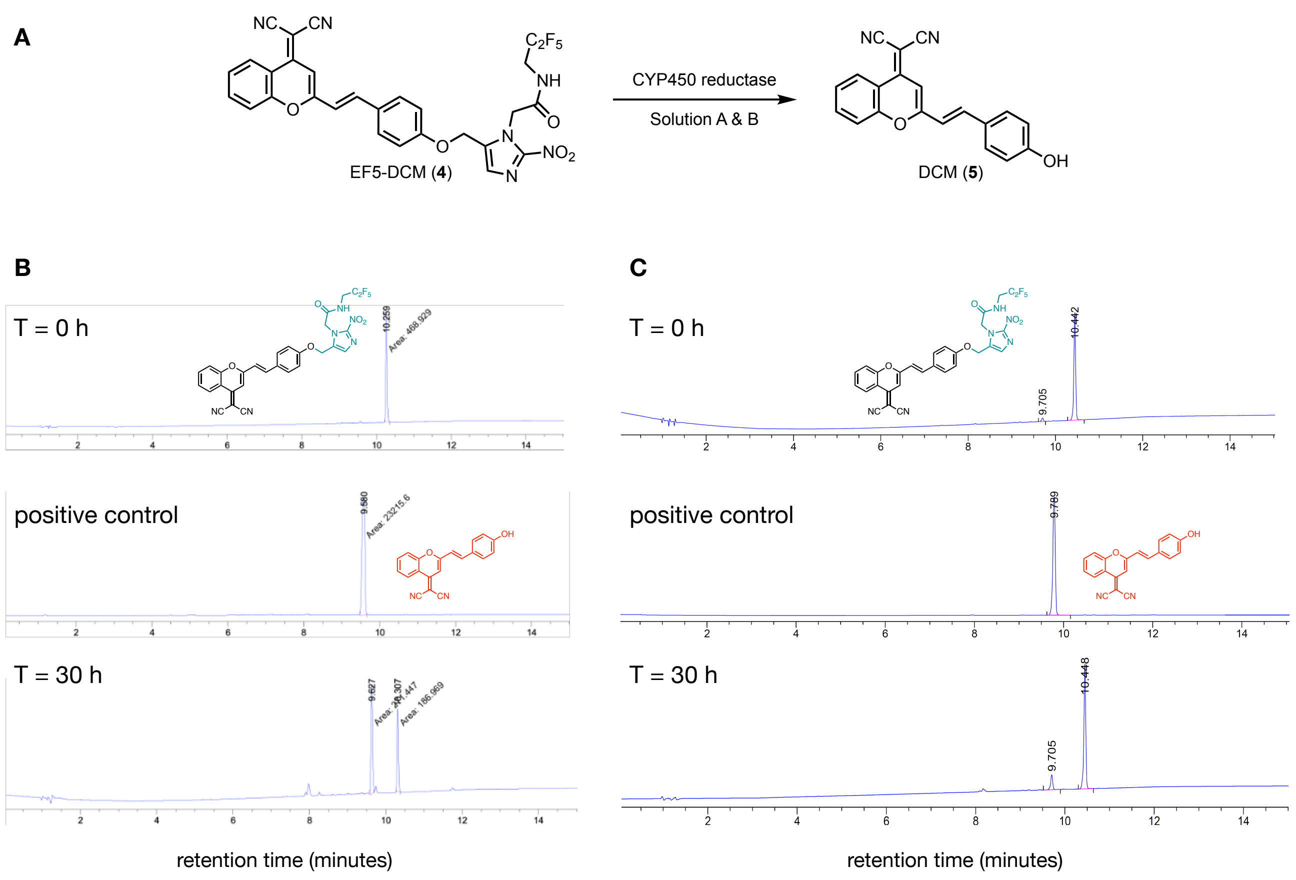

Figure S2. EF5-DCM (4) can be reduced to the corresponding fluorescent reporter DCM (5). (**A**) Enzymatic reduction of compound **4** to **5** using CYP004 and regenerative solutions A & B. (**B**) Compound **4** (50 µM) was treated with NADPH-cytochrome P450 reductase (CYP004, C=92 pM) and solution A (100 µL), and solution B (20 µL) under hypoxia (0.1% O_2) over 30 hours, as described in the general procedure for HPLC-based assay. Aliquots were taken at the time indicated and analyzed by HPLC. Aliquots were taken at T = 0 and T = 30 hours and analyzed using analytical HPLC (see chemistry experimental section for method). Positive control DCM (**5**, 50 µM) was treated as described in the general procedure for HPLC-based assay. Absorbance was recorded at 450 nm. (**C**) Normoxic (21% O_2) conditions as described in HPLC-based assay normoxia control. Aliquots were taken at T = 0 and T = 30 hours and analyzed using analytical HPLC (see chemistry experimental section for method). Positive control DCM (**5**, 50 µM) was treated as described in the general procedure for HPLC-based assay. Absorbance was recorded at 450 nm.

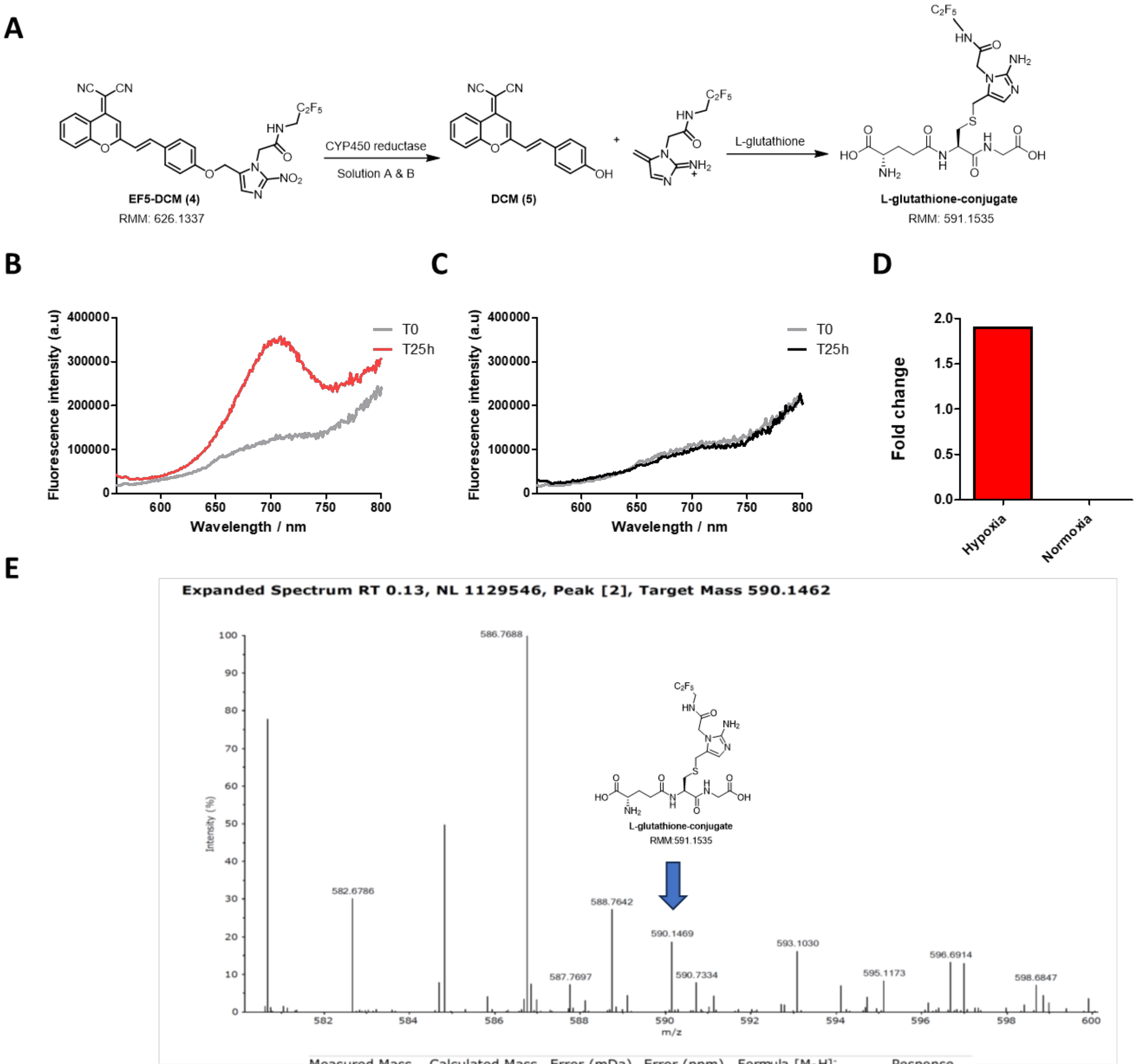

Measured Mass	Calculated Mass	Error (mDa)	Error (ppm)	Formula [M-H]$^-$	Response
590.1469	590.1462	0.72	1.22	$C_{19}H_{25}F_5N_7O_7S$	15578

Figure S3. EF5-DCM (4) can be reduced to the corresponding fluorescent reporter DCM (5) and alkylate L-Gluthatione. (**A**) The enzymatic reduction of EF5-DCM (4) to give DCM (5) and the proposed iminium intermediate that react with L-glutathione (GSH) to give the L-glutathione conjugate. (**B**) Compound **4** (50 μM) was treated with NADPH-cytochrome P450 reductase (CYP004, C=92 pM) and solution A (100 μL), and solution B (20 μL) in hypoxia (0.1% O_2) over 25 h, as described in the general procedure for fluorescence-based assay. Fluorescence intensity data were collected after the time shown, with excitation at 545 nm. Slits 3, 3 nm (a representative graph is shown, n = 2). (C) Normoxic (21% O_2) conditions as described in fluorescence-based assay normoxia control (a representative graph is shown, n = 2). (**D**) Quantification of the fluorescence increase shown by compound 5, showing a 1.9% increase in fluorescence after 25 h under hypoxia whereas the fluorescence of normoxia control is 0%. (**E**) HRMS spectrum (negative mode) of an aliquot (500 μL) taken after 25 h, showing the [M-H]$^-$ peaks for the L-glutathione-conjugate formed after enzymatic reduction and treatment with GSH in hypoxic conditions

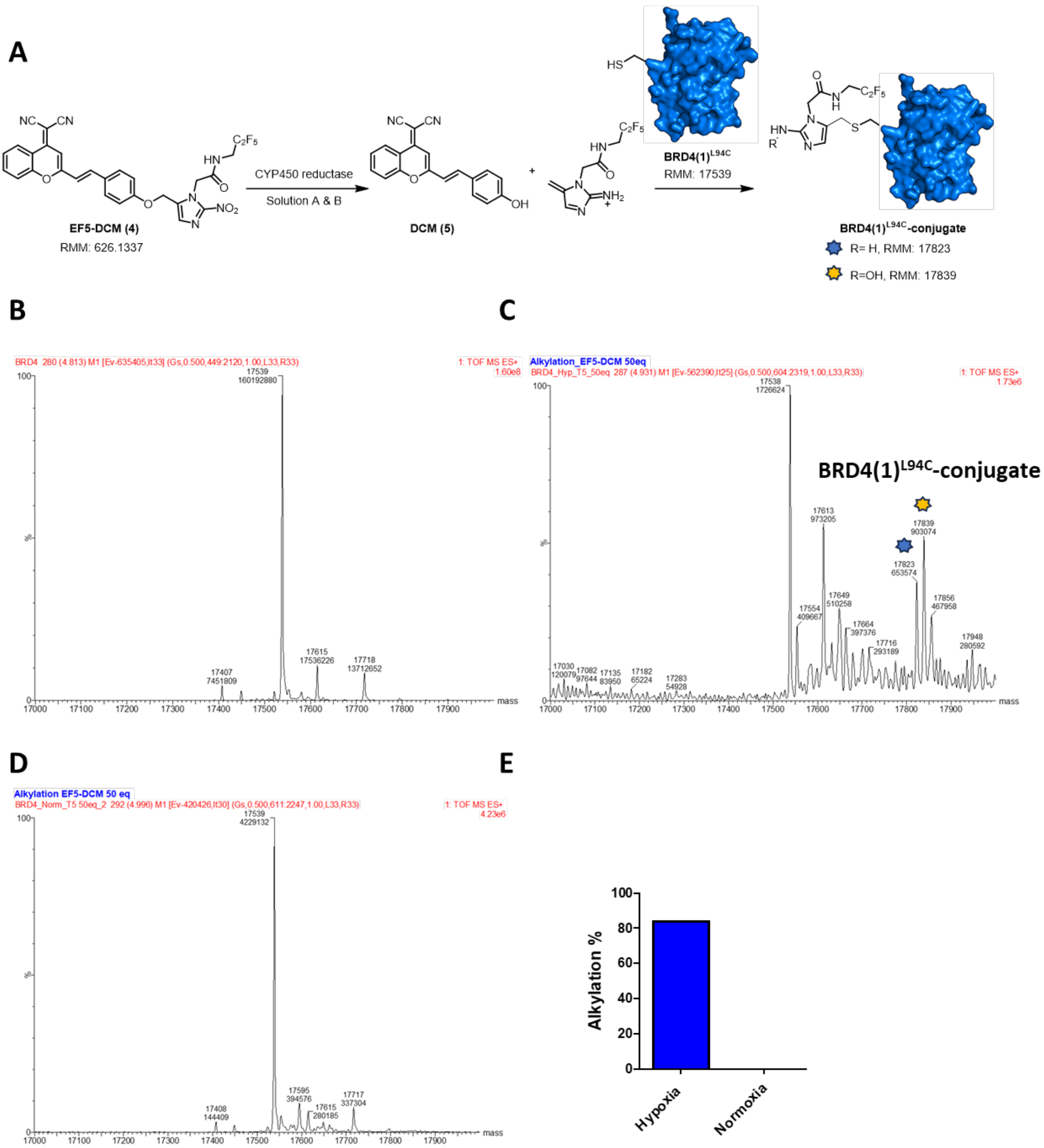

Figure S4. EF5-DCM (4) can be reduced to the corresponding fluorescent reporter DCM (5) and alkylate a cysteine-containing protein (BRD4(1)^{L94C}. (**A**) The enzymatic reduction of EF5-DCM (4) to give DCM (5) and the proposed iminium intermediate that reacts with (BRD4(1)^{L94C} to give (BRD4(1)^{L94C} conjugate. (**B**) Deconvoluted protein mass spectrum of (BRD4(1)^{L94C}. (**C**) Deconvoluted protein mass spectrum of an aliquot taken after 5 h from the following reaction: EF5-DCM (500 μM) and protein (BRD4(1)^{L94C} (10 μM) were treated with NADPH-cytochrome P450 reductase (CYP004, C=9.2 pM) and solution A (10 μL), and solution B (2 μL) under hypoxia (0.1% O_2), as described in the general procedure for protein modification. Then LC-MS data were collected as described by intact protein mass spectrometry procedure (a representative graph is shown, n = 2). Spectrum showing the peaks of unmodified (BRD4(1)^{L94C} and labelled (BRD4(1)^{L94C}. (**D**) Deconvoluted protein mass spectrum of an aliquot taken after 5 h from the normoxic (21% O_2)

conditions as described in protein modification normoxia control. LC-MS data were collected (a representative graph is shown, n = 2). (**E**) Quantification of (BRD4(1)^{L94C} alkylation: (.BRD4(1)^{L94C} conjugate peak intensity /(BRD4(1)^{L94C}) × 100. n=2. 84% protein alkylation (32% and 52% as amine (blue star) and hydroxylamine products (yellow star), respectively) was observed after 5 h under hypoxia whereas the intensity of normoxia control is 0%.

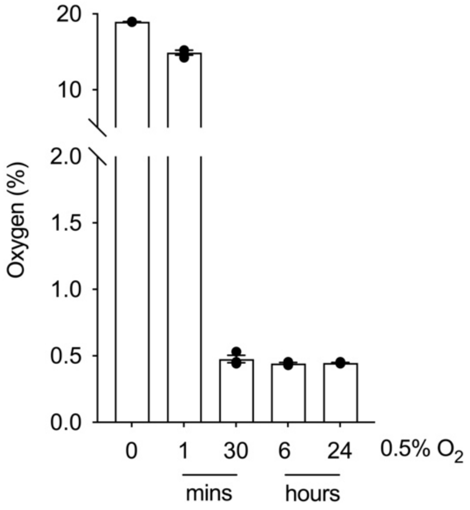

Figure S5: Measurement of aqueous concentration of oxygen inside the hypoxia chamber set at 0.5% O_2. MM.1S cells were exposed to 0.5% O_2 and level of dissolved oxygen in media was monitored using an oxygen probe (OxyLite) at indicated timepoints (n=3).

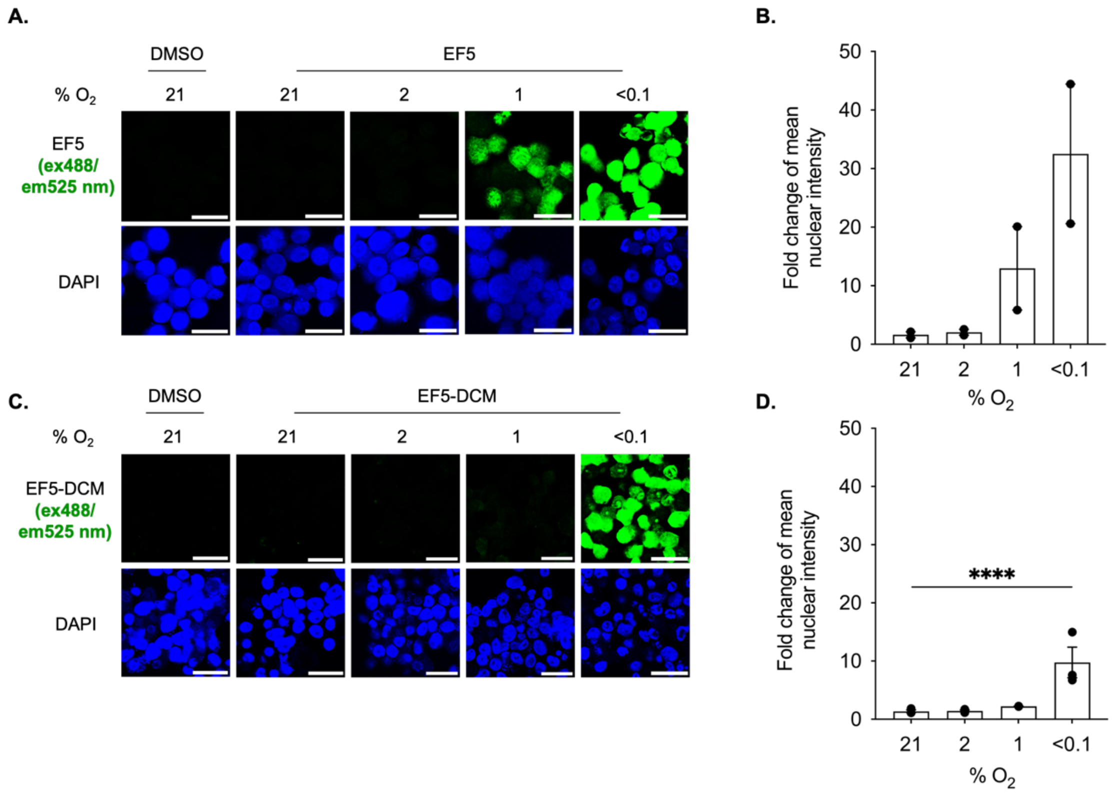

Figure S6: Comparing the oxygen dependency of EF5 (1) and EF5-DCM (4) bioreduction. A. MM.1S cells were exposed to 24 hours of either normoxia or the indicated levels of hypoxia (<0.1%, 1% or 2% O_2) in the presence of EF5 (70 μM). Cells were fixed and stained for DAPI and EF5. Scale bars represent 20 μm. **B.** Quantification of EF5 fluorescence from panel **A** at each oxygen concentration. Each dot represents the mean of a biological repeat where the increase in fluorescence was normalized to the vehicle control (n=2). Minimum of 300 cells were imaged per treatment. **C.** MM.1S cells were exposed to 24 hours of either normoxia or the levels of hypoxia indicated (<0.1%, 1% or 2% O_2) in the presence of EF5-DCM (10 μM). Cells were fixed and stained for DAPI and EF5. Scale bars represent 20 μm. **D.** Quantification of EF5 fluorescence from panel **C** at each oxygen concentration. Each dot represents the average of a biological repeat where the increase in fluorescence was normalized to the vehicle control. Minimum of 300 cells were imaged per treatment. Significance student's t-test. ****: $p<0.00005$ (n=3).

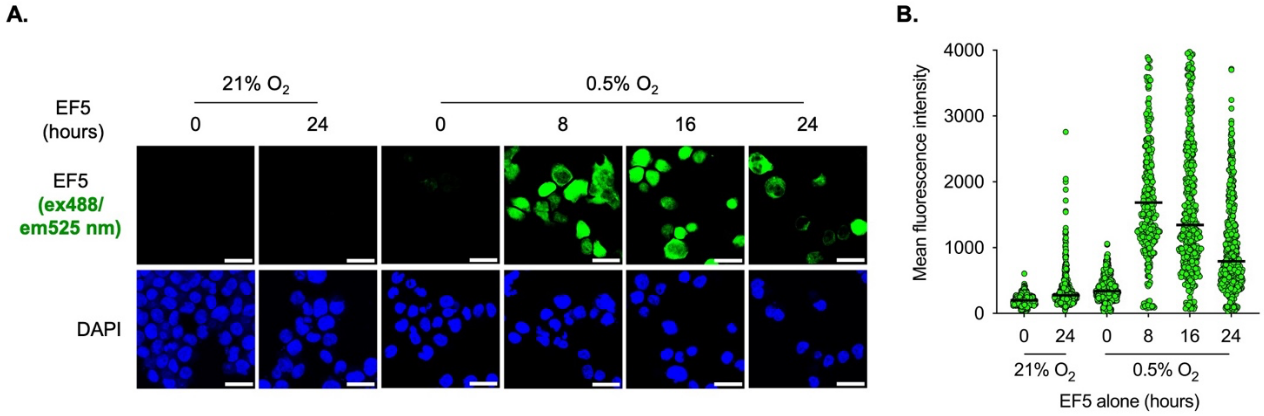

Figure S7: EF5 bioreduction is detected as soon as 8 hours in 0.5% O_2. A. MM.1S cells were treated with EF5 alone (40 µM) and exposed to 0.5% O_2 for indicated timepoints. Scale bar indicates 20 µm. **B.** Data from a representative experiment where each dot represents a cell (minimum of 200 cells per treatment). Black lines represent average fluorescence (n=3).

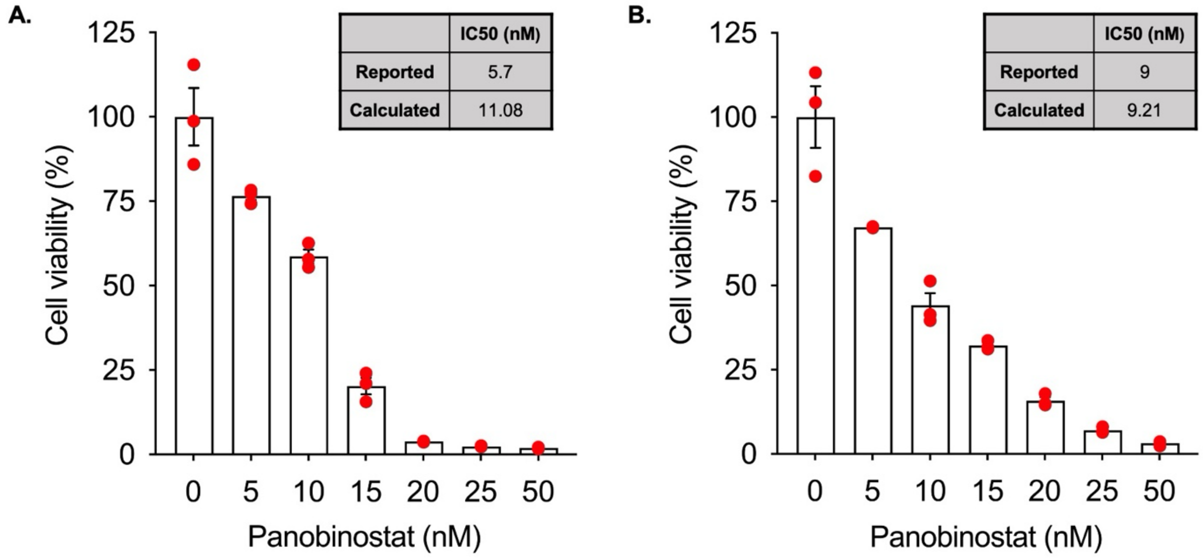

Figure S8: Panobinostat leads to loss of cell viability in MM cell lines. A. MM.1S and **B.** JJN3 cells were treated with indicated concentrations of panobinostat for 24 hours. Cell viability was assessed using an MTT assay. Reported and calculated IC50 values for each cell line is shown. Data are mean ± s.e.m (n=1).

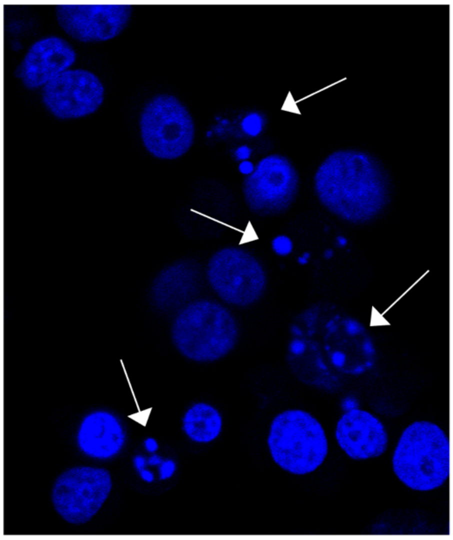

Figure S9: Examples of the change in nuclear morphology observed during apoptosis. JJN3 cells were exposed to hypoxia (0.5% O_2) in the presence of EF5-Pano (10 μM) for 24 hours followed by DAPI staining. The white arrows indicate cells which were scored as apoptotic.

2. Enzymatic reduction of DCM-based probes and biomolecule modification

The oxygen-dependent enzymatic reduction assays were run in a RUSKINN InvivO2® 400 hypoxic Workstation at 37 °C with 0.1% oxygen and 99.9% nitrogen concentrations unless otherwise stated. The human NADPH-cytochrome P450 reductase (CYP004, expressed in *Escherichia coli*, 4 nmol) was purchased from Cypex®. The NADPH-regenerating system (solution A 451220 and solution B 451200) and the phosphate buffer (0.5 M, pH 7.4) were purchased from Corning®. The buffers used in the assays were equilibrated under the conditions stated above in the hypoxic Workstation for 18 hours before use. HPLC and/or fluorimetry were used as readouts for reaction analysis.

General procedure for the HPLC-based assay of DCM-based and DCM probes: To a 2 mL Eppendorf tube containing potassium phosphate buffer (0.5 M, 400 µL, pH 7.4) and deionized water (1448.6 µL) was added a solution of probe (DCM-based probe or DCM) (10 µL, of a 10 mM stock solution in DMSO). An aliquot (500 µL) was collected from the reaction solution and filtered using a syringe filter (Gilson PTFE-4-4, size × porosity: 13 mm × 0.20 µm). The filter was washed with acetonitrile (200 µL) and the flow-through was combined with the aliquot and analyzed using HPLC analysis (this sample serves as T = 0, where T = time). Next, the human NADPH-cytochrome P450 reductase (19.4 µL of a 10.3 nm/mL solution), solution A (100 µL), and solution B (20 µL) were added to the reaction mixture in the hypoxia chamber, and the Eppendorf tube was left in the hypoxia chamber. The final concentrations of human NADPH-cytochrome P450 reductase and probe were approximatively 92 pM and 50 µM, respectively. An aliquot (500 µL) was collected at the indicated time, passed through a syringe filter, and the filter washed with acetonitrile (200 µL). The flow-through was combined with the aliquot and analyzed using HPLC or LCMS.

Normoxia control procedure for the HPLC-based assay of DCM-based and DCM probes: To a 2 mL Eppendorf tube containing potassium phosphate buffer (0.5 M, 400 µL, pH 7.4) and deionized water (1448.6 µL) was added a solution of probe (DCM-based probe or DCM) (10 µL, of a 10 mM stock solution in DMSO). An aliquot (500 µL) was collected from the reaction solution and passed through a syringe filter (Gilson PTFE-4-4, size × porosity: 13 mm × 0.20 µm). The filter was washed

with acetonitrile (200 µL) and the flow-through was combined with the aliquot and analyzed by HPLC (this sample serves as T = 0, where T = time). Next, the human NADPH-cytochrome P450 reductase (19.4 µL of a 10.3 nm/mL solution), solution A (100 µL), and solution B (20 µL) were added to the reaction mixture, and the Eppendorf was left at room temperature, under ambient conditions. The final concentrations of human NADPH-cytochrome P450 reductase and probe were approximatively 92 pM and 50 µM, respectively. An aliquot (500 µL) was collected at the indicated time, passed through a syringe filter, and the filter washed with acetonitrile (200 µL). The flow-through was combined with the aliquot and analyzed using HPLC or LCMS.

General procedure for the fluorescence-based assay of DCM-based and DCM probes: To a 2 mL Eppendorf tube containing potassium phosphate buffer (0.5M, 400 µL, pH 7.4) and deionized water (1448.6 µL) was added a solution of probe (DCM-based probe or DCM) (10 µL, of a 10 mM stock solution in DMSO). An aliquot (500 µL) was collected from the reaction solution and added to a 3 mL quartz cuvette. DMSO (1.5 mL) and deionized water (1 mL) were added, and the fluorescence spectra were recorded according to the indicated parameters (this sample serves as T = 0, where T = time). Next, the human NADPH-cytochrome P450 reductase (19.4 µL of a 10.3 nm/mL solution), solution A (100 µL), and solution B (20 µL) were added to the reaction mixture in the hypoxia chamber, and the Eppendorf tube was left in the hypoxia chamber. The final concentrations of the human NADPH-cytochrome P450 reductase and probe were approximately 92 pM and 50 µM, respectively. An aliquot (500 µL) was collected at the indicated time, added to a 3 mL quartz cuvette, and diluted with DMSO (1.5 mL) and deionized water (1 mL), and the fluorescence spectrum was recorded.

Normoxia control procedure for the fluorescence-based assay of DCM-based and DCM probes: To a 2 mL Eppendorf tube containing potassium phosphate buffer (0.5M, 400 µL, pH 7.4) and deionized water (1448.6 µL) was added a solution of probe (DCM-based probe or DCM) (10 µL, of a 10 mM stock solution in DMSO). An aliquot (500 µL) was collected from the reaction solution and added to a 3 mL quartz cuvette. DMSO (1.5 mL) and deionized water (1 mL) were added, and the fluorescence spectra were recorded using the parameters shown in Figure S3 (this sample serves as T = 0, where T = time). Next, the human NADPH-cytochrome P450 reductase (19.4 µL of

a 10.3 nm/mL solution), solution A (100 µL), and solution B (20 µL) were added to the reaction mixture, and the Eppendorf was left at room temperature, under ambient conditions. The final concentrations of the human NADPH-cytochrome P450 reductase and probe were approximately 92 pM and 50 µM, respectively. An aliquot (500 µL) was collected at the indicated time, added to a 3 mL quartz cuvette, and diluted with DMSO (1.5 mL) and deionized water (1 mL), and the fluorescence spectrum was recorded.

L-Glutathione modification: To a 2 mL Eppendorf tube containing potassium phosphate buffer (0.5 M, 400 µL, pH 7.4) and deionized water (1438.6 µL) was added a solution of EF5-DCM probe (10 µL, of a 10 mM stock solution in DMSO) and a solution of L-glutathione (10 µL, of a 100 mM stock solution in deionized water). An aliquot (500 µL) was collected from the reaction solution and added to a 3 mL quartz cuvette. DMSO (1.5 mL) and deionized water (1 mL) were added, and the fluorescence spectra were recorded according to the indicated parameters (this sample serves as T = 0, where T = time). Next, the human NADPH-cytochrome P450 reductase (19.4 µL of a 10.3 nm/mL solution), solution A (100 µL), and solution B (20 µL) were added to the reaction mixture in the hypoxia chamber, and the Eppendorf tube was left in the hypoxia chamber. The final concentrations of the human NADPH-cytochrome P450 reductase and probe were approximatively 92 pM and 50 µM, respectively. Two aliquots (2 × 500 µL) were collected at the indicated time, one was added to a 3 mL quartz cuvette, and diluted with DMSO (1.5 mL) and deionized water (1 mL), and the fluorescence spectrum was recorded, the other was analyzed by HRMS.

Normoxia control procedure: To a 2 mL Eppendorf tube containing potassium phosphate buffer (0.5 M, 400 µL, pH 7.4) and deionized water (1438.6 µL) was added a solution of EF5-DCM probe (10 µL, of a 10 mM stock solution in DMSO) and a solution of L-glutathione (10 µL, of a 100 mM stock solution in deionized water). An aliquot (500 µL) was collected from the reaction solution and added to a 3 mL quartz cuvette. DMSO (1.5 mL) and deionized water (1 mL) were added, and the fluorescence spectra were recorded according to the indicated parameters (this sample serves as T = 0, where T = time). Next, the human NADPH-cytochrome P450 reductase (19.4 µL of a 10.3 nm/mL solution), solution A (100 µL), and solution B (20 µL) were added to the reaction mixture and the Eppendorf was left at room temperature, under ambient conditions. The final concentrations of the

human NADPH-cytochrome P450 reductase and probe were approximatively 92 pM and 50 μM, respectively Two aliquots (2 × 500 μL) were collected at the indicated time, one was added to a 3 mL quartz cuvette, and diluted with DMSO (1.5 mL) and deionized water (1 mL), and the fluorescence spectrum was recorded, the other was analyzed by HRMS.

Protein modification: To a 1.5 mL Eppendorf tube containing potassium phosphate buffer (0.5 M, 40 μL, pH 7.4) and deionized water (129.6 μL) was added a solution of EF5-DCM probe (10 μL, of a 10 mM stock solution in DMSO), followed by the BRD4(1)^{L94C} protein (6.6 μL of a 302 μM solution), the human NADPH-cytochrome P450 reductase (1.94 μL of a 10.3 nm/mL solution), solution A (10 μL), and solution B (2 μL) were added to the reaction mixture in the hypoxia chamber, the final concentrations of BRD4(1)^{L94C} and EF5-DCM probe were approximately 10 μM and 500 μM. An aliquot (50 μL) was collected from the reaction solution and analyzed using HPLC analysis (this sample serves as T = 0, where T = time). Then, the Eppendorf tube was left in the hypoxia chamber. An aliquot (500 μL) was collected at the indicated time, analyzed using LCMS and intact protein mass spectrometry.

Normoxia control procedure: To a 1.5 mL Eppendorf tube containing potassium phosphate buffer (0.5 M, 40 μL, pH 7.4) and deionized water (129.6 μL) was added a solution of EF5-DCM probe (10 μL, of a 10 mM stock solution in DMSO), followed by the BRD4(1)^{L94C} protein (6.6 μL of a 302 μM solution), the human NADPH-cytochrome P450 reductase (1.94 μL of a 10.3 nm/mL solution), solution A (10 μL), and solution B (2 μL) were added to the reaction mixture at room temperature, under ambient conditions, the final concentrations of BRD4(1)^{L94C} and EF5-DCM probe were approximately 10 μM and 500 μM. An aliquot (50 μL) was collected from the reaction solution and analyzed using HPLC analysis (this sample serves as T = 0, where T = time). Then, the Eppendorf tube was left at 37 °C, under ambient conditions. An aliquot (500 μL) was collected at the indicated time, analyzed using LCMS and intact protein mass spectrometry method.

Intact Protein Mass Spectrometry: LC–MS method for analysis of protein conjugation. LC–MS was carried out on a Waters Xevo G2-XS QTOF mass spectrometer, equipped with a Waters Acquity UPLC. A ProSwift™ RP-2H monolithic 4.6 mm × 50 mm column (Thermo Scientific™) was used with

a constant flow rate of 0.4 mL/min and a gradient method of 10 min from H_2O:MeCN (95:5) to H2O:MeCN (5:95) with a 5 min hold. All samples were run with 0.1% formic acid in the elution solvents. Mass spectra were deconvoluted using Waters MassLynx™ software (MaxEnt1 algorithm, 10–20 kDa range, 1.0 Da resolution).

3. Chemistry experimental section

Chemicals were purchased from Sigma Aldrich UK, Alfa Aesar UK, and Fluorochem, and were used as supplied unless stated. Brine refers to a saturated aqueous solution of sodium chloride. Anhydrous solvents were obtained from an MBRAUN Solvent Purification System 5 and stored under an argon atmosphere over 3 Å molecular sieves. Petroleum ether refers to the fraction of light petroleum ether boiling in the range of 40–60 °C. *In vacuo* refers to the removal of solvent using a Buchi® rotary evaporator under reduced pressure in a water bath at 40 °C.

^{1}H NMR spectra were recorded on Bruker AVC500 (500 MHz) or Bruker AVH400 (400 MHz) spectrometers using deuterochloroform (unless indicated otherwise) as a reference for the internal deuterium lock. The chemical shift data for each signal are given as δH in units of parts per million (ppm) relative to tetramethylsilane (TMS) where δH (TMS) = 0.00 ppm. The multiplicity of each signal is indicated by s (singlet); d (doublet); t (triplet); q (quartet); dd (doublet of doublets); dt (doublet of triplets) or m (multiplet). The number of protons (n) for a given resonance signal is indicated by nH. Coupling constants (*J*) are quoted in Hz and are recorded to the nearest 0.1 Hz. Identical proton coupling constants (*J*) are averaged in each spectrum and reported to the nearest 0.1 Hz. The coupling constants were determined by analysis using MestreNova software. Bruker Topspin was used to plot the spectra. Spectra were assigned using COSY, NOESY, HSQC, and HMBC experiments, as necessary.

^{13}C NMR spectra were recorded on a Bruker AVC500 (126 MHz) or AVH400 (100 MHz) spectrometers in the stated solvents, with broadband proton decoupling and an internal deuterium lock. The chemical shift data for each signal are given as δC in units of parts per million (ppm) relative

to tetramethylsilane (TMS) where δC (TMS) = 0.00 ppm. The shift values of resonances are quoted to 1 decimal place and were determined using Bruker MestreNova software. Bruker Topspin was used to plot the spectra. Spectra were assigned using HSQC and HMBC experiments, as necessary.

Mass spectra were acquired on a VG platform spectrometer and an Agilent 6120 spectrometer (low resolution). Electro-spray ionization spectra were obtained from solutions of MeOH or MeCN using Micromass LCT Premier or Bruker MicroTOF spectrometers, operating in a positive or negative mode, as indicated. *m/z* values are reported in Daltons and followed by their percentage abundance in parentheses. Electron ionization/field ionization (EI/FI) was carried out on a Waters GCT with a temperature-programmed solids probe inlet. Samples were introduced in glass tips directly into the source where they were vaporized and analyzed. In EI the ionization is by electron impact, electrons being provided by a filament. In FI the ionization is in an intense electric field which causes quantum electron tunneling of a valence electron.

Melting points were determined using a Griffin capillary tube melting point apparatus and are uncorrected.

Infrared spectra were obtained from neat samples, either as solids or liquids, using a diamond ATR module. The spectra were recorded on a Bruker Tensor 27 spectrometer. Absorption maxima are recorded in wavenumbers (cm^{-1}) and reported as s (strong), m (medium), w (weak), or br (broad).

Analytical thin-layer chromatography (TLC) was carried out on normal-phase Merck silica gel 60 F254 aluminum-supported chromatography sheets. Visualization was achieved using absorption of UV light (λ_{max} 254 and 365 nm) or thermal development after staining in an aqueous solution of potassium permanganate. UV light was provided by a LF – 206.LS 230V – 50 Hz lamp from UVItec Limited.

Flash column chromatography was performed manually using Geduran® silica gel 60 (40-63 μm) eluting with solvents as supplied, under a positive pressure of air or nitrogen, or on a Biotage® Selekt Flash Pure Purification System using Biotage® KP-Sil SNAP or Biotage® Sfär Silica cartridges.

Fluorescence spectroscopy: HORIBA Jobin Yvon FluoroLog3 fluorimeter (Hamamatsu R928 detector and a double-grating emission monochromator) was used to acquire the fluorescence spectra. The standard conditions for acquiring emission and excitation spectra are room temperature and steady-stated mode unless otherwise stated. HORIBA Jobin Yvon FluoroLog3 fluorimeter system equipped with a Xenon flash lamp was used to acquire emission lifetimes. Fluorescence spectra were plotted using GraphPad Prism 8 software (GraphPad Software Inc.).

UV-vis spectra were recorded on a V-770 UV-Vis/NIR Spectrophotometer equipped with a Peltier temperature controller and stirrer, using disposable polystyrene cuvettes of 1 cm path length. Experiments were conducted at 25 °C unless otherwise stated. UV-vis spectra were plotted using Prism 8 software (GraphPad Software Inc.).

Semi-preparative high performance liquid chromatography (HPLC) method: was carried out on an Agilent 1260 Infinity II® system equipped with an Agilent 5 Prep C18 column [5 μM, 21.2 × 50 mm]; [95:5 H_2O:MeCN 0.1% formic acid (FA) modifier (1 min), 95:5 H_2O:MeCN → 5:95 H_2O: MeCN with 0.1% FA modifier (10 min), 5 min hold; 20 mL min^{-}1]

Analytical high performance liquid chromatography (HPLC) method: was carried out on an Agilent 1260 Infinity II® system equipped with a Poroshell 120 EC-C18 column [4 μm, 4.6 × 100 mm]; [95:5 H_2O:MeCN 0.1% FA modifier (1 min), 95:5 H_2O: MeCN → 5:95 H_2O: MeCN: H_2O with 0.1% FA modifier (10 min); 5 min hold, 95:5 H_2O:MeCN % FA modifier (1 min); 1 mL min^{-1}].

Ethyl imidazo[1,2-a]pyrimidine-3-carboxylate (8) and ethyl imidazo[1,2-a]pyrimidine-2-carboxylate (9)

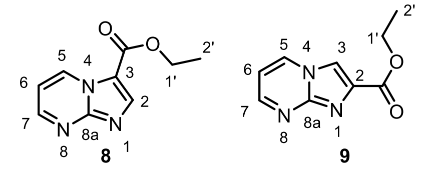

8 **9**

A solution of 2-aminopyrimidine (2.50 g, 26.3 mmol, 1.0 eq.) and ethyl bromopyruvate (3.68 mL, 26.3 mmol, 1.0 eq.) in ethanol (40 mL) was heated to 75 °C for 16 h. After TLC analysis, the reaction mixture was concentrated *in vacuo*, then redissolved in dichloromethane (30 mL) and washed with saturated with aqueous sodium hydrogen carbonate (30 mL). The aqueous layer was extracted with dichloromethane (2 × 25 mL), and the organic components were combined, dried (sodium sulfate), filtered, and concentrated *in vacuo*. Purification using silica gel chromatography, eluting with ethyl acetate yielded ethyl imidazo[1,2-a]pyrimidine-3-carboxylate (**8)** (1.14 g, 5.97 mmol, 47%) as a pale-yellow solid. R_f 0.11 (100% ethyl acetate); m.p. 170–174 C (from ethyl acetate) [lit. 179–180 °C (from ethanol)];[1] ^{1}H NMR (400 MHz, $CDCl_3$) δ 8.67 (1H, dd, *J* 4.1, 2.1, H-7), 8.53 (1H, dd, *J* 6.9, 2.1, H-5), 8.16 (1H, s, H-3), 6.97 (1H, dd, *J* 6.9, 4.1, H-6), 4.46 (2H, q, *J* 7.1, H-1’), 1.42 (3H, t, *J* 7.1, H-2’); LRMS *m/z* (ESI$^+$) 214 ($[M+Na]^+$, 100%). The reaction also yielded ethyl imidazo[1,2-a]pyrimidine-2-carboxylate (**9**, 1.14 g, 5.97 mmol, 47%) as an orange solid. R_f 0.34 (100% ethyl acetate); m.p. 115–117 °C (from ethyl acetate) [lit. 116–118 °C (from ethyl acetate)];[2] ^{1}H NMR (400 MHz, $CDCl_3$) δ 9.55 (1H, dd, *J* 6.9, 2.1, H-7), 8.72 (1H, dd, *J* 4.2, 2.1, H-5), 8.44 (1H, s, H-2), 7.10 (1H, dd, *J* 6.9, 4.2, H-6), 4.42 (2H, q, *J* 7.2, H-1’), 1.41 (3H, t, *J* 7.2, H-2’); LRMS *m/z* (ESI$^+$) 192 ($[M+H]^+$, 100%). The spectroscopic data are in good agreement with the literature values.[1,2]

Ethyl 2-Amino-1*H*-imidazole-5-carboxylate (10)

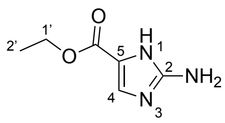

To a solution of **8** (1.13 g, 5.97 mmol, 0.5 eq.) and **9** (1.13 g, 5.97 mmol, 0.5 eq.) in anhydrous ethanol (90 mL), was added hydrazine monohydrate (0.645 mL, 13.3 mmol, 1.1 eq.). The solution

was heated under reflux for 16 h and monitored by TLC analysis, then concentrated *in vacuo*. After, the resulting solid was crystallized from methanol, yielding ethyl 2-amino-1*H*-imidazole-4-carboxylate (**10**) as an orange solid (1.86 g, 12.0 mmol, 90%). R_f 0.15 (10% methanol in dichloromethane); m.p. 162–164 °C (from methanol) [lit. 166–168 °C (from ethyl acetate)];[3] ^{1}H NMR (400 MHz, MeOD) δ 7.24 (1H, s, H-4), 4.24 (2H, q, *J* 7.1, H-1'), 1.31 (3H, t, *J* 7.1, H-2'); LRMS *m/z* (ESI$^-$) 154 ([M−H]$^-$, 100%). The spectroscopic data are in good agreement with the literature values.[3]

2-Chloro-*N*-(2',2',3',3',3'-pentafluoropropyl) acetamide (11)

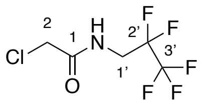

To a solution of chloroacetic acid (1.52 g, 16.2 mmol, 1.0 eq.) in anhydrous tetrahydrofuran (150 mL) at 0 ºC was added *N*-methyl morpholine (1.80 mL, 16.2 mmol, 1.0 eq.). After 10 min, *iso*-butyl chloroformate (2.27 mL, 17.4 mmol, 1.1 eq.) was added. After a further 20 min, 2,2,3,3,3-pentafluoropropan-1-amine (2.00 mL, 16.2 mmol, 1.0 eq.) was added, and the solution was warmed to rt and stirred for 16 h. The precipitate was removed by vacuum filtration, washed with tetrahydrofuran (20 mL), and the filtrate was concentrated *in vacuo*. Purification using silica gel chromatography, eluting with 80% ethyl acetate in petroleum ether yielded 2-chloro-*N*-(2',2',3',3',3'-pentafluoropropyl) acetamide (**11**, 3.09 g, 13.8 mmol, 85%) as a colorless solid. R_f 0.25 (80% ethyl acetate in petroleum ether); m.p. 42–43 °C (from petroleum ether) [lit. 44–45 °C (from tetrahydrofuran)];[4] ^{1}H NMR (400 MHz, $CDCl_3$) δ 6.85 (1H, s, N-H), 4.14 (2H, s, H-2), 4.02 (2H, td, *J* 15.2, 6.0, H-1'); ^{19}F NMR (565 MHz, MeOD) δ −85.91 (3F, s, CF_3), −122.89 (2F, t, $J_{F\text{–}H}$ 15.2, CF_2); LRMS *m/z* (ESI$^-$) 224 ([M−H]$^-$, 100%). The spectroscopic data are in good agreement with the literature values.[4]

Ethyl 2-amino-1-{2'-oxo-2'-[(2'',2'',3'',3'',3''-pentafluoropropyl)amino]ethyl}-1*H*-imidazole-5-carboxylate (12)

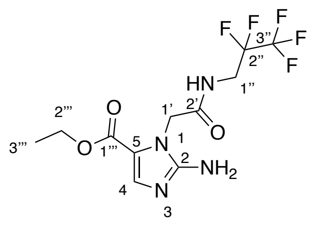

To a solution of **10** (409 mg, 2.63 mmol, 1.0 eq.) in anhydrous *N,N*-dimethylformamide (115 mL) was added cesium carbonate (859 mg, 2.63 mmol, 1.0 eq.), and the suspension was stirred at 50 ºC for 2 h. Compound **11** (600 mg, 2.63 mmol, 1.0 eq.) in *N,N*-dimethylformamide (5 mL) was then added and the suspension stirred for a further 3 h. The precipitate was removed by vacuum filtration, washed with ethyl acetate (30 mL), and the filtrate was concentrated *in vacuo*. The residue was redissolved in methanol (23 mL) and acetic acid (3 mL), and adjusted to pH 9, using an aqueous solution of NaOH (2 M, 9 mL). The resulting precipitate was collected by vacuum filtration, then dissolved in toluene (30 mL) and the solvent was subsequently removed *in vacuo*. This procedure was repeated with heptane (30 mL), to yield *ethyl 2-amino-1-{2'-oxo-2'-[(2'',2'',3'',3'',3''-pentafluoropropyl)amino]ethyl}-1H-imidazole-5-carboxylate* (**12**, 410 mg, 1.19 mmol, 45%) as a pale-yellow solid. R_f 0.49 (50/8/1 dichloromethane/ethanol/ammonium hydroxide); m.p. 230–234 °C (from methanol); $\tilde{\nu}_{max}$ (solid)/cm^{-1} 3395 (N-H, m), 3335 (N-H, m), 2981 (C-H, w), 1661 (C=O, s), 1568 (N-H, m), 1545 (N-H, m), 1177 (s), 1152 (s), 1110 (s); ^{1}H NMR (400 MHz, MeOD) δ 7.38 (1H, s, H-4), 4.89 (2H, s, H-1'), 4.22 (2H, q, *J* 7.1, H-2'''), 4.00 (2H, t, J_{H-F} 15.2, H-1''), 1.30 (3H, t, *J* 7.1, H-3'''); ^{13}C (151 MHz, MeOD) δ 170.6 (C-2'), 161.8 (C-1'''), 155.7 (C-2), 135.9 (C-4), 121.2 (qt, J_{C-F} 285.1, 35.4, C-3''), 119.2 (C-5), 114.5 (tq, J_{C-F} 253.5, 37.1, C-2''), 61.0 (C-2'''), 47.1 (C-1'), 39.6 (t, J_{C-F} 24.5, C-1''), 14.6 (C-3'''); ^{19}F NMR (565 MHz, MeOD) δ –85.91 (3F, s, CF_3), –122.89 (2F, t, J_{F-H} 15.3, CF_2; HRMS *m/z* (ESI$^+$) $C_{11}H_{14}F_5N_4O_3^+$ requires 345.0981, found 345.0981; LRMS *m/z* (ESI$^+$) 689 ([2M+H]$^+$, 100%); HPLC retention time 6.4 min, 99%.

Ethyl 2-nitro-1-{2'-oxo-2'-[(2",2",3",3",3"-pentafluoropropyl)amino]ethyl}-1*H*-imidazole-5-carboxylate (13)

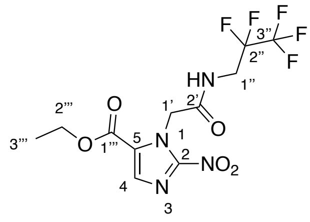

To a solution of **12** (146 mg, 0.424 mmol, 1.0 eq.) in acetic acid (1.5 mL), at 0 ºC, and under argon was added a solution of $NaNO_2$ (293 mg, 4.24 mmol, 10.0 eq.) in water (0.8 mL). The resulting reaction mixture was stirred at 0 ºC for 1.5 h, then warmed to RT, and stirred for 5 h. After this time, the solution was diluted with water (30 mL) and extracted with ethyl acetate (3 × 200 mL). The organic components were combined, dried (sodium sulfate), filtered, and concentrated *in vacuo*. Purification using a short pad of neutral alumina, eluting with ethyl acetate in petroleum ether (gradient elution 10 → 100% ethyl acetate) yielded *ethyl 2-nitro-1-{2'-oxo-2'-[(2",2",3",3",3"-pentafluoropropyl)amino]ethyl}-1H-imidazole-5-carboxylate* (**13**, 134 mg, 0.359 mmol, 67%) as a colorless solid. R_f 0.8 (100% ethyl acetate); m.p. 125–128 °C (from ethyl acetate); $\tilde{\nu}_{max}$ (solid)/cm^{-1} 3323 (C-H, w), 2363 (C-H, w), 1723 (C=O, s), 1721 (C=O, s), 1528 (m), 1341 (m), 1287 (s), 1265 (s), 1195 (s); ^{1}H NMR (400 MHz, MeOD) δ 7.80 (1H, s, H-4), 5.70 (2H, s, H-1'), 4.38 (2H, q, *J* 7.1, H-2'''), 4.03 (2H, t, $J_{H\text{-}F}$ 14.8, H-1''), 1.38 (3H, t, *J* 7.1, H-3'''); ^{13}C (151 MHz, MeOD) δ 168.8 (C-2'), 160.4 (C-1'''), 148.8 (C-2), 134.8 (C-4), 127.7 (C-5), 120.2 (qt, $J_{C\text{-}F}$ 285.2, 35.3, C-3''), 114.4 (tq, *J* 253.7, 37.2, C-2''), 63.0 (C-2'''), 50.6 (C-1'), 39.7 (t, $J_{C\text{-}F}$ 25.2, C-1''), 14.4 (C-3'''); ^{19}F NMR (565 MHz, MeOD) δ –85.82 (3F, s, CF_3), –122.86 (2F, t, $J_{F\text{-}H}$ 14.8, CF_2); HRMS *m/z* (ESI$^+$) $C_{11}H_{12}F_5N_4O_5^+$ requires 375.0722, found 375.0723; LRMS *m/z* (ESI$^+$) 375 ([M+H]$^+$, 28%); HPLC retention time 6.8 min, 98%.

*Not visible by ^{13}C NMR – detected using HMBC.

2'-[5-(Hydroxymethyl)-2-nitro-1*H*-imidazol-1-yl]-*N*-(2'',2'',3'',3'',3''-pentafluoroprop yl)acetamide (14)

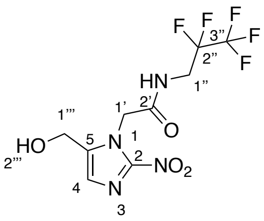

To a solution of **13** (134 mg, 0.359 mmol, 1.0 eq.) in anhydrous tetrahydrofuran (2.5 mL) and anhydrous methanol (0.5 mL), a solution of sodium borohydride (40.8 mg, 1.08 mmol, 3 eq.) in anhydrous ethanol (1 mL) was added dropwise, at 0 ºC, under argon with stirring. After 30 min TLC analysis was performed and the reaction was seen to be incomplete, so further sodium borohydride (17.2 mg, 0.457 mmol, 1.5 eq.) in ethanol (0.6 mL) was added, and the solution was stirred for a further 4 h, at 0 ºC. Methanol (1.5 mL) was then added, and the solution was stirred for 30 min before acidification to pH 5 using an aqueous solution of hydrochloric acid (2 M, 2 mL). The solution was then concentrated *in vacuo*. The resulting residue was diluted with water (20 mL) and extracted with ethyl acetate (5 × 50 mL). The organic fractions were combined, dried (sodium sulfate), filtered, and concentrated *in vacuo.* Purification using silica gel chromatography, eluting with EtOAc yielded *2'-[5-(hydroxymethyl)-2-nitro-1H-imidazol-1-yl]-N-(2'',2'',3'',3'',3''-pentafluoroprop yl)acetamide* (**14**, 80.5 mg, 0.242 mmol, 67%) as a colorless solid. R_f 0.3 (100% ethyl acetate); m.p. 173–175 °C (from ethyl acetate); $\tilde{\nu}_{max}$ (solid)/cm^{-1} 3309 (br), 2363 (w), 1682 (s), 1540 (s), 1341 (s), 1193 (s), 1155 (s); ^{1}H NMR (400 MHz, MeOD) δ 7.14 (1H, s, H-4), 5.28 (2H, s, H-1'), 4.61 (2H, s, H-1'''), 4.01 (2H, t, $J_{H\text{-}F}$ 15.2, H-1''); ^{13}C (151 MHz, MeOD) δ 168.9 (C-2'), 147.1 (C-2), 139.4 (C-5), 127.5 (C-4), 120.2 (qt, $J_{C\text{-}F}$ 285.5, 35.3, C-3''), 114.5 (t $J_{C\text{-}F}$ 37.1, C-2''), 54.6 (C-1'''), 50.0 (C-1'), 39.7 (t, $J_{C\text{-}F}$ 24.8, C-1''); ^{19}F NMR (565 MHz, MeOD) δ –85.84 (3F, s, CF_3), –122.87 (2F, t, $J_{F\text{-}H}$ 15.2, CF_2); HRMS *m/z* (ESI$^+$) $C_9H_{10}F_5N_4O_4^+$ requires 333.0617, found 332.8938; LRMS *m/z* (ESI$^+$) 333 ([M+H]+, 22%), 687 ([2M+Na]$^+$, 100%); HPLC retention time 6.8 min, 96%.

2-(5-((4-Formylphenoxy)methyl)-2-nitro-1*H*-imidazol-1-yl)-*N*-(2,2,3,3,3-pentafluoropropyl)acetamide (16)

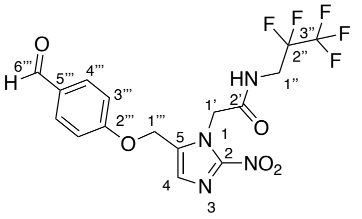

To a solution of **14** (80.5 mg, 0.242 mmol, 1.0 eq.), 4-hydroxybenzaldehyde **15** (74.0 mg, 0.606 mmol, 2.5 eq.) and triphenylphosphine (159 mg, 0.606 mmol, 2.5 eq.) in anhydrous tetrahydrofuran (6.0 mL), was added diisopropyl azodicarboxylate (143 µL, 0.727 mmol, 3.0 eq.) dropwise, at rt. The resulting solution was stirred at rt for 6 h, then concentrated *in vacuo.* The crude residue was dissolved in ethyl acetate (25 mL), washed with a saturated aqueous solution of sodium hydrogen carbonate (3 × 25 mL), water (3 × 25 mL), dried (sodium sulfate), filtered, and concentrated *in vacuo.*. Purification using silica gel column chromatography, eluting with ethyl acetate in petroleum ether (gradient elution 30 → 100% ethyl acetate), followed by precipitation [the residue was dissolved in chloroform (5 mL) and precipitate by addition of petroleum ether (100 mL)] to yield *2-(5-((4-formylphenoxy)methyl)-2-nitro-1H-imidazol-1-yl)-N-(2,2,3,3,3-pentafluoropropyl)acetamide* **16** (60.2 mg, 0.156 mmol, 64%) as a colorless solid. R_f 0.29 (3% methanol in dichloromethane); m.p. 145–149 °C (from acetonitrile); $\tilde{\nu}_{max}$ (solid)/cm^{-1} 1702, 1679, 1509, 1237, 1195; ^{1}H NMR (400 MHz, MeOD) δ 9.86 (1H, s, CHO), 7.90 (2H, d, *J* 8.8, H-4'''), 7.37 (1H, s, H-4), 7.18 (2H, d, *J* 8.8, H-3'''), 5.33 (2H, s, H-1'''), 5.27 (2H, s, H-1'), 2.63 (2H, t, $J_{H\text{-}F}$ 15.3, H-1''); ^{13}C NMR (151 MHz, MeOD) δ 192.8 (C-6'''), 168.5 (C-2'), 164.1 (C-2'''), 147.6 (C-2), 134.5 (C-5), 133.0 (C-5'''), 132.3 (C-4'''), 129.1 (C-4), 120.5 (qt, $J_{C\text{-}F}$ 285.5, 34.7,C-3''), 116.4 (C-3'''), 114.6 (t, $J_{C\text{-}F}$ 45.3, C-2''), 60.7 (C-1'''), 50.2 (C-1'), 39.7 (t, $J_{C\text{-}F}$ 24.7, C-1''); ^{19}F NMR (565 MHz, D_6-DMSO) δ –85.1 (3F, s, CF_3), –122.1 (2F, s, CF_2); LRMS *m/z* (ESI$^+$) 437 ([M+H]$^+$, 100%); HRMS (ESI$^+$) $C_{16}H_{14}F_5N_4O_5^+$ requires 437.0879, found 437.0868; HPLC retention time 7.930 min, 100%. N.B. The ^{13}C spectrum indicates hemi-acetal formation in MeOH. Acetal peaks are not reported.

(*E*)-2-(5-((4-(2-(4-(Dicyanomethylene)-4*H*-chromen-2-yl)vinyl)phenoxy)methyl)-2-nitro-1*H*-imidazol-1-yl)-*N*-(2,2,3,3,3-pentafluoropropyl)acetamide (4)

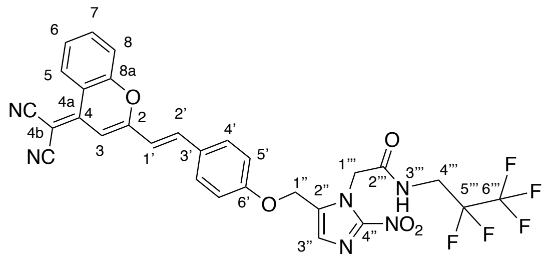

To a suspension of **16** (33.5 mg, 76.8 μmol, 1.0 eq.) and 2-(2-methyl-4*H*-chromen-4-ylidene) malonitrile (17.6 mg, 84.5 μmol, 1.1 eq.) in anhydrous ethanol (3 mL) was added piperidine (20.5 μL, 207 μmol, 2.7 eq.). The suspension was heated under reflux for 3 h, then cooled to RT and concentrated *in vacuo*. Purification using silica gel chromatography, eluting with ethyl acetate in petroleum ether (gradient elution 0 → 100% ethyl acetate), followed by a series of precipitation. The first was from dichloromethane by adding petroleum ether, then precipitation from ethyl acetate by adding hexane yielded *(E)-2-(5-((4-(2-(4-(dicyanomethylene)-4H-chromen-2-yl)vinyl)phenoxy)methyl)-2-nitro-1H-imidazol-1-yl)-N-(2,2,3,3,3-pentafluoropropyl)acetamide* (**4**, 20.5 mg, 32.7 μmol, 42%) as an orange solid. R_f 0.42 (2% methanol in dichloromethane); m.p. 180–185 °C (from ethanol); $\tilde{\nu}_{max}$ (solid)/cm^{-1} 2361 (w), 2211 (w) , 1596 (s), 1557 (s), 1340 (m); ^{1}H NMR (400 MHz, D$_6$-acetone) δ 8.85 (1H, d, *J* 8.5, H-5), 8.24 (1H, t, *J* 6.2, H-3'''), 7.90 (1H, dd, *J* 8.3, 7.2, H-7), 7.81 (1H, d, *J* 16.1, H-2'), 7.81–7.72 (3H, m, H-8, H-4'), 7.59 (1H, dd, *J* 8.5, 7.2, H-6), 7.37 (1H, s, H-3''), 7.26 (1H, d, *J* 16.1, H-1'), 7.13 (2H, d, *J* 8.5, H-5'), 6.91 (1H, s, H-3), 5.42 (2H, s, H-1''), 5.36 (2H, s, H-1'''), 4.14 (2H, dt, *J* 15.5, 6.2, H-4'''); ^{13}C NMR (151 MHz, D$_6$-acetone) δ 167.4 (C-2'''), 160.6 (C-6'), 159.4 (C-2), 153.8 (C-4), 153.4 (C-8a), 147.6 (C-4''), 139.2 (C-4'), 136.0 (C-7), 134.1 (C-2''), 130.8 (C-8), 129.9 (C-3'), 129.4 (C-3''), 126.8 (C-6), 126.1 (C-5), 119.8 (C-2'), 119.9 (qt, J_{C-F} 285.5, 34.7, C-6'''), 118.5 (C-1'), 118.4 (C-4a), 117.7 (C-4bCN), 116.43 (C-5'), 116.38 (C^{4b}-CN), 114.3 (t, J_{C-F} 37.8, C-5'''), 107.1 (C-3), 62.2 (C-4b), 60.4 (C-1''), 50.1 (C-1'''), 39.4 (t, J_{C-F} 24.2, C-4'''); ^{19}F NMR (565 MHz, D$_6$-acetone) δ –85.1 (3F, s, CF_3), –122.1 (2F, s, CF_2); HRMS (ESI$^+$) $C_{29}H_{20}F_5N_6O_5^+$ requires 627.1410, found 627.1407; LRMS (ESI$^+$) *m/z* 627 ([M+H]$^+$, 100%); HPLC retention time 10.262 min, 97%.

(*E*)-*tert*-Butyl-3-(2-((tert-butoxycarbonyl)(4-(3-(hydroxyamino)-3-oxoprop-1-en-1-yl)benzyl)amino)ethyl)-2-methyl-1*H*-indole-1-carboxylate (18)

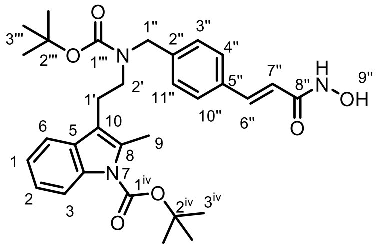

(*E*)-3-(4-[(*tert*-Butyloxycarbonyl-[2-(1-[*tert*-butyloxycarbonyl]-2-methyl-1*H*-indol-3-yl)ethyl]amino)methyl]phenyl)prop-2-enoic acid was synthesized as previously described.[5] To a solution of the compound (133 mg, 0.249 mmol, 1.0 eq.) in dry tetrahydrofuran (1.0 mL), was added 1,1'-carbonyl diimidazole (63.5 mg, 0.392 mmol, 1.6 eq.) and the solution stirred at rt for 4 h. Hydroxylamine hydrochloride (36.5 mg, 0.525 mmol, 2.1 eq.) was added, and the was solution stirred for a further 19 h. After this time the reaction was quenched with an aqueous solution of hydrochloric acid (1 M, 1 mL) and extracted with ethyl acetate (2 × 20 mL). The combined organic components were washed with brine (50 mL), dried (sodium sulfate), filtered, and concentrated *in vacuo*. Purification using silica gel chromatography, eluting with ethanol in chloroform (gradient elution 0 → 3% ethanol) yielded (*E*)-*tert*-butyl-3-(2-((tert-butoxycarbonyl)(4-(3-(hydroxyamino)-3-oxoprop-1-en-1-yl)benzyl)amino)ethyl)-2-methyl-1*H*-indole-1-carboxylate (**18**, 58 mg, 0.106 mmol, 42%) as a pale-pink solid. R_f 0.28 (10% ethanol in chloroform); m.p. 99–102 °C (chloroform) [lit. 104–106 °C (from ethanol)];[6] ^{1}H NMR (400 MHz, D_6-DMSO) δ 10.75 (1H, s, NH), 9.04 (1H, s, H-9''), 8.01 (1H, d, *J* 8.1, H-3), 7.55–7.37 (4H, m, H-3'', H-4'', H-11'', H-10''), 7.29–7.13 (4H, m, H-6, H-1, H-2, H-6''), 6.43 (1H, d, *J* 15.9, H-7''), 4.40 (2H, s, H-1''), 3.27 (2H, t, *J* 7.9, H-2') 2.81 (2H, t, *J* 7.1, H-1'), 2.43 (3H, s, H-9), 1.61 (9H, s, H-3^{v}), 1.29 (9H, s, H-3^{vi}); LRMS (ESI$^+$) 549 ([M+H]$^+$, 100%). The spectroscopic data are in good agreement with the literature values.[5]

***tert*-Butyl (*E*)-3-(2-((*tert*-butoxycarbonyl)(4-(3-(((2-nitro-1-(2-oxo-2-((2,2,3,3,3-pentafluoropropyl)amino)ethyl)-1*H*-imidazol-5-yl)methoxy)amino)-3-oxoprop-1-en-1-yl)benzyl)amino)ethyl)-2-methyl-1*H*-indole-1-carboxylate (23)**

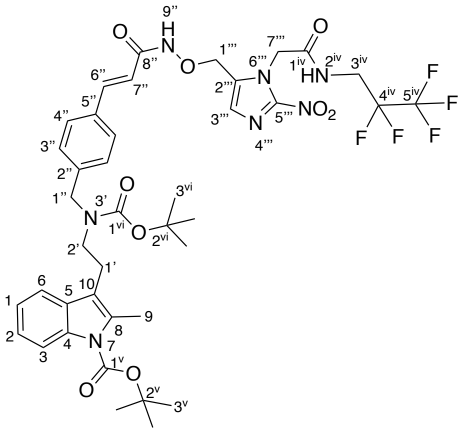

To a solution of **18** (186 mg, 0.339 mmol, 1.0 eq.), **7** (123 mg, 0.381 mmol, 1.1 eq.), and triphenylphosphine (222 mg, 0.847 mmol, 2.5 eq.) in anhydrous tetrahydrofuran (4.2 mL), diisopropyl azodicarboxylate (201 µL, 1.01 mmol, 3.0 eq.) was added dropwise, at rt. The solution was stirred for 2.5 h, then diluted with H_2O (25 mL) and extracted with ethyl acetate (3 × 25 mL). The organic components were combined, dried (sodium sulfate), filtered, and concentrated *in vacuo*. Purification using silica gel chromatography, eluting with ethyl acetate in petroleum ether (gradient elution 0 → 60%), followed by reverse phase chromatography, eluting with acetonitrile in water (gradient elution 0 → 70%) yielded **23** (51 mg, 0.0591 mmol, 17%) as a colorless solid. R_f 0.34 (4% methanol in dichloromethane); m.p. 81–85 °C (from acetonitrile); $\tilde{\nu}_{max}$ (solid)/cm^{-1} 1723, 1685, 1665, 1541; ^{1}H NMR (400 MHz, D_6-DMSO) δ 10.76 (1H, s, H-9''), 8.96 (1H, t, *J* 5.4, H-2^{iv}), 8.00 (1H, d, *J* 8.1, H-6), 7.47 (2H, d, *J* 8.0, H-4''), 7.42 (1H, d, *J* 8.0, H-3), 7.34 (1H, s, H-3'''), 7.22–7.14 (2H, m, H-1, H-2), 7.20 (2H, d, *J* 7.9, H-3''), 6.90 (1H, d, *J* 16.1, H-6''), 6.53 (1H, d, *J* 16.1, H-7''), 5.34 (2H, s, H-1'''), 5.28 (2H, s, H-7'''), 4.39 (2H, s, H-1''), 3.95 (2H, td, *J* 15.0, 4.4, H-3^{iv}), 3.29 (2H, t, *J* 7.1, H-2'), 2.82 (2H, t, *J* 7.3, H-1'), 2.45 (3H, s, H-9), 1.63 (9H, s, H-3^{v}), 1.31 (9H, s, H-3^{vi}); ^{13}C NMR (151 MHz, D_6-DMSO), δ 166.3 (C-1^{iv}), 153.3 (C-8''), 149.7 (C-1^{v}), 145.8 (C-5'''), 138.9 (C-2''), 135.0 (C-4), 134.3 (C-5''), 133.8 (C-2'''), 133.0 (C-8), 132.7 (C-6''), 129.2 (C-5), 128.3 (C-3'''), 127.5 (C-3''), 126.8 (C-4''), 123.0 (C-2), 122.0 (C-1), 118.9 (C-7''), 117.3 (C-3), 115.0 (C-10), 114.6 (C-6), 112.4* (C-4^{iv}), 83.3 (C-2^{v}), 78.6 (C-2^{vi}), 60.8 (C-1'''), 48.7 (C-7'''), 48.6* (C-1''), 45.6* (C-2'), 37.9 (t, *J* 24.9, C-3^{iv}),

27.6 (C-3[v]), 27.5 (C-3[vi]), 22.1 (C-1'), 13.0 (C-9); F^{19} NMR (565 MHz, D_6-DMSO) δ −119.92 (2F, s, CF_2), -83.46 (3F, s, CF_3); HRMS (ESI^+) $C_{40}H_{47}F_5N_7O_9^+$ requires 864.3355, 864.3344 found; LRMS (ESI^+) 863 ($[M+H]^+$, 100%); HPLC retention time 11.744 min, 96%. Due to poor solubility C-1'' was not visible by ^{13}C NMR – detected by HSQC conducted at RT. C-2' was not visible by ^{13}C NMR – detected by HMBC (the ^1H resonance at 2.81 ppm correlates with the ^{13}C resonance at 46.0 ppm) or HSQC conducted at RT. C-2[vi] was deduced by a process of elimination. C-4[iv] deduced by HMBC (3.96 ppm proton correlates with 112.4 ppm carbon). C-5[iv] was not observed.

(*E*)-3-(4-(((2-(2-methyl-1*H*-indol-3-yl)ethyl)amino)methyl)phenyl)-*N*-((2-nitro-1-(2-oxo-2-((2,2,3,3,3-pentafluoropropyl)amino)ethyl)-1*H*-imidazol-5-yl)methoxy)acrylamide (2)

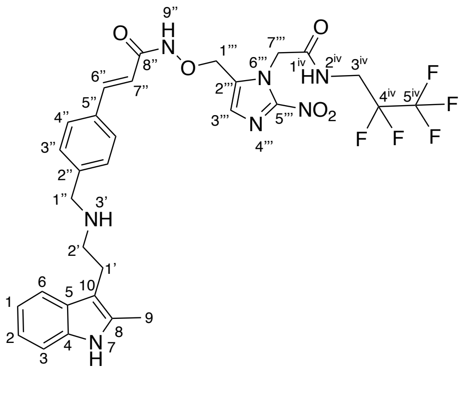

To a solution of *tert*-butyl (*E*)-3-(2-((*tert*-butoxycarbonyl)(4-(3-(((2-nitro-1-(2-oxo-2-((2,2,3,3,3-pentafluoropropyl)amino)ethyl)-1*H*-imidazol-5-yl)methoxy)amino)-3-oxoprop-1-en-1-yl)benzyl)amino)ethyl)-2-methyl-1*H*-indole-1-carboxylate (22 mg, 25.5 μmol, 1.0 eq.) and triisopropylsilane (1.04 μL, 5.09 μmol, 0.2 eq.) in anhydrous dichloromethane (2.55 mL), trifluoroacetic acid (0.51 mL, 20% *v/v*) was added dropwise, at RT. The resulting solution was stirred for 45 min, then cooled to 0 °C, and adjusted to a neutral pH with anhydrous triethylamine (0.9 mL). The crude mixture was concentrated under a stream of nitrogen, then diluted with MeCN (1.5 mL), and purified using semi-preparative HPLC (see above for method). Pure fractions were immediately filtered through Celite, then lyophilized, yielding *(E)-3-(4-(((2-(2-methyl-1H-indol-3-yl)ethyl)amino)methyl)phenyl)-N-((2-nitro-1-(2-oxo-2-((2,2,3,3,3-pentafluoropropyl)amino)ethyl)-1H-imidazol-5-yl)methoxy)acrylamide* (**2**, 7.8 mg, 11.8 μmol, 46%) as a pale-yellow solid. R_f 0.47 (1.5:3.5:95 water: isopropanol: ethyl acetate); m.p. 138–142 °C (from acetonitrile); $\tilde{\nu}_{max}$ (solid)/cm^{-1}

3358 (w), 3194 (w), 2957 (s), 2923 (s), 2852 (m), 1660 (m), 1633 (m), 1560 (m), 1093 (m); ^{1}H NMR (400 MHz, 70:30 CD_3CN:D_2O +0.1% formic acid) δ 7.51 (2H, d, *J* 8.0, H-4''), 7.39 (2H, d, *J* 8.0, H-3''), 7.38 (1H, d, *J* 7.0, H-6), 7.28 (1H, s, H-3'''), 7.28 (1H, d, *J* 7.0, H-3), 7.04 (1H, ddd, *J* 8.2, 7.0, 1.2, H-2), 6.97 (1H, d, *J* 16.1, H-6''), 6.97 (1H, ddd, *J* 8.2, 7.0, 1.2, H-1) 6.56 (1H, d, *J* 16.1, H-7''), 5.32 (2H, s, H-7'''), 5.30 (2H, s, H-1'''), 4.13 (2H, s, H-1''), 3.91 (2H, t, *J* 15.2, H-3^{iv}), 3.12-3.08 (2H, m, H-2'), 3.05-3.03 (2H, m, H-1'), 2.31 (3H, s, H-9); ^{13}C NMR (151 MHz, 70:30 CD_3CN:D_2O +0.1% formic acid) δ 168.0 (C-1^{iv}), 155.7 (C-8''), 137.8 (C-5''), 136.5 (C-4), 134.9 (C-2'''), 134.5 (C-6''), 134.4 (C-8), 132.2 (C-2''), 131.5 (C-3''), 129.9 (C-3'''), 128.9 (C-5), 128.7 (C-4''), 121.8 (C-2), 120.9 (C-7''), 119.9 (C-1), 118.2 (C-6), 111.6 (C-3), 105.7 (C-10), 62.3 (C-1'''), 51.3 (C-1''), 50.3 (C-7'''), 48.1 (C-2'), 39.3 (d *J* 25.7 C-3^{iv}), 21.6 (C-1'), 11.4 (C-9); F^{19} NMR (565 MHz, D_6-DMSO) δ –122.0 (2F, s, CF_2), –85.0 (3F, s, CF_3); LCMS *m/z* (ESI$^+$) [Found: 664.200, $C_{30}H_{31}F_5N_7O_5$ requires $[M+H]^+$ 664.230); LRMS *m/z* (ESI$^+$) 664 ([M+H$^+$]), 100%; HPLC retention time 7.115 min, 99.7%. * Due to poor solubility C-4^{iv} and C-5^{iv} were not observed.

^{1}H NMR (400 MHz, D_6-DMSO) δ 10.99 (1H, s, H-7), 10.69 (1H, s, H-9''), 9.14 (1H, t, *J* 6.3, H-2^{iv}), 8.28 (1H, s, H-3'), 7.48 (2H, d, *J* 8.0, H-4''), 7.37 (1H, s, H-3'''), 7.36 (1H, d, *J* 8.1, H-6), 7.32 (2H, d, *J* 8.0, H-3''), 7.20 (1H, d, *J* 8.1, H-3), 6.95 (1H, ddd, *J* 8.1, 7.1, 1.2, H-2), 6.88 (1H, ddd, *J* 8.1, 7.1, 1.2, H-1), 6.87 (1H, d, *J* 16.1, H-6''), 6.57 (1H, d, *J* 16.1, H-7''), 5.33 (2H, s, H-7'''), 5.29 (2H, s, H-1'''), 3.98 (2H, dt, *J* 16.0, 6.3, H-3^{iv}), 3.80 (2H, s, H-1''), 2.82-2.78 (2H, m, H-2'), 2.74-2.70 (2H, m, H-1'), 2.29 (3H, s, H-9).

4. NMR spectra

2-(5-((4-Formylphenoxy)methyl)-2-nitro-1*H*-imidazol-1-yl)-*N*-(2,2,3,3,3-pentafluoropropyl)acetamide (16) ^{1}H NMR spectrum

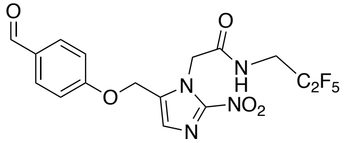

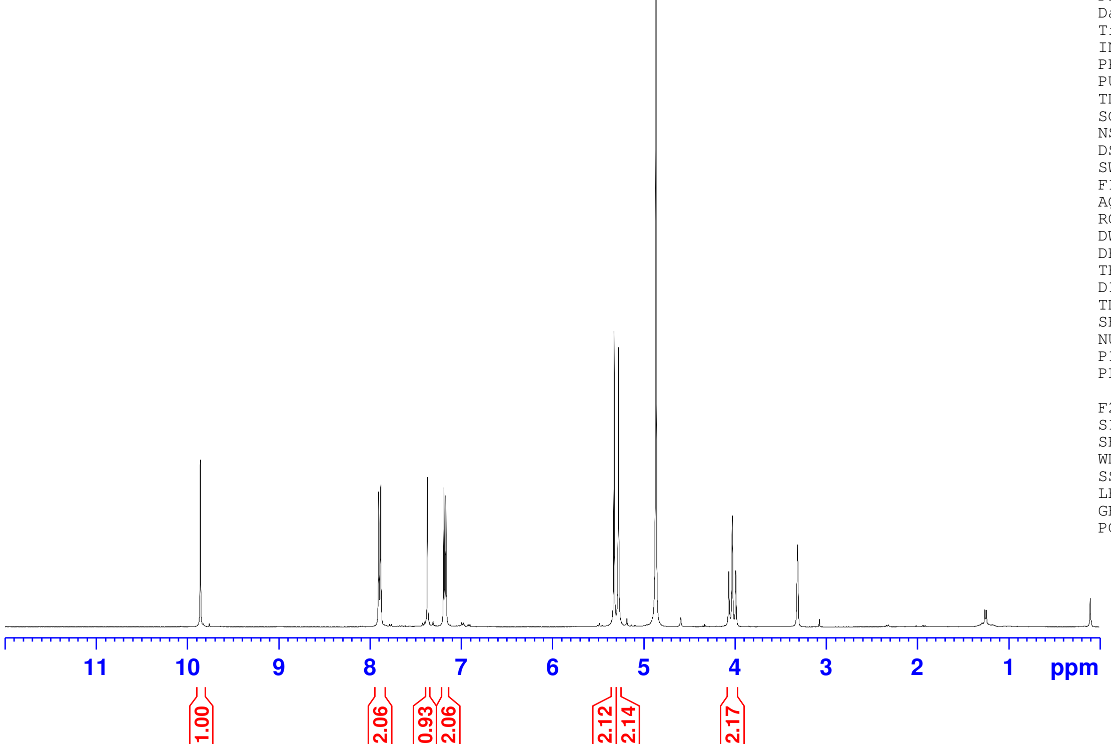


```
Current Data Parameters
NAME      B49 Open access
EXPNO                   1
PROCNO                  1

F2 - Acquisition Parameters
Date_            20211129
Time                 9.41 h
INSTRUM            avh400
PROBHD   Z108618_0873 (
PULPROG              zg60
TD                  65536
SOLVENT              MeOD
NS                     16
DS                      2
SWH              8012.820 Hz
FIDRES           0.244532 Hz
AQ              4.0894465 sec
RG                  88.17
DW                 62.400 usec
DE                   6.50 usec
TE                  -32.4 K
D1             1.00000000 sec
TD0                     1
SFO1          400.1324008 MHz
NUC1                   1H
P1                  14.00 usec
PLW1          14.36999989 W

F2 - Processing parameters
SI                  32768
SF            400.1300080 MHz
WDW                    EM
SSB                     0
LB                   0.30 Hz
GB                      0
PC                   1.00
```

2-(5-((4-Formylphenoxy)methyl)-2-nitro-1*H*-imidazol-1-yl)-*N*-(2,2,3,3,3-pentafluoropropyl)acetamide (16) ^{13}C NMR spectrum

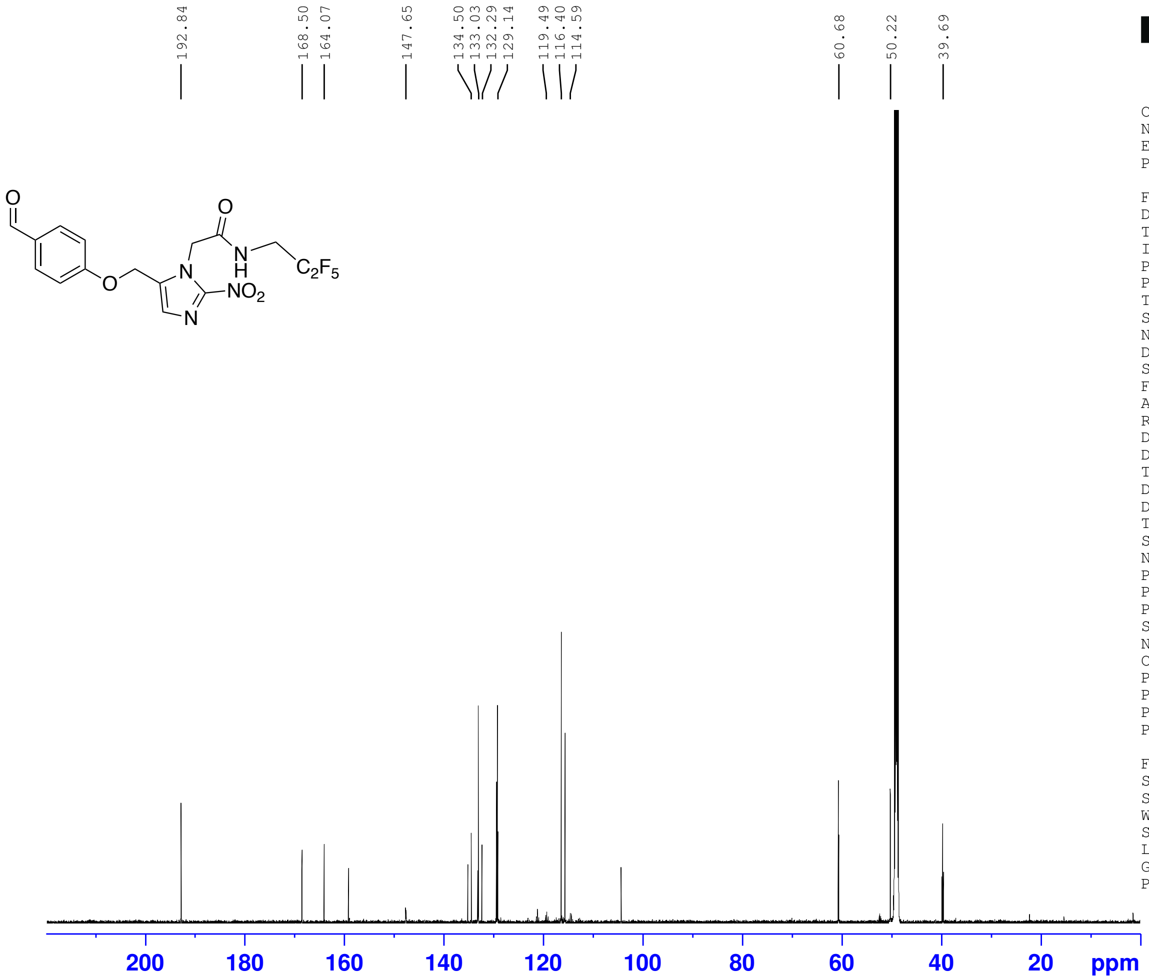


```
Current Data Parameters
NAME          B49 Service
EXPNO                   5
PROCNO                  1

F2 - Acquisition Parameters
Date_            20211201
Time                20.37 h
INSTRUM            Avance
PROBHD   Z159656_0020 (
PULPROG           zgpg30
TD                 65536
SOLVENT             MeOD
NS                   512
DS                     4
SWH            35714.285 Hz
FIDRES          1.089913 Hz
AQ             0.9175040 sec
RG                   101
DW                14.000 usec
DE                 18.00 usec
TE                 298.0 K
D1            2.00000000 sec
D11           0.03000000 sec
TD0                    1
SFO1         150.9908267 MHz
NUC1                 13C
P0                  3.33 usec
P1                 10.00 usec
PLW1         41.91400146 W
SFO2         600.4224017 MHz
NUC2                  1H
CPDPRG[2         waltz16
PCPD2              80.00 usec
PLW2         13.51200008 W
PLW12         0.30124050 W
PLW13         0.15098180 W

F2 - Processing parameters
SI                 65536
SF           150.9755187 MHz
WDW                   EM
SSB                    0
LB                  1.00 Hz
GB                     0
PC                  1.40
```

2-(5-((4-Formylphenoxy)methyl)-2-nitro-1*H*-imidazol-1-yl)-*N*-(2,2,3,3,3-pentafluoropropyl)acetamide (16) ^{19}F NMR spectrum

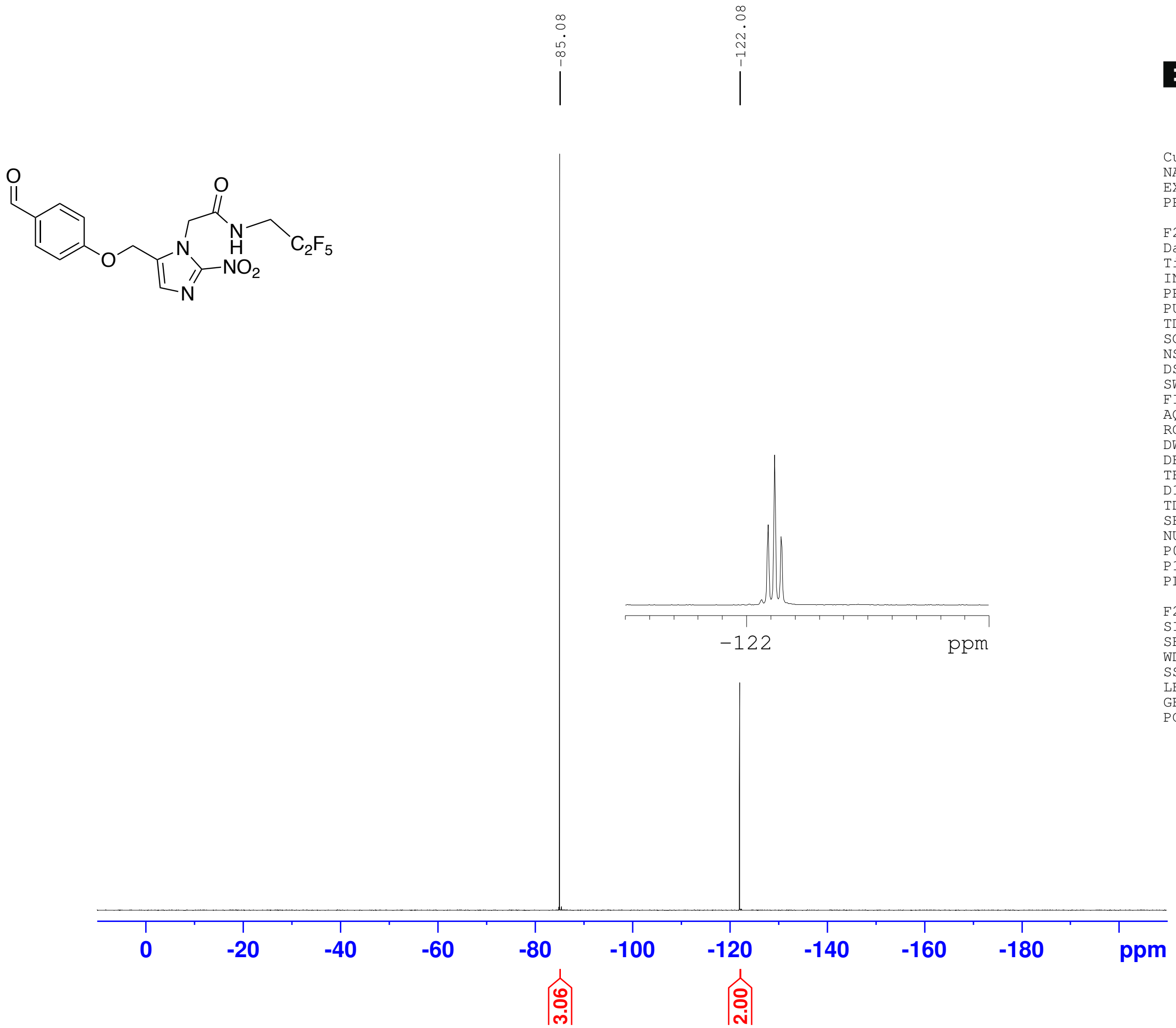


```
Current Data Parameters
NAME      B49 Service in Acetone
EXPNO                    6
PROCNO                   1

F2 - Acquisition Parameters
Date_             20220712
Time                  9.19 h
INSTRUM             Avance
PROBHD    Z159656_0020 (
PULPROG               zg30
TD                  131072
SOLVENT            Acetone
NS                      16
DS                       4
SWH             138888.891 Hz
FIDRES            2.119276 Hz
AQ               0.4718592 sec
RG                 12.3106
DW                   3.600 usec
DE                   18.00 usec
TE                   298.0 K
D1              1.00000000 sec
TD0                      1
SFO1           564.9027649 MHz
NUC1                   19F
P0                    5.00 usec
P1                   15.00 usec
PLW1           11.45499992 W

F2 - Processing parameters
SI                   65536
SF             564.9592608 MHz
WDW                     EM
SSB                      0
LB                    0.30 Hz
GB                       0
PC                    1.00
```

(*E*)-2-(5-((4-(2-(4-(Dicyanomethylene)-4*H*-chromen-2-yl)vinyl)phenoxy)methyl)-2-nitro-1*H*-imidazol-1-yl)-*N*-(2,2,3,3,3-pentafluoropropyl)acetamide (4) ^{1}H NMR spectrum

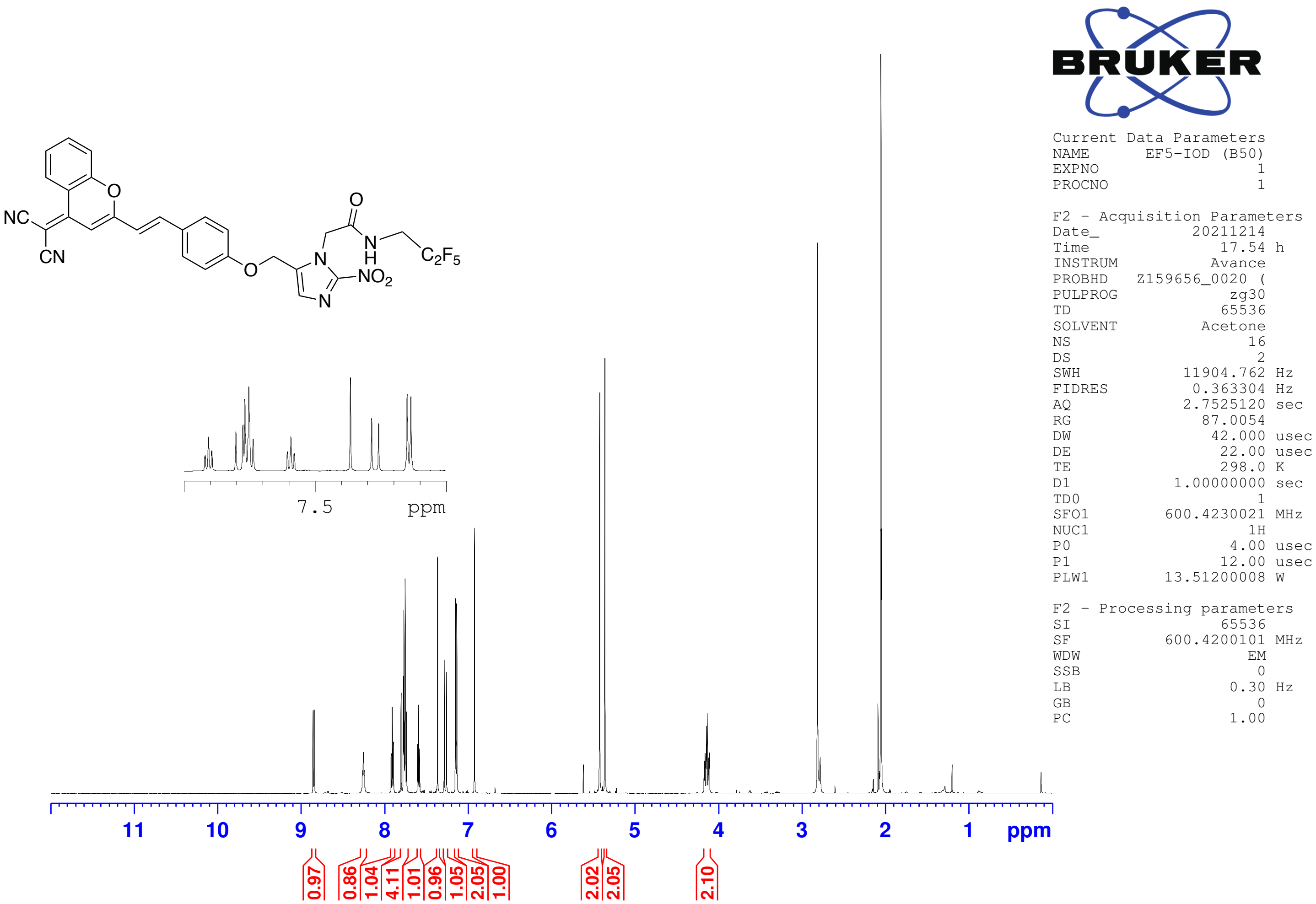

E)-2-(5-((4-(2-(4-(Dicyanomethylene)-4*H*-chromen-2-yl)vinyl)phenoxy)methyl)-2-nitro-1*H*-imidazol-1-yl)-*N*-(2,2,3,3,3-pentafluoropropyl)acetamide (4) ^{13}C NMR spectrum

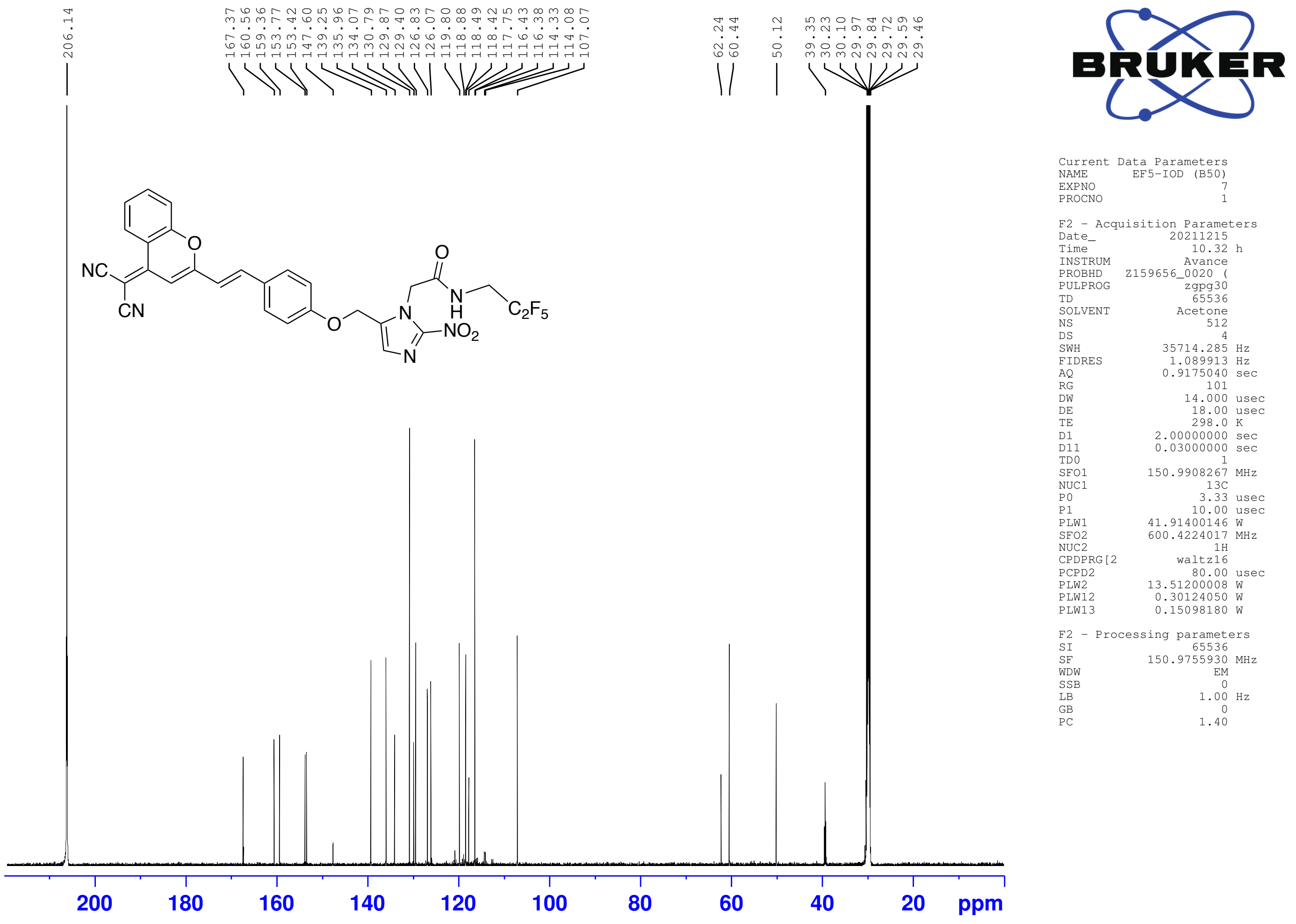

(*E*)-2-(5-((4-(2-(4-(Dicyanomethylene)-4*H*-chromen-2-yl)vinyl)phenoxy)methyl)-2-nitro-1*H*-imidazol-1-yl)-*N*-(2,2,3,3,3-pentafluoropropyl)acetamide (4) ^{19}F NMR spectrum

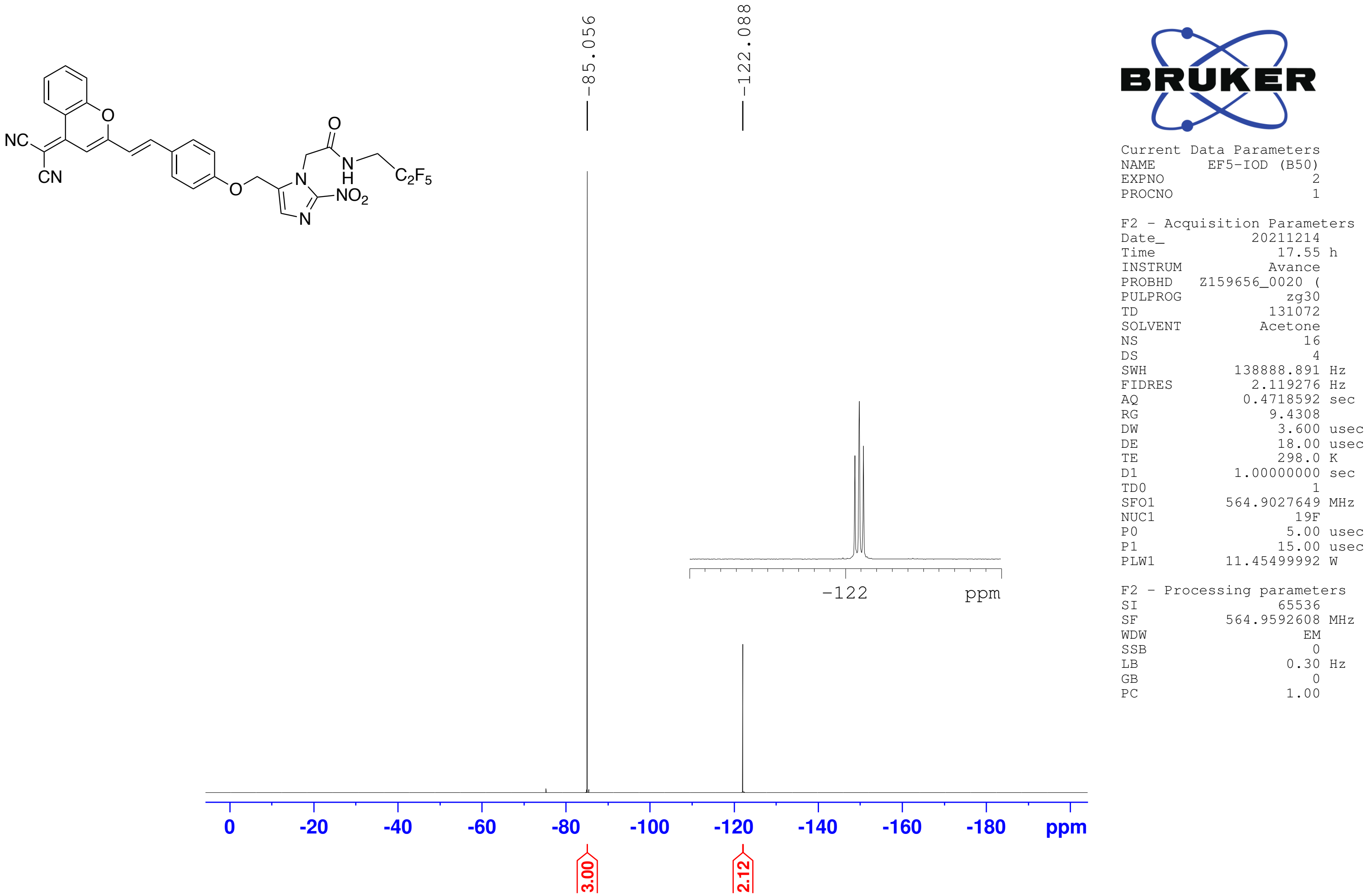

(*E*)-3-(2-((*tert*-Butoxycarbonyl)(4-(3-(((2-nitro-1-(2-oxo-2-((2,2,3,3,3-pentafluoropropyl)amino)ethyl)-1*H*-imidazol-5-yl)methoxy)amino)-3-oxoprop-1-en-1-yl)benzyl)amino)ethyl)-2-methyl-1*H*-indole-1-carboxylate ^{1}H NMR spectrum

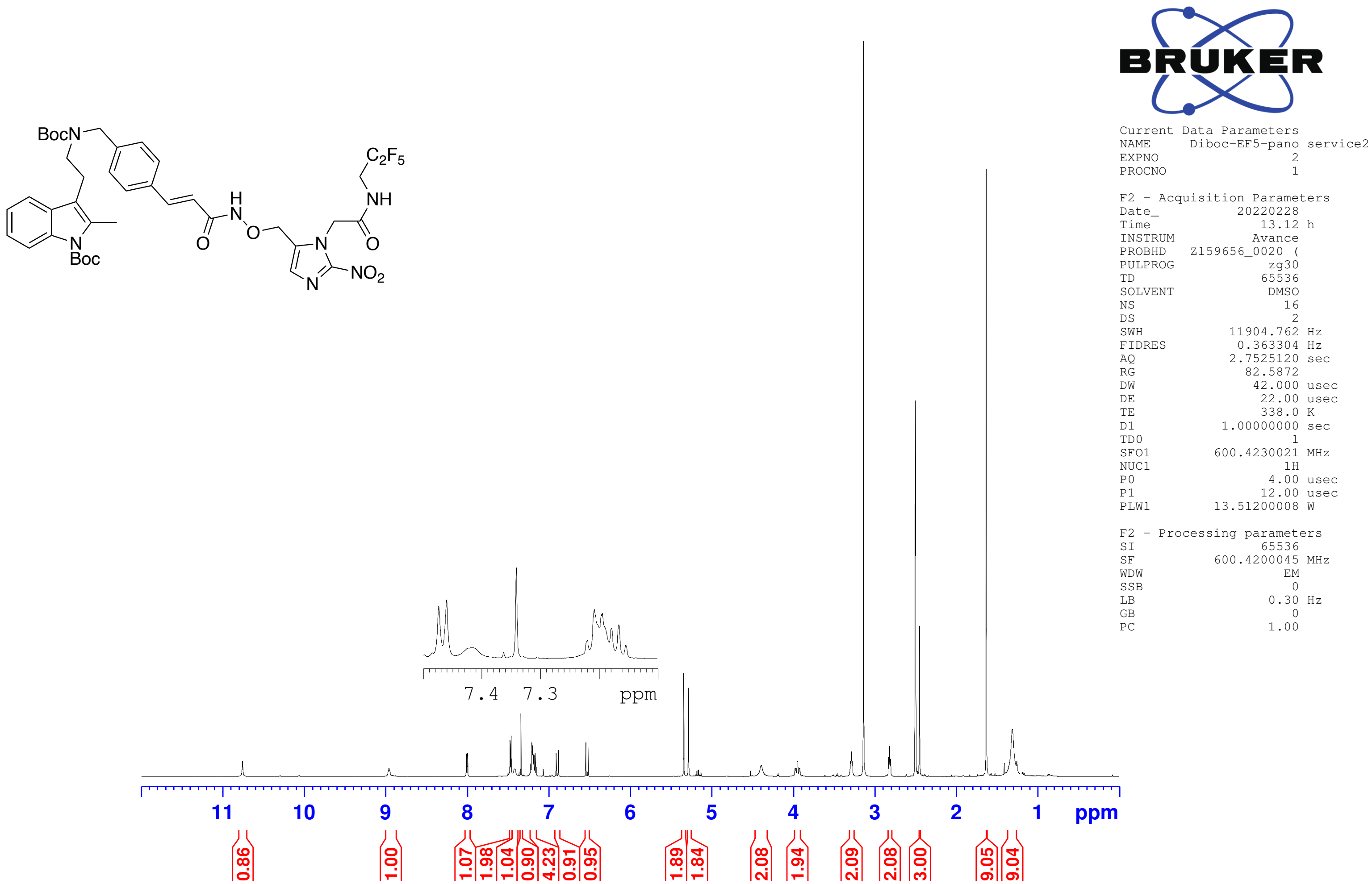

tert-Butyl (_E_)-3-(2-((_tert_-butoxycarbonyl)(4-(3-(((2-nitro-1-(2-oxo-2-((2,2,3,3,3-pentafluoropropyl)amino)ethyl)-1_H_-imidazol-5-yl)methoxy)amino)-3-oxoprop-1-en-1-yl)benzyl)amino)ethyl)-2-methyl-1_H_-indole-1-carboxylate ^{13}C NMR spectrum

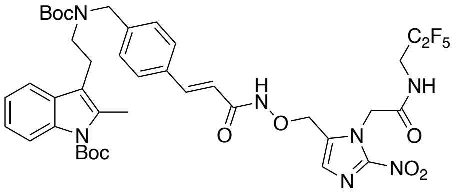

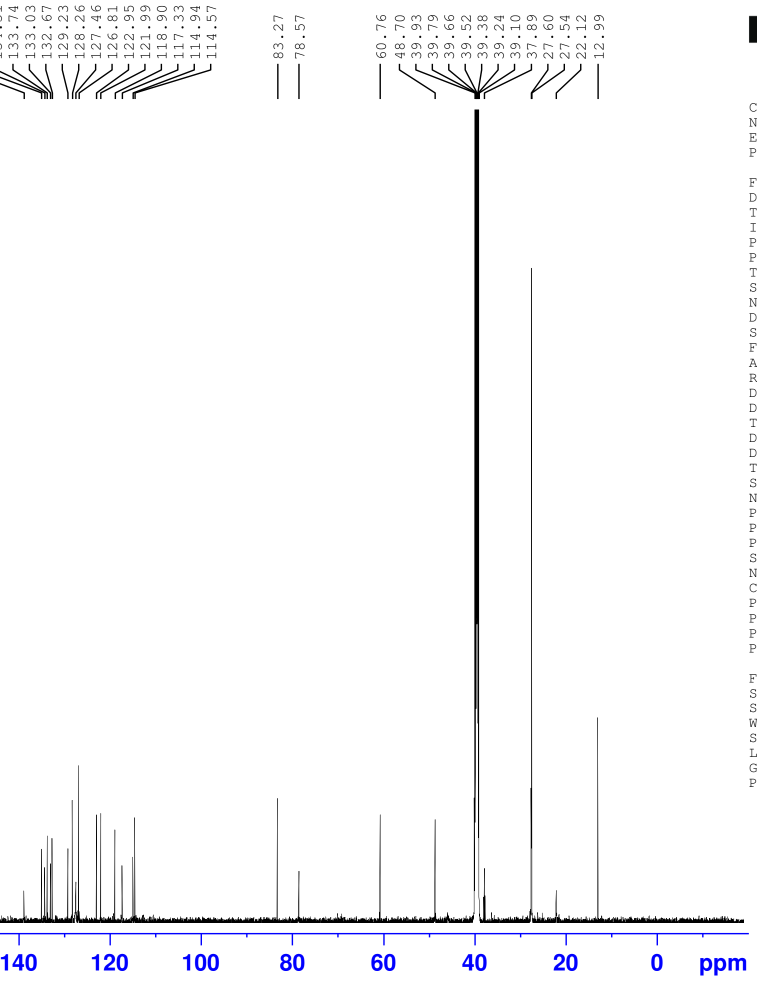

BRUKER

Current Data Parameters
NAME Diboc-EF5-pano service2
EXPNO 3
PROCNO 1

F2 - Acquisition Parameters
Date_ 20220228
Time 14.05 h
INSTRUM Avance
PROBHD Z159656_0020 (
PULPROG zgpg30
TD 65536
SOLVENT DMSO
NS 1024
DS 4
SWH 35714.285 Hz
FIDRES 1.089913 Hz
AQ 0.9175040 sec
RG 101
DW 14.000 usec
DE 18.00 usec
TE 338.0 K
D1 2.00000000 sec
D11 0.03000000 sec
TD0 1
SFO1 150.9908267 MHz
NUC1 13C
P0 3.33 usec
P1 10.00 usec
PLW1 41.91400146 W
SFO2 600.4224017 MHz
NUC2 1H
CPDPRG[2 waltz16
PCPD2 80.00 usec
PLW2 13.51200008 W
PLW12 0.30124050 W
PLW13 0.15098180 W

F2 - Processing parameters
SI 65536
SF 150.9758465 MHz
WDW EM
SSB 0
LB 1.00 Hz
GB 0
PC 1.40

tert-Butyl (_E_)-3-(2-((_tert_-butoxycarbonyl)(4-(3-(((2-nitro-1-(2-oxo-2-((2,2,3,3,3-pentafluoropropyl)amino)ethyl)-1_H_-imidazol-5-yl)methoxy)amino)-3-oxoprop-1-en-1-yl)benzyl)amino)ethyl)-2-methyl-1_H_-indole-1-carboxylate ^{19}F NMR spectrum

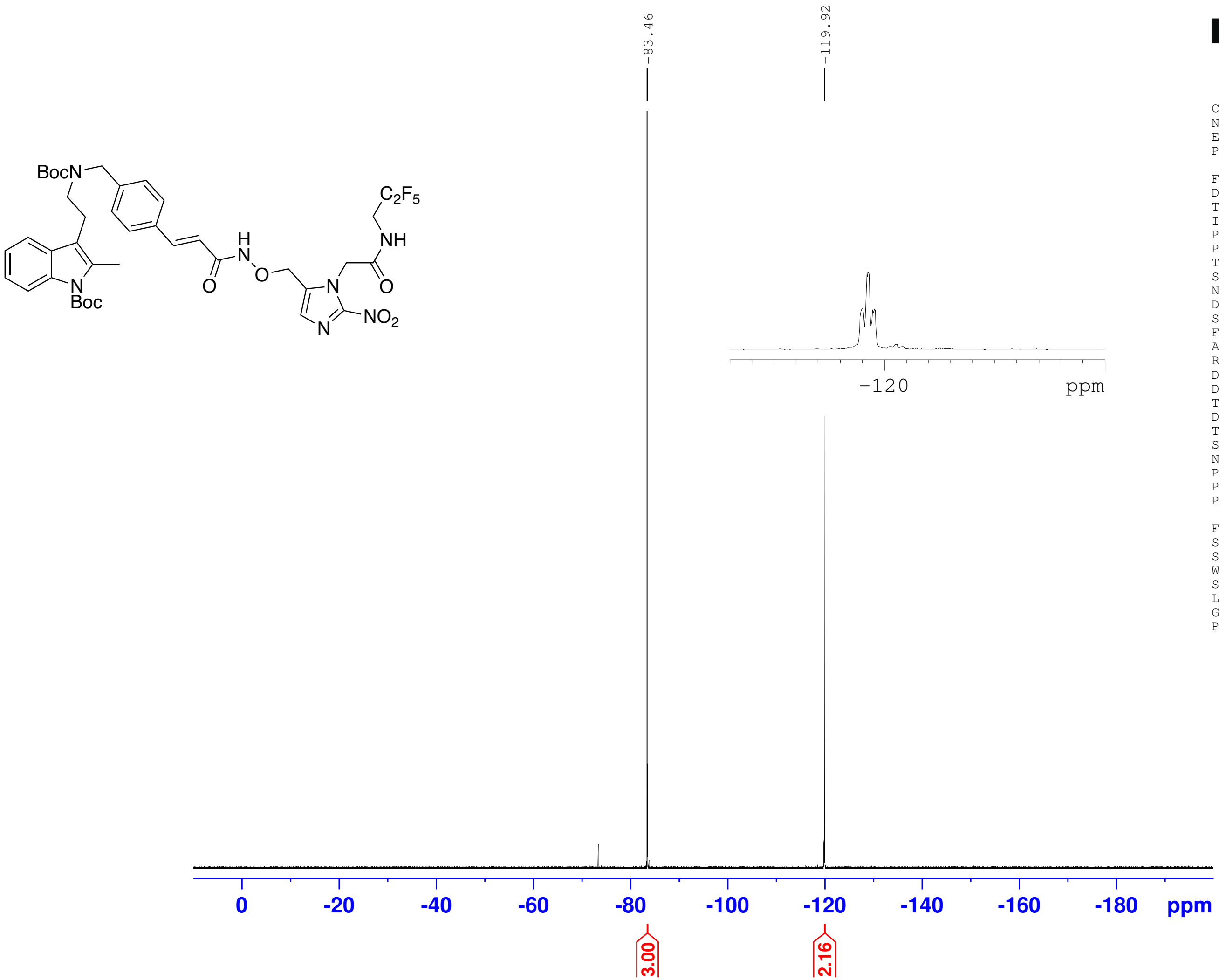


```
Current Data Parameters
NAME     Diboc-EF5-pano service2
EXPNO                 7
PROCNO                1

F2 - Acquisition Parameters
Date_          20220228
Time              14.32 h
INSTRUM          Avance
PROBHD    Z159656_0020 (
PULPROG            zg30
TD               131072
SOLVENT            DMSO
NS                   16
DS                    4
SWH          138888.891 Hz
FIDRES         2.119276 Hz
AQ            0.4718592 sec
RG              9.34099
DW                3.600 usec
DE                18.00 usec
TE                338.0 K
D1           1.00000000 sec
TD0                   1
SFO1        564.9027649 MHz
NUC1                19F
P0                 5.00 usec
P1                15.00 usec
PLW1        11.45499992 W

F2 - Processing parameters
SI                65536
SF          564.9592608 MHz
WDW                  EM
SSB                   0
LB                 0.30 Hz
GB                    0
PC                 1.00
```

(*E*)-3-(4-(((2-(2-Methyl-1*H*-indol-3-yl)ethyl)amino)methyl)phenyl)-*N*-((2-nitro-1-(2-oxo-2-((2,2,3,3,3-pentafluoropropyl)amino)ethyl)-1*H*-imidazol-5-yl)methoxy)acrylamide (2) ^{1}H NMR spectrum

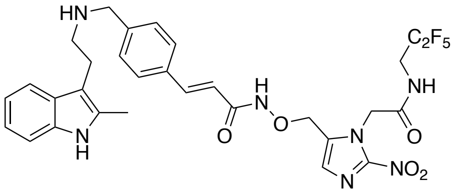

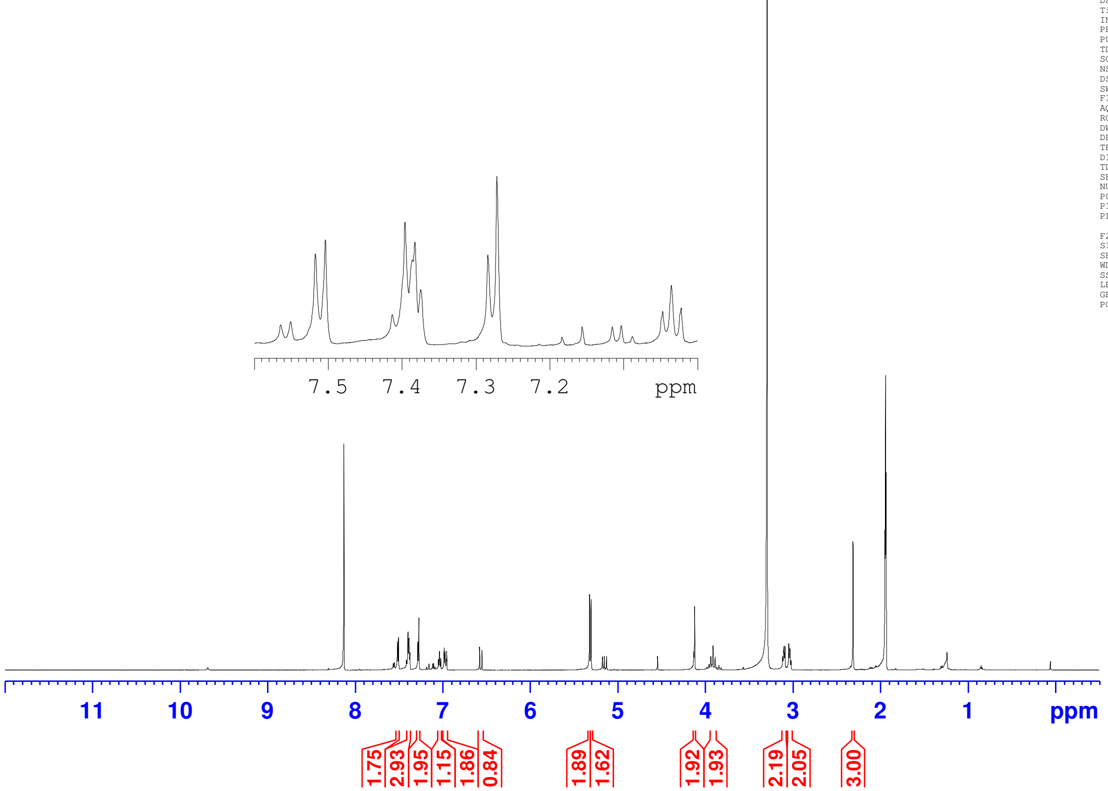


```
Current Data Parameters
NAME      EF5 Pano Service - 7030 ACN-H20 +0.1% FA
EXPNO                    1
PROCNO                   1

F2 - Acquisition Parameters
Date_             20220617
Time                 12.00 h
INSTRUM             Avance
PROBHD    Z159656_0020 (
PULPROG               zg30
TD                   65536
SOLVENT              CD3CN
NS                      16
DS                       2
SWH              11904.762 Hz
FIDRES            0.363304 Hz
AQ               2.7525120 sec
RG                 84.9821
DW                  42.000 usec
DE                   22.00 usec
TE                   298.0 K
D1              1.00000000 sec
TD0                      1
SFO1           600.4230021 MHz
NUC1                    1H
P0                    4.00 usec
P1                   12.00 usec
PLW1           13.51200008 W

F2 - Processing parameters
SI                   65536
SF             600.4200171 MHz
WDW                     EM
SSB                      0
LB                    0.30 Hz
GB                       0
PC                    1.00
```

(*E*)-3-(4-(((2-(2-Methyl-1*H*-indol-3-yl)ethyl)amino)methyl)phenyl)-*N*-((2-nitro-1-(2-oxo-2-((2,2,3,3,3-pentafluoropropyl)amino)ethyl)-1*H*-imidazol-5-yl)methoxy)acrylamide (2) ^{13}C NMR spectrum

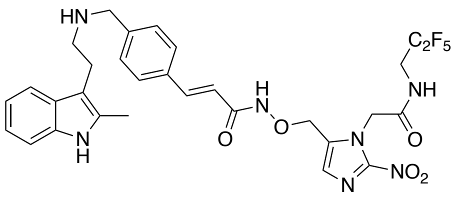

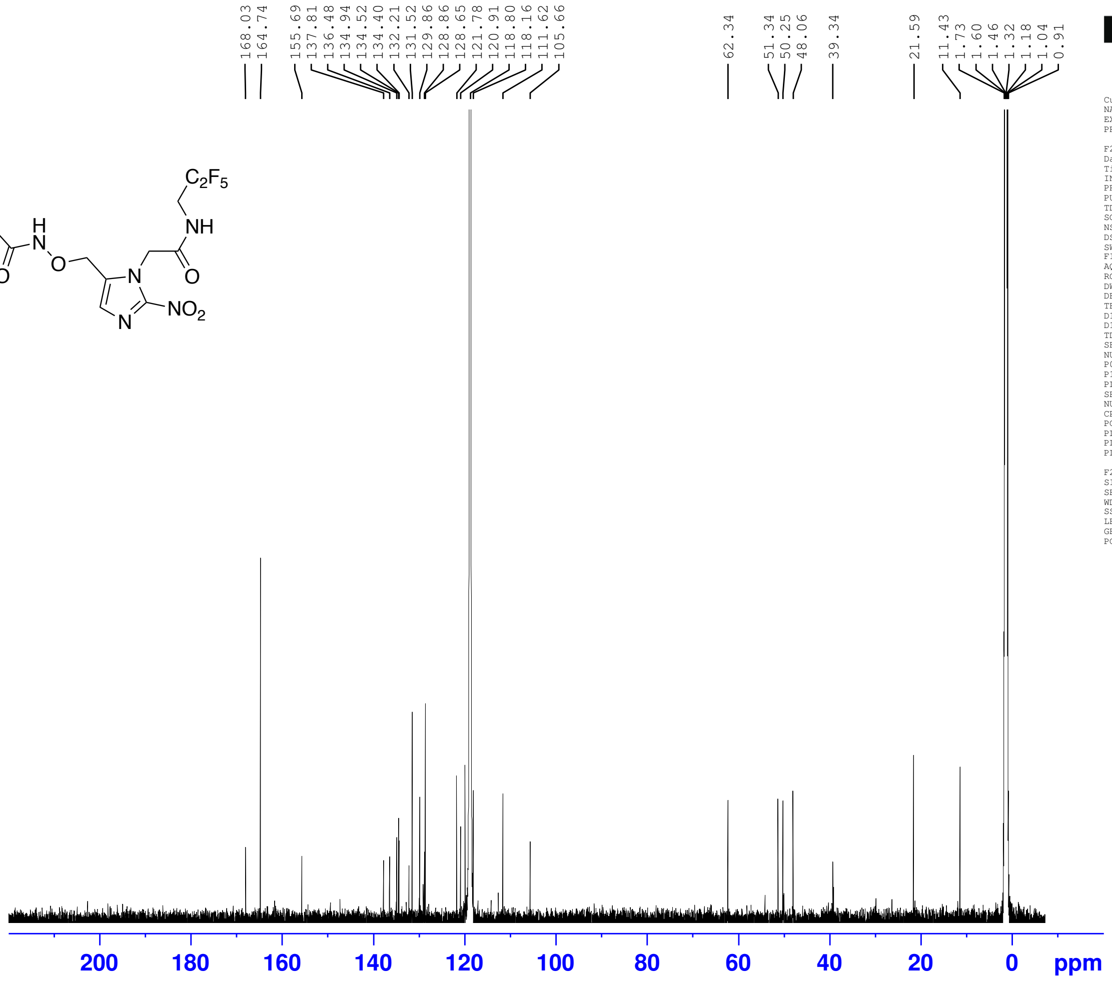


```
Current Data Parameters
NAME     EF5 Pano Service - 7030 ACN-H20 +0.1% FA
EXPNO                   5
PROCNO                  1

F2 - Acquisition Parameters
Date_            20220617
Time                13.08 h
INSTRUM            Avance
PROBHD   Z159656_0020 (
PULPROG            zgpg30
TD                  65536
SOLVENT             CD3CN
NS                    512
DS                      4
SWH             35714.285 Hz
FIDRES           1.089913 Hz
AQ              0.9175040 sec
RG                    101
DW                 14.000 usec
DE                  18.00 usec
TE                  298.0 K
D1             2.00000000 sec
D11            0.03000000 sec
TD0                     1
SFO1          150.9923364 MHz
NUC1                  13C
P0                   3.33 usec
P1                  10.00 usec
PLW1          41.91400146 W
SFO2          600.4224017 MHz
NUC2                   1H
CPDPRG[2         waltz16
PCPD2               80.00 usec
PLW2          13.51200008 W
PLW12          0.30124050 W
PLW13          0.15098180 W

F2 - Processing parameters
SI                  65536
SF            150.9755779 MHz
WDW                    EM
SSB                     0
LB                   1.00 Hz
GB                      0
PC                   1.40
```

(*E*)-3-(4-(((2-(2-Methyl-1*H*-indol-3-yl)ethyl)amino)methyl)phenyl)-*N*-((2-nitro-1-(2-oxo-2-((2,2,3,3,3-pentafluoropropyl)amino)ethyl)-1*H*-imidazol-5-yl)methoxy)acrylamide (2) ^{19}F NMR spectrum

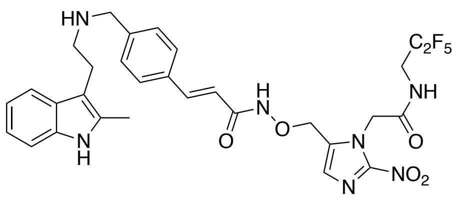

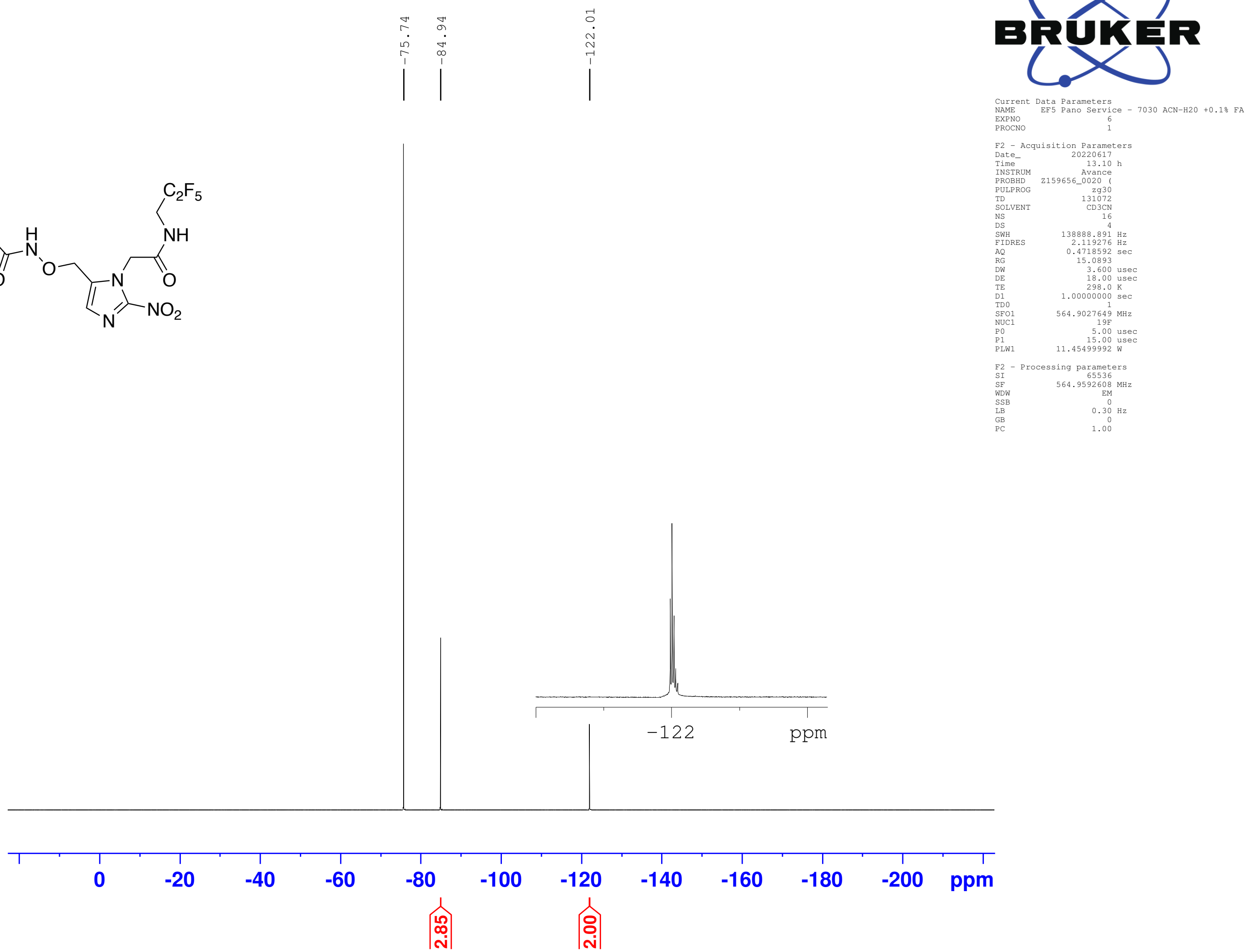

(*E*)-3-(4-(((2-(2-Methyl-1*H*-indol-3-yl)ethyl)amino)methyl)phenyl)-*N*-((2-nitro-1-(2-oxo-2-((2,2,3,3,3-pentafluoropropyl)amino)ethyl)-1*H*-imidazol-5-yl)methoxy)acrylamide (2) ^{1}H NMR spectrum (D_6-DMSO)

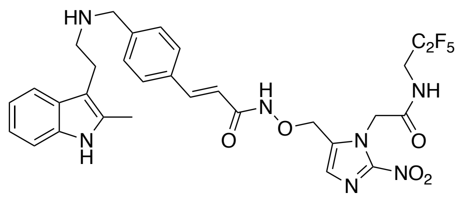

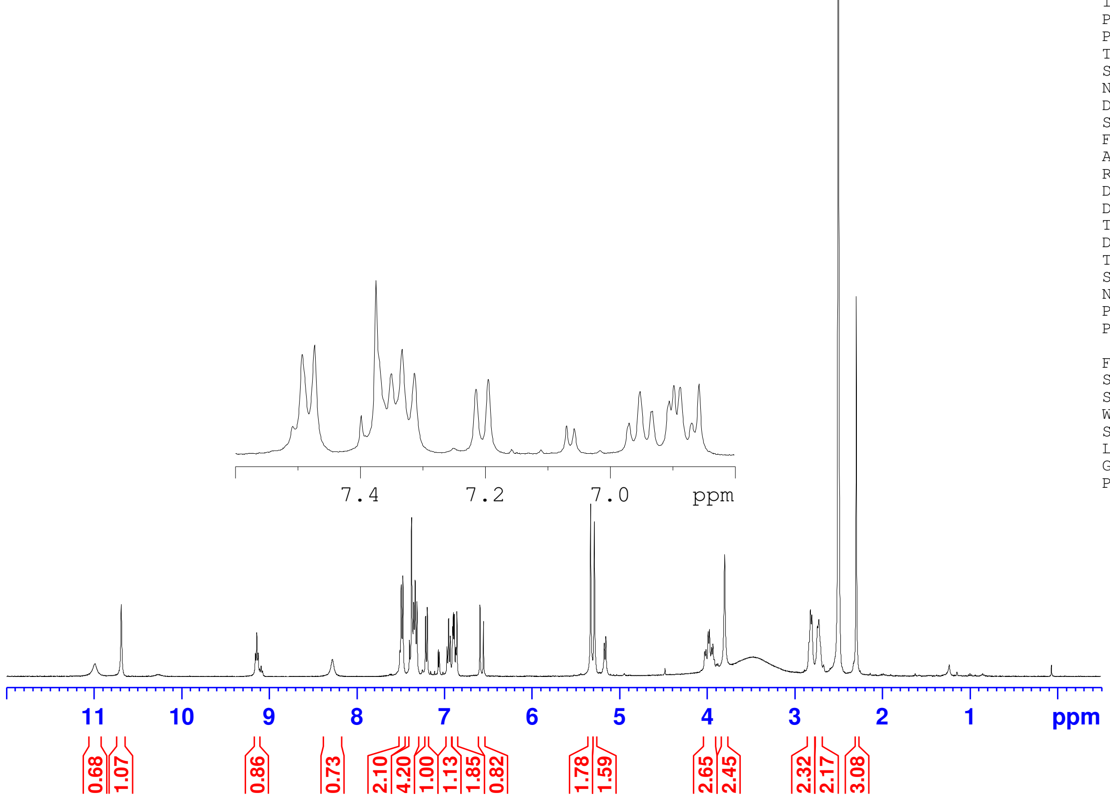


```
Current Data Parameters
NAME      EF5-Pano in DMSO
EXPNO                    1
PROCNO                   1

F2 - Acquisition Parameters
Date_             20220523
Time                 10.03 h
INSTRUM             avg400
PROBHD   Z108618_0816 (
PULPROG               zg60
TD                   65536
SOLVENT               DMSO
NS                      16
DS                       2
SWH               8012.820 Hz
FIDRES            0.244532 Hz
AQ               4.0894465 sec
RG                  162.47
DW                  62.400 usec
DE                    6.50 usec
TE                   298.0 K
D1              1.00000000 sec
TD0                      1
SFO1           400.2024012 MHz
NUC1                    1H
P1                   14.00 usec
PLW1           14.00000000 W

F2 - Processing parameters
SI                   32768
SF             400.2000030 MHz
WDW                     EM
SSB                      0
LB                    0.30 Hz
GB                       0
PC                    1.00
```

Ethyl 2-nitro-1-{2'-oxo-2'-[(2'',2'',3'',3'',3''-pentafluoropropyl)amino]ethyl}-1*H*-imidazole-5-carboxylate (13) ^{1}H NMR spectrum

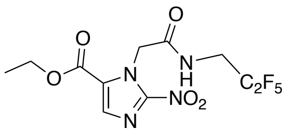

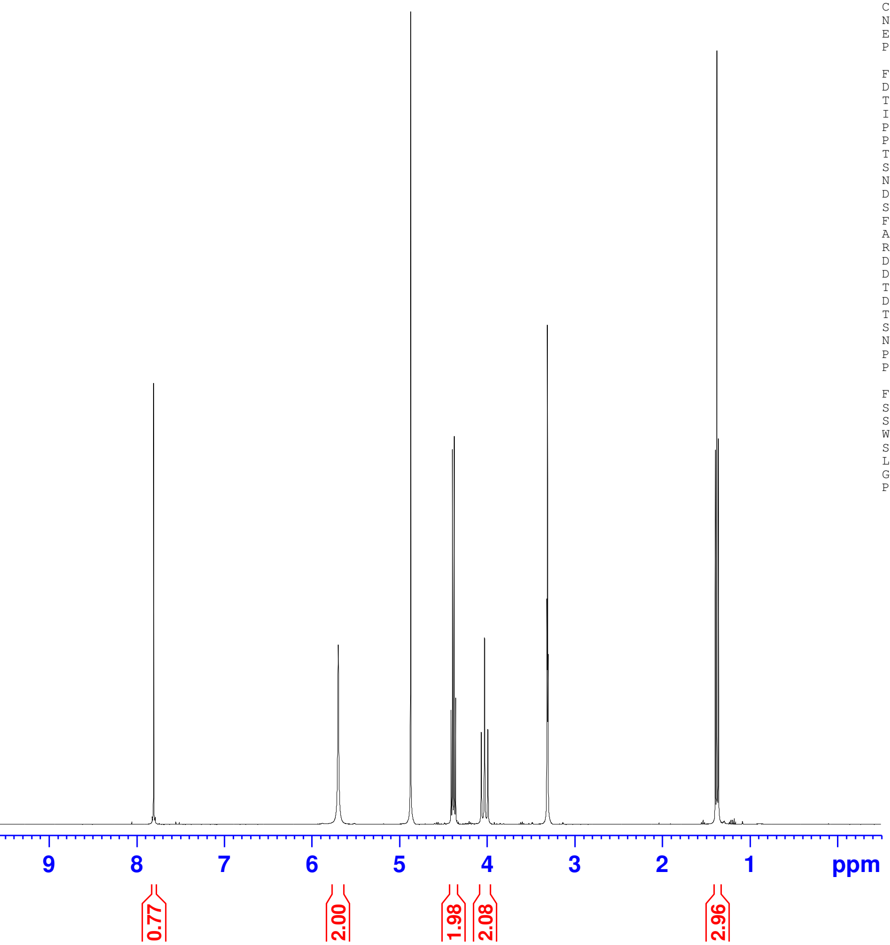


```
Current Data Parameters
NAME      Jul12-2022-4-MLJM-NitroImid
EXPNO                 1
PROCNO                1

F2 - Acquisition Parameters
Date_          20220712
Time              19.18 h
INSTRUM          avh400
PROBHD   Z116098_0219 (
PULPROG            zg60
TD                65536
SOLVENT            MeOD
NS                   16
DS                    2
SWH            8012.820 Hz
FIDRES         0.244532 Hz
AQ            4.0894465 sec
RG                88.17
DW               62.400 usec
DE                 6.50 usec
TE                295.8 K
D1           1.00000000 sec
TD0                   1
SFO1        400.1324008 MHz
NUC1                 1H
P1                10.00 usec
PLW1        16.00000000 W

F2 - Processing parameters
SI                32768
SF          400.1300077 MHz
WDW                  EM
SSB                   0
LB                 0.30 Hz
GB                    0
PC                 1.00
```

Ethyl 2-nitro-1-{2'-oxo-2'-[(2'',2'',3'',3'',3''-pentafluoropropyl)amino]ethyl}-1*H*-imidazole-5-carboxylate (13) ^{13}C NMR spectrum

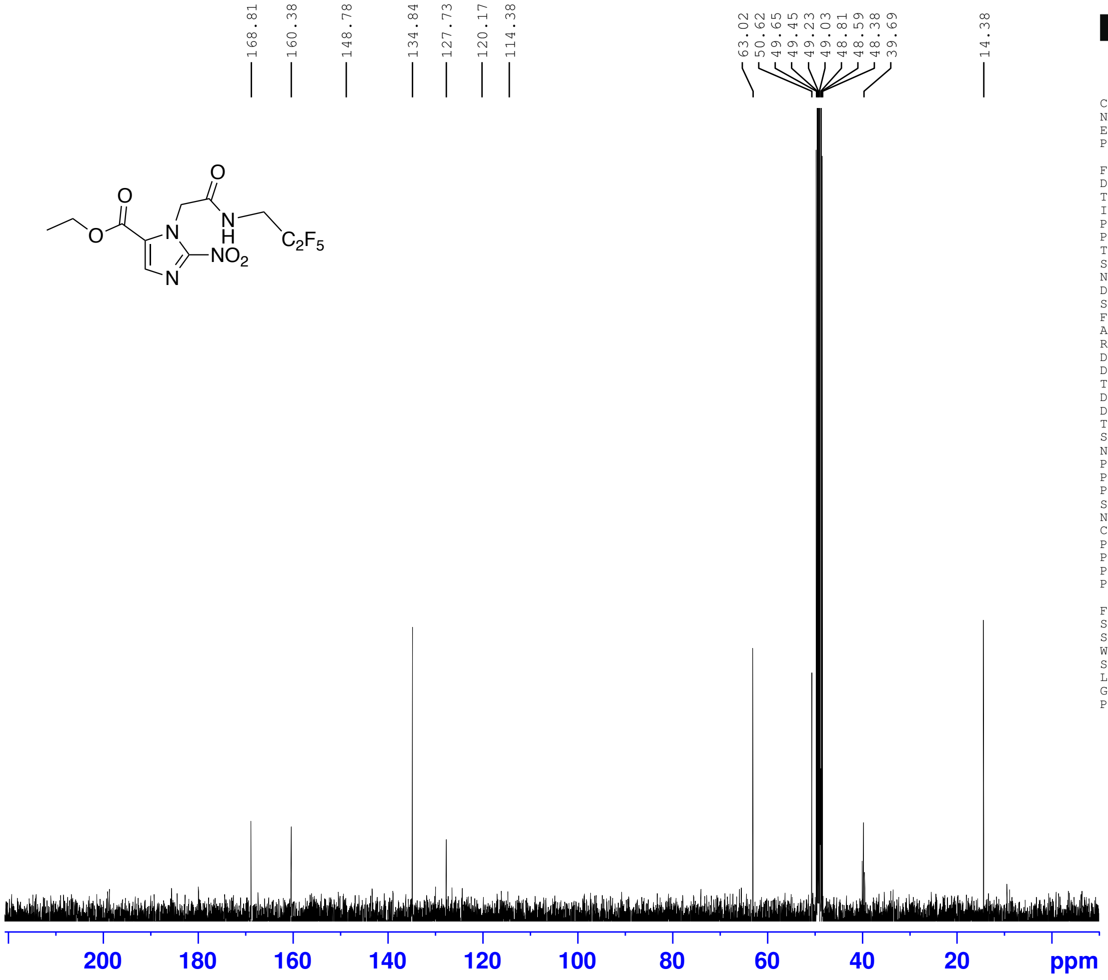


```
Current Data Parameters
NAME      Jul12-2022-4-MLJM-NitroImid
EXPNO                   2
PROCNO                  1

F2 - Acquisition Parameters
Date_            20220713
Time                 9.13 h
INSTRUM            avh400
PROBHD    Z116098_0219 (
PULPROG            zgpg30
TD                  32768
SOLVENT              MeOD
NS                    512
DS                      4
SWH             26041.666 Hz
FIDRES           1.589457 Hz
AQ              0.6291456 sec
RG                 197.18
DW                 19.200 usec
DE                   6.50 usec
TE                  296.7 K
D1             1.00000000 sec
D11            0.03000000 sec
TD0                     1
SFO1          100.6228298 MHz
NUC1                  13C
P0                   3.67 usec
P1                  11.00 usec
PLW1          69.00000000 W
SFO2          400.1316005 MHz
NUC2                   1H
CPDPRG[2          waltz16
PCPD2               90.00 usec
PLW2          16.00000000 W
PLW12          0.19753000 W
PLW13          0.09935700 W

F2 - Processing parameters
SI                  32768
SF            100.6126267 MHz
WDW                    EM
SSB                     0
LB                   1.00 Hz
GB                      0
PC                   1.40
```

Ethyl 2-nitro-1-{2'-oxo-2'-[(2'',2'',3'',3'',3''-pentafluoropropyl)amino]ethyl}-1*H*-imidazole-5-carboxylate (13) partial ^{13}C NMR spectrum

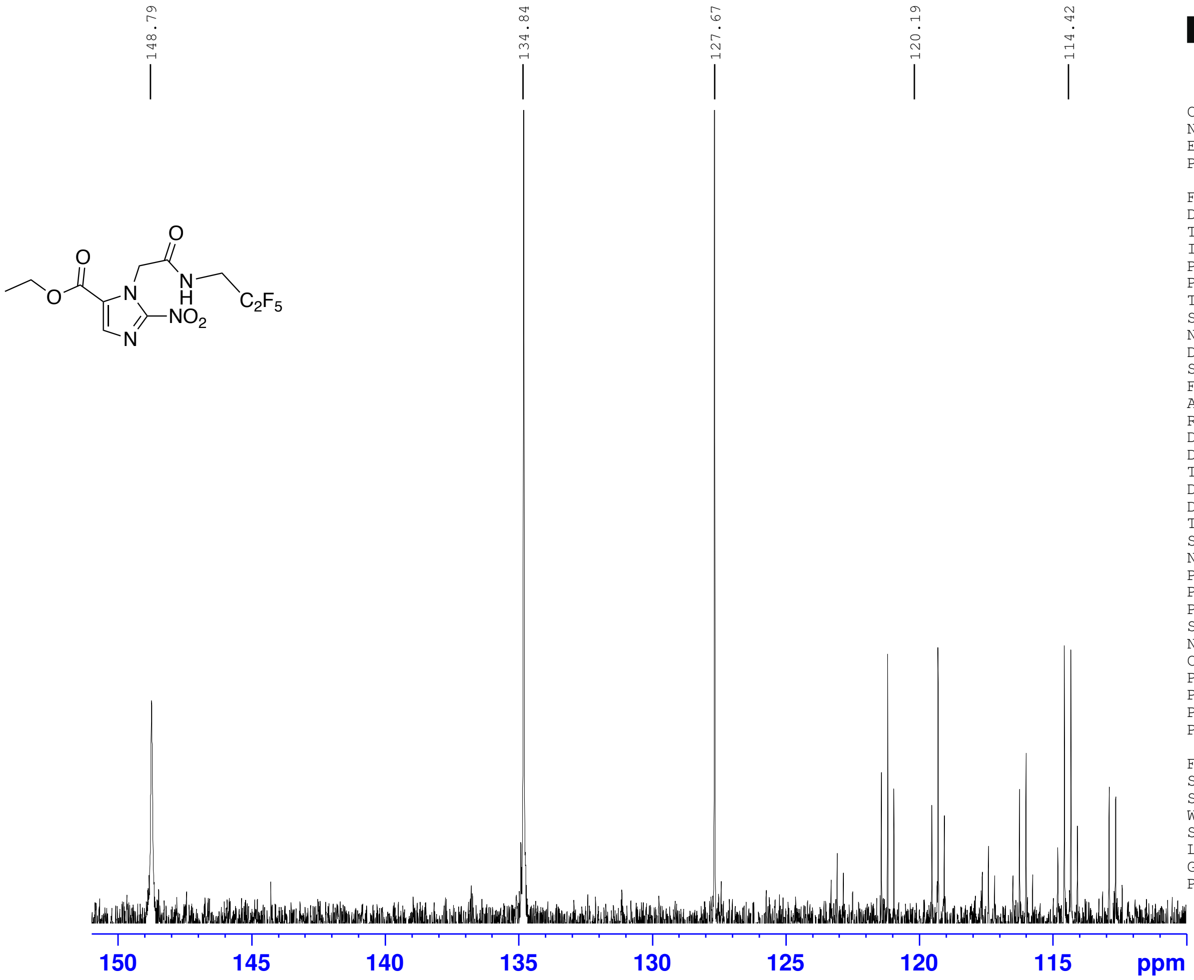


```
Current Data Parameters
NAME       NitroImid Service
EXPNO                      2
PROCNO                     1

F2 - Acquisition Parameters
Date_               20220715
Time                    2.10 h
INSTRUM               Avance
PROBHD     Z159656_0020 (
PULPROG              zgpg30
TD                     65536
SOLVENT                 MeOD
NS                      1024
DS                         4
SWH                35714.285 Hz
FIDRES              1.089913 Hz
AQ                 0.9175040 sec
RG                       101
DW                    14.000 usec
DE                     18.00 usec
TE                     298.0 K
D1                2.00000000 sec
D11               0.03000000 sec
TD0                        1
SFO1             150.9923364 MHz
NUC1                     13C
P0                      3.33 usec
P1                     10.00 usec
PLW1             41.91400146 W
SFO2             600.4224017 MHz
NUC2                      1H
CPDPRG[2             waltz16
PCPD2                  80.00 usec
PLW2             13.51200008 W
PLW12             0.30124050 W
PLW13             0.15098180 W

F2 - Processing parameters
SI                     65536
SF               150.9755162 MHz
WDW                       EM
SSB                        0
LB                      1.00 Hz
GB                         0
PC                      1.40
```

Ethyl 2-nitro-1-{2'-oxo-2'-[(2'',2'',3'',3'',3''-pentafluoropropyl)amino]ethyl}-1*H*-imidazole-5-carboxylate (13) ^{19}F NMR spectrum

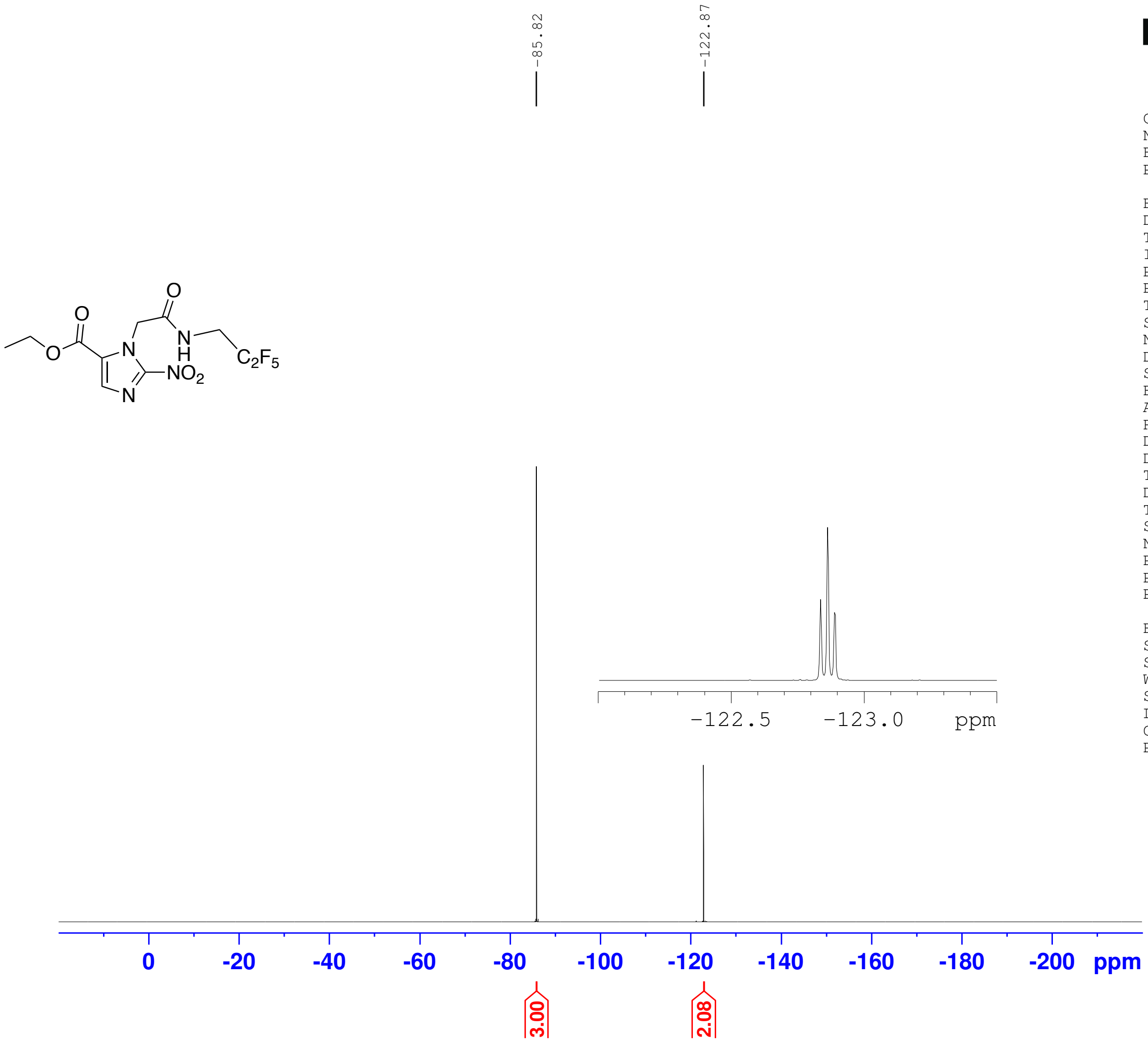

BRUKER

Current Data Parameters
NAME NitroImid Service
EXPNO 3
PROCNO 1

F2 - Acquisition Parameters
Date_ 20220715
Time 2.12 h
INSTRUM Avance
PROBHD Z159656_0020 (
PULPROG zg30
TD 131072
SOLVENT MeOD
NS 16
DS 4
SWH 138888.891 Hz
FIDRES 2.119276 Hz
AQ 0.4718592 sec
RG 9.02778
DW 3.600 usec
DE 18.00 usec
TE 298.0 K
D1 1.00000000 sec
TD0 1
SFO1 564.9027649 MHz
NUC1 19F
P0 5.00 usec
P1 15.00 usec
PLW1 11.45499992 W

F2 - Processing parameters
SI 65536
SF 564.9592608 MHz
WDW EM
SSB 0
LB 0.30 Hz
GB 0
PC 1.00

2'-[5-(Hydroxymethyl)-2-nitro-1*H*-imidazol-1-yl]-*N*-(2",2",3",3",3"-pentafluoropropyl)acetamide (14) ^{1}H NMR spectrum

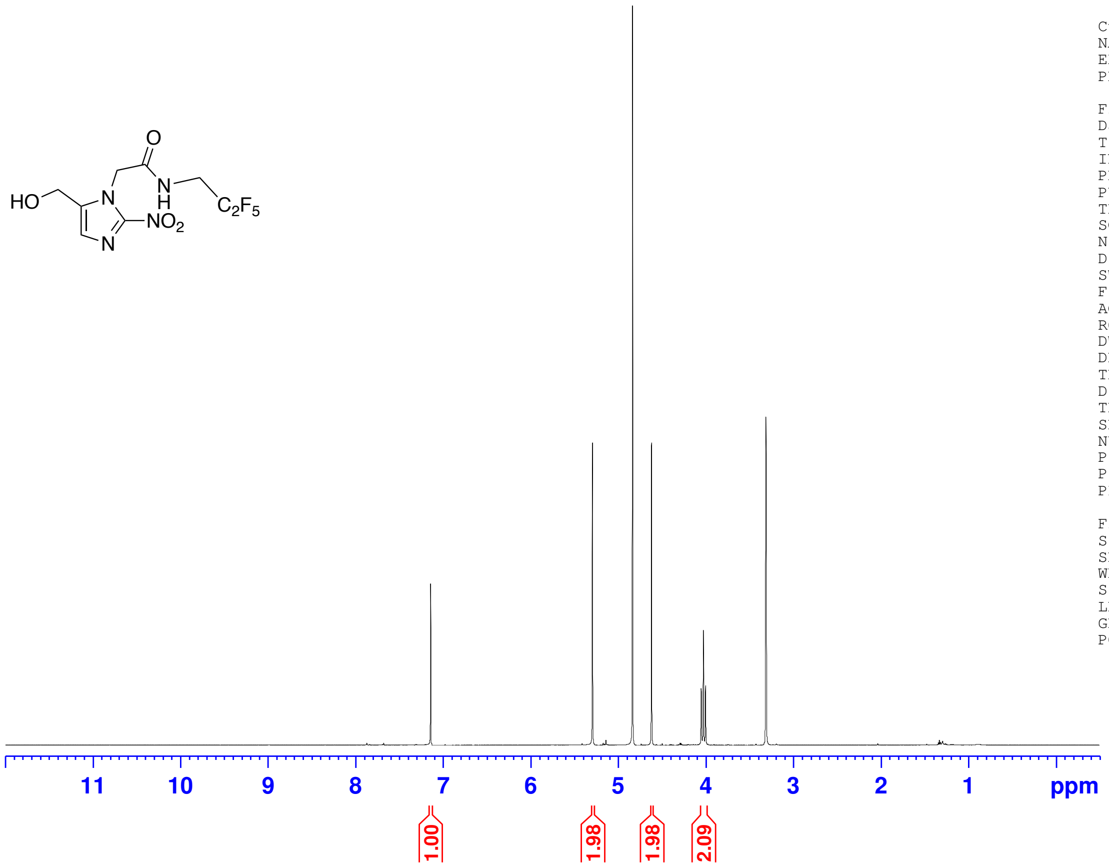


```
Current Data Parameters
NAME      NitroAlcohol Service
EXPNO                    1
PROCNO                   1

F2 - Acquisition Parameters
Date_             20220715
Time                  2.49 h
INSTRUM             Avance
PROBHD    Z159656_0020 (
PULPROG               zg30
TD                   65536
SOLVENT               MeOD
NS                      16
DS                       2
SWH              11904.762 Hz
FIDRES            0.363304 Hz
AQ               2.7525120 sec
RG                     101
DW                  42.000 usec
DE                   22.00 usec
TE                   298.0 K
D1              1.00000000 sec
TD0                      1
SFO1           600.4230021 MHz
NUC1                    1H
P0                    4.00 usec
P1                   12.00 usec
PLW1           13.51200008 W

F2 - Processing parameters
SI                   65536
SF             600.4200111 MHz
WDW                     EM
SSB                      0
LB                    0.30 Hz
GB                       0
PC                    1.00
```

2'-[5-(Hydroxymethyl)-2-nitro-1*H*-imidazol-1-yl]-*N*-(2",2",3",3",3"-pentafluoropropyl)acetamide (14) ^{13}C NMR spectrum

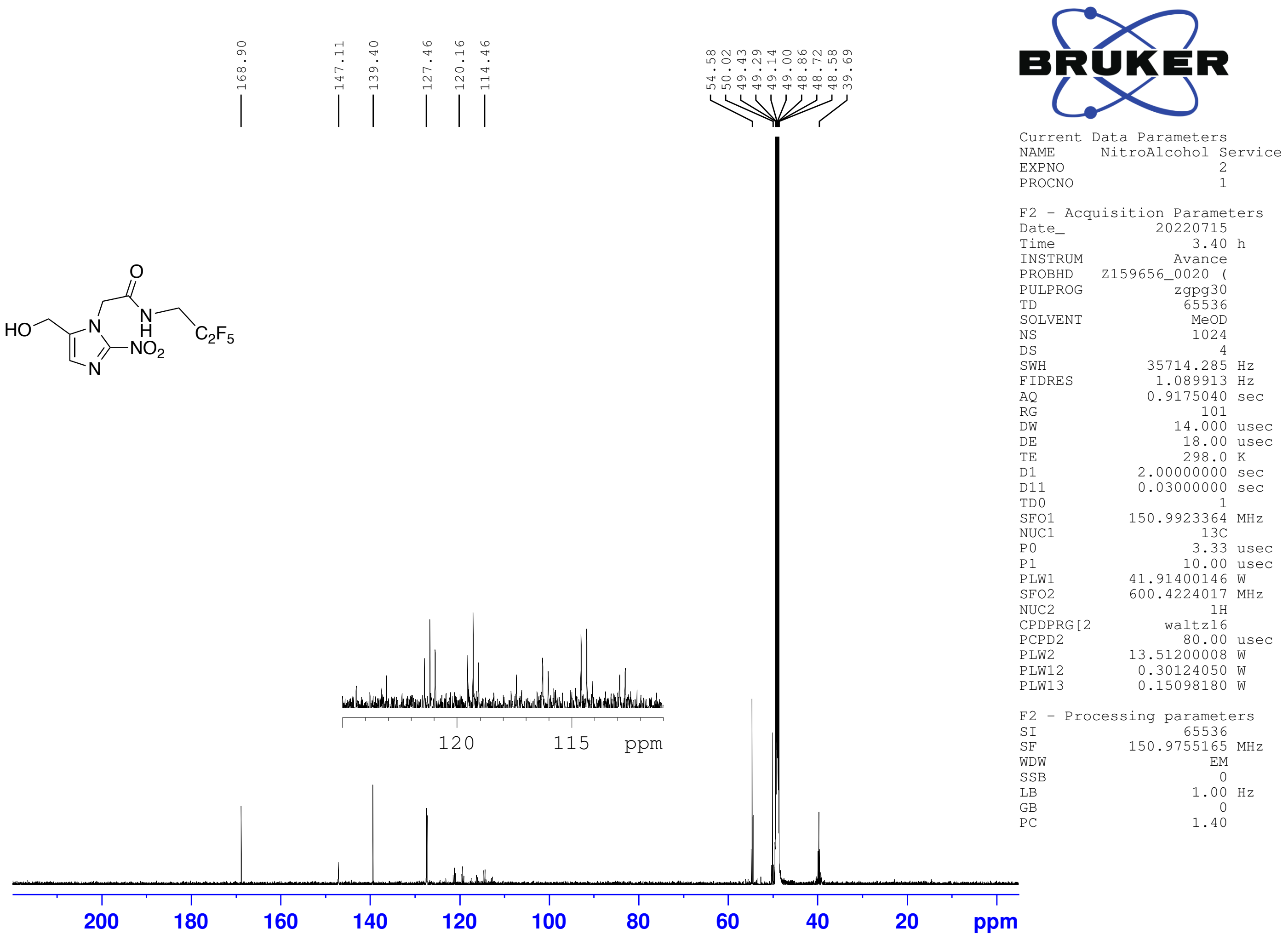

2'-[5-(Hydroxymethyl)-2-nitro-1*H*-imidazol-1-yl]-*N*-(2",2",3",3",3"-pentafluoropropyl)acetamide (14) ^{19}F NMR spectrum

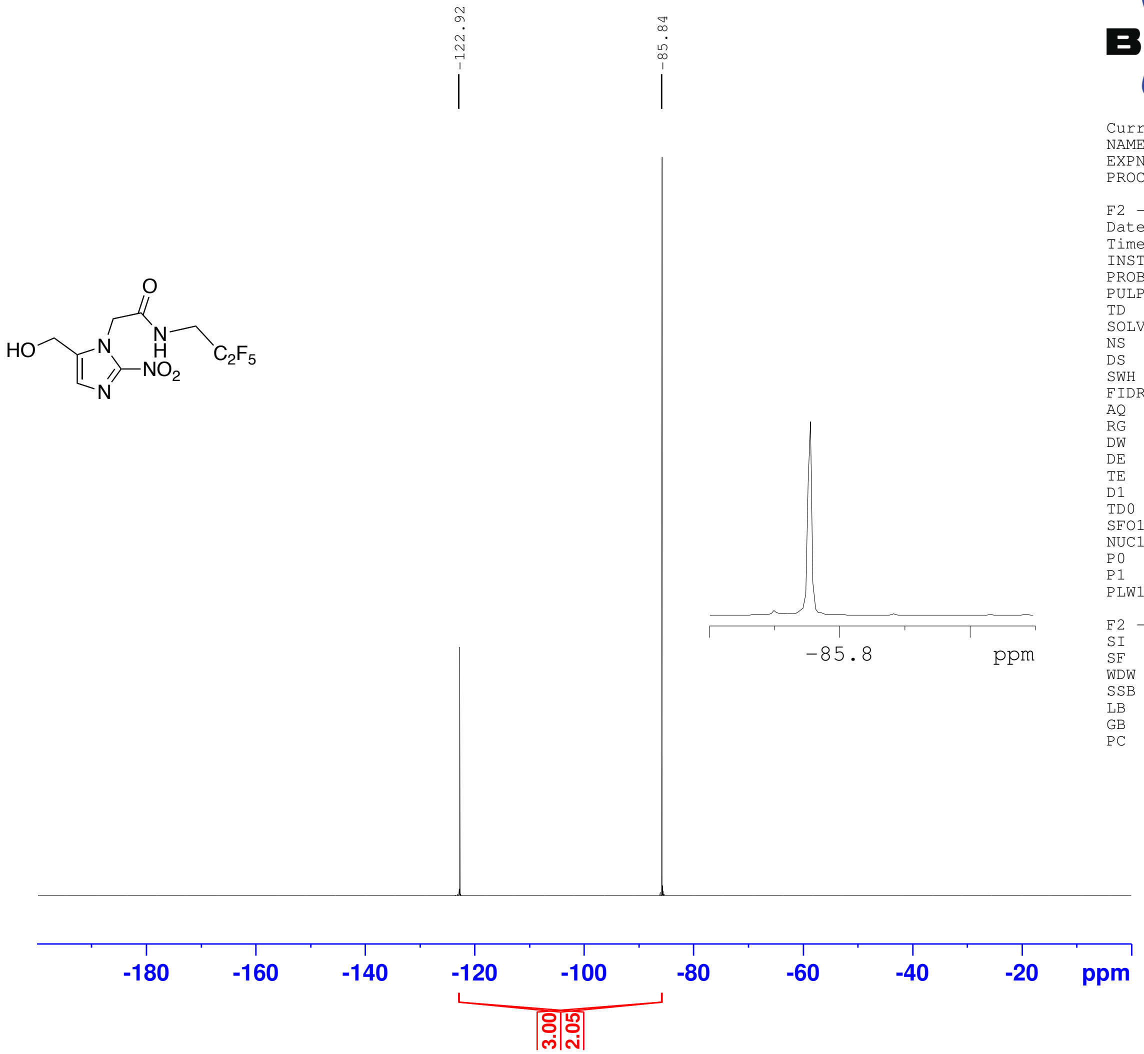

Current Data Parameters
NAME NitroAlcohol Service
EXPNO 3
PROCNO 1

F2 - Acquisition Parameters
Date_ 20220715
Time 3.41 h
INSTRUM Avance
PROBHD Z159656_0020 (
PULPROG zg30
TD 131072
SOLVENT MeOD
NS 16
DS 4
SWH 138888.891 Hz
FIDRES 2.119276 Hz
AQ 0.4718592 sec
RG 32.0524
DW 3.600 usec
DE 18.00 usec
TE 298.0 K
D1 1.00000000 sec
TD0 1
SFO1 564.9027649 MHz
NUC1 19F
P0 5.00 usec
P1 15.00 usec
PLW1 11.45499992 W

F2 - Processing parameters
SI 65536
SF 564.9592608 MHz
WDW EM
SSB 0
LB 0.30 Hz
GB 0
PC 1.00

Ethyl 2-amino-1-{2'-oxo-2'-[(2",2",3",3",3"-pentafluoropropyl)amino]ethyl}-1*H*-imidazole-5-carboxylate (12) ^{1}H NMR spectrum

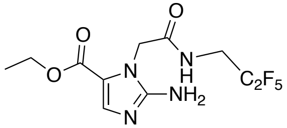

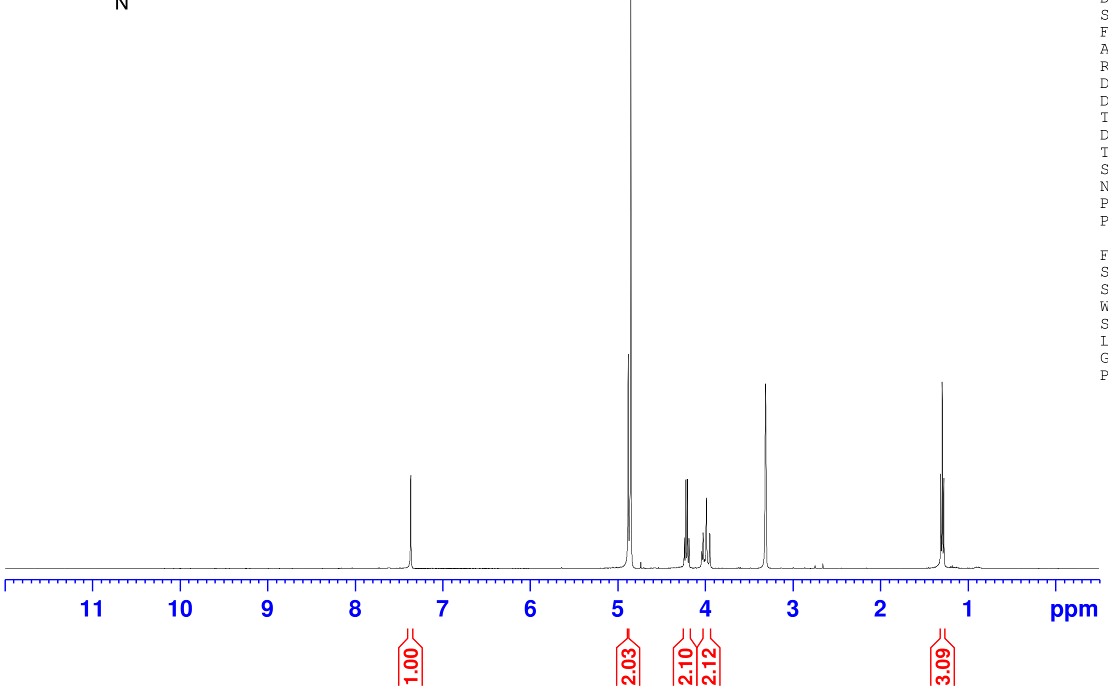


```
Current Data Parameters
NAME              AminoImid
EXPNO                     1
PROCNO                    1

F2 - Acquisition Parameters
Date_              20220714
Time                  11.30 h
INSTRUM              avg400
PROBHD   Z8400_0179 (PH
PULPROG                zg60
TD                    65536
SOLVENT                MeOD
NS                       16
DS                        2
SWH                8012.820 Hz
FIDRES             0.244532 Hz
AQ                4.0894465 sec
RG                   206.87
DW                   62.400 usec
DE                     6.50 usec
TE                    298.0 K
D1               1.00000000 sec
TD0                       1
SFO1            400.2024012 MHz
NUC1                     1H
P1                    11.00 usec
PLW1            14.00000000 W

F2 - Processing parameters
SI                    32768
SF              400.2000077 MHz
WDW                      EM
SSB                       0
LB                     0.30 Hz
GB                        0
PC                     1.00
```

Ethyl 2-amino-1-{2'-oxo-2'-[(2'',2'',3'',3'',3''-pentafluoropropyl)amino]ethyl}-1*H*-imidazole-5-carboxylate (12) ^{13}C NMR spectrum

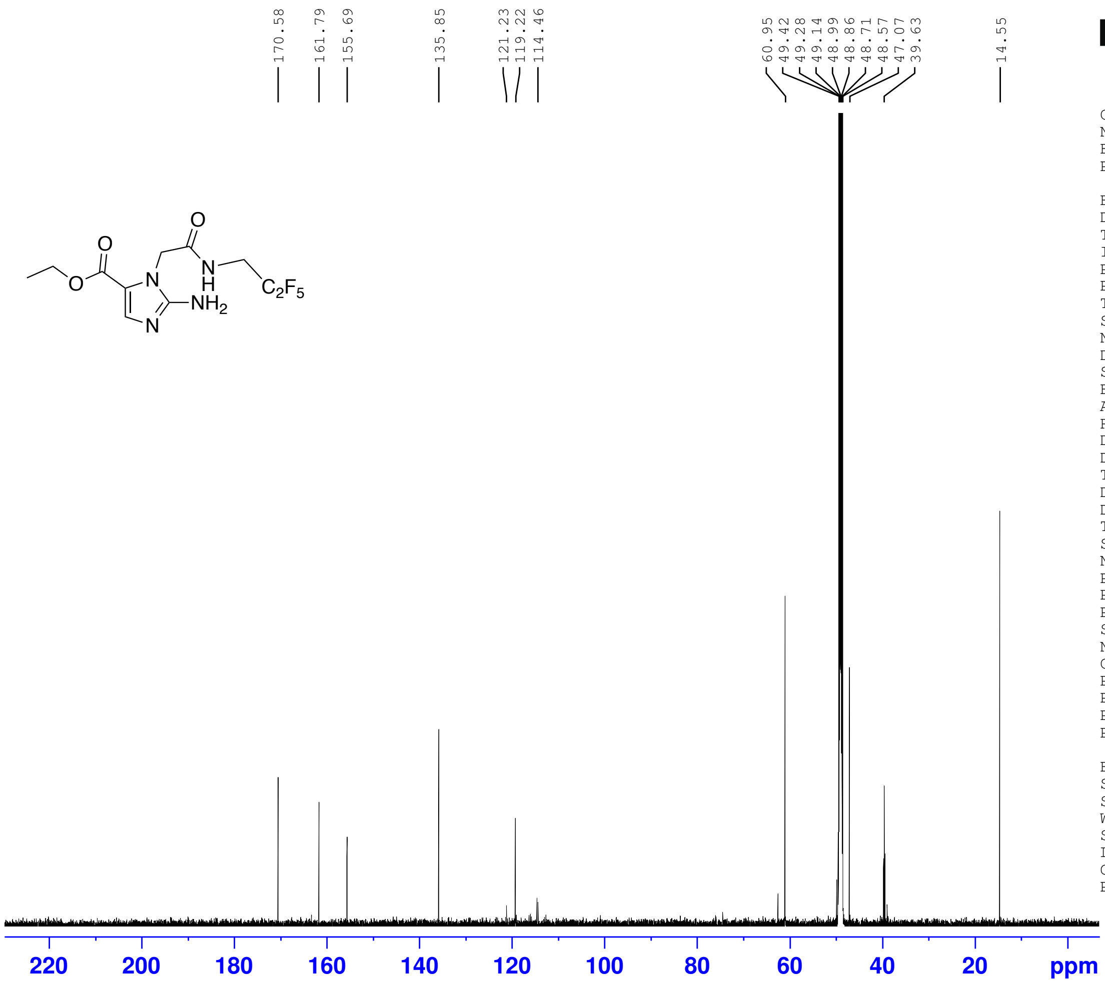

Current Data Parameters
NAME AminoImid Service
EXPNO 2
PROCNO 1

F2 - Acquisition Parameters
Date_ 20220715
Time 18.52 h
INSTRUM Avance
PROBHD Z159656_0020 (
PULPROG zgpg30
TD 65536
SOLVENT MeOD
NS 512
DS 4
SWH 35714.285 Hz
FIDRES 1.089913 Hz
AQ 0.9175040 sec
RG 101
DW 14.000 usec
DE 18.00 usec
TE 298.0 K
D1 2.00000000 sec
D11 0.03000000 sec
TD0 1
SFO1 150.9923364 MHz
NUC1 13C
P0 3.33 usec
P1 10.00 usec
PLW1 41.91400146 W
SFO2 600.4224017 MHz
NUC2 1H
CPDPRG[2 waltz16
PCPD2 80.00 usec
PLW2 13.51200008 W
PLW12 0.30124050 W
PLW13 0.15098180 W

F2 - Processing parameters
SI 65536
SF 150.9755174 MHz
WDW EM
SSB 0
LB 1.00 Hz
GB 0
PC 1.40

Ethyl 2-amino-1-{2'-oxo-2'-[(2'',2'',3'',3'',3''-pentafluoropropyl)amino]ethyl}-1*H*-imidazole-5-carboxylate (5) ^{19}F NMR spectrum

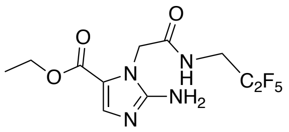

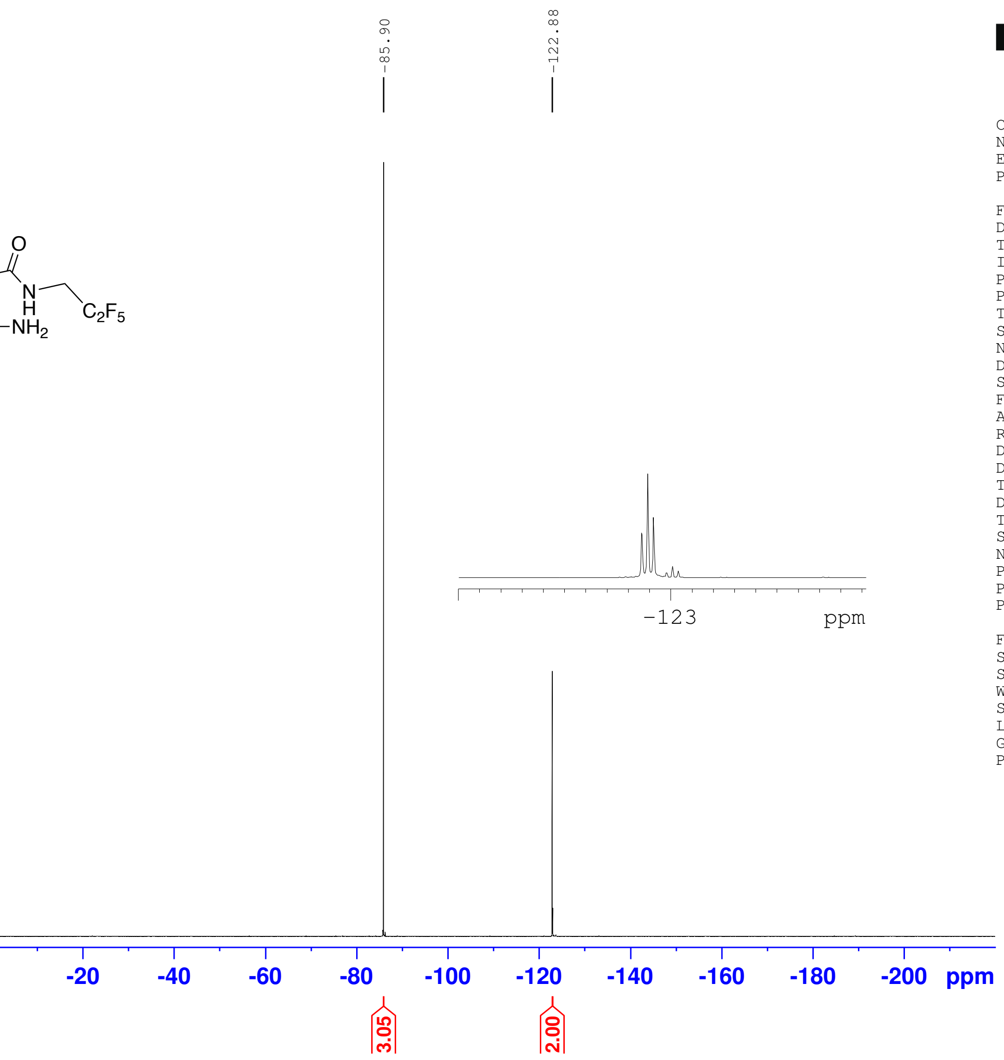


```
Current Data Parameters
NAME      AminoImid Service
EXPNO                     3
PROCNO                    1

F2 - Acquisition Parameters
Date_              20220715
Time                  18.54 h
INSTRUM              Avance
PROBHD   Z159656_0020 (
PULPROG                zg30
TD                   131072
SOLVENT                MeOD
NS                       16
DS                        4
SWH              138888.891 Hz
FIDRES             2.119276 Hz
AQ                0.4718592 sec
RG                  9.51577
DW                    3.600 usec
DE                    18.00 usec
TE                    298.0 K
D1               1.00000000 sec
TD0                       1
SFO1            564.9027649 MHz
NUC1                    19F
P0                     5.00 usec
P1                    15.00 usec
PLW1            11.45499992 W

F2 - Processing parameters
SI                    65536
SF              564.9592608 MHz
WDW                      EM
SSB                       0
LB                     0.30 Hz
GB                        0
PC                     1.00
```

Ethyl 2-amino-1-{2'-oxo-2'-[(2'',2'',3'',3'',3''-pentafluoropropyl)amino]ethyl}-1*H*-imidazole-5-carboxylate (5) HMBC spectrum

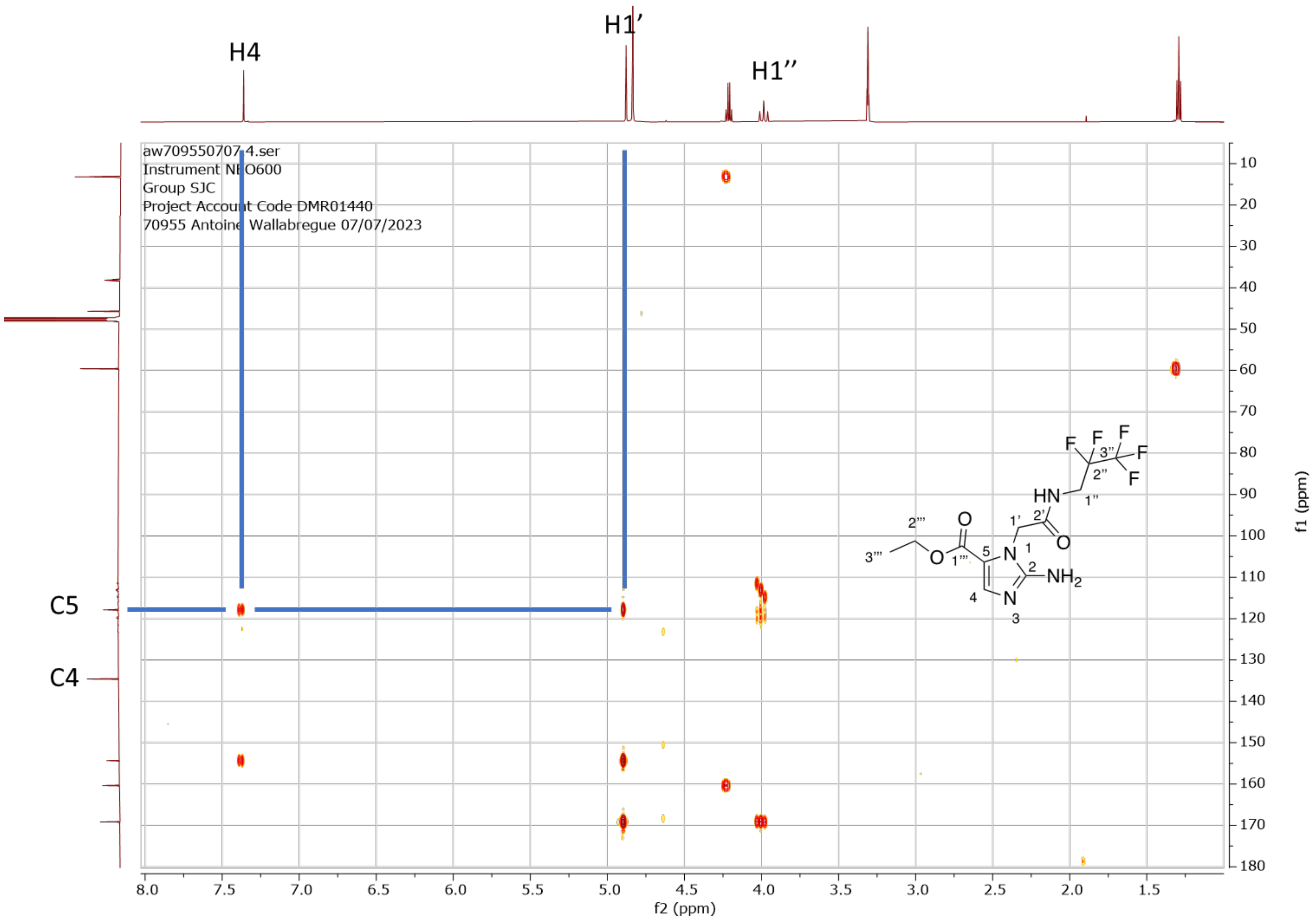

5. HPLC Traces

2-(5-((4-Formylphenoxy)methyl)-2-nitro-1*H*-imidazol-1-yl)-*N*-(2,2,3,3,3-pentafluoropropyl)acetamide (16)

Area Percent Report

MLJM B49 Post Service

Agilent

**Data file:**	Q:\Analytical HPLC\Data\Max Mallerman\MLJM B49 Post Service 2021-12-06 09-42-17\001-P1-F1-MLJM B49 Post Service.D		
**Sample name:**	MLJM B49 Post Service		
**Description:**			
**Sample amount:**	0.000	**Sample type:**	Sample
**Instrument:**	Analytical HPLC	**Location:**	P1-F1
**Injection date:**	2021-12-06 09:45:15+00:00	**Injection:**	1 of 1
**Acq. method:**	IOD_PURITY_TEST.M	**Injection volume:**	20.000
**Analysis method:**	IOD_PURITY_TEST.M	**Acq. operator:**	SYSTEM
**Last changed:**	2022-07-06 13:57:38+01:00		

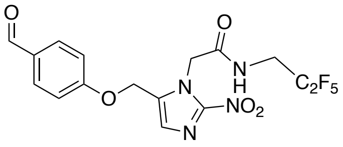

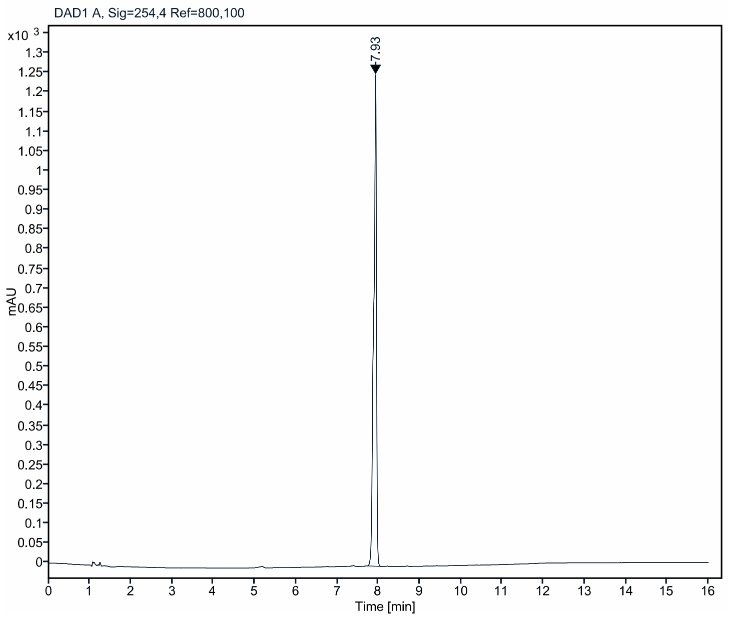

Signal: DAD1 A, Sig=254,4 Ref=800,100

RT [min]	Type	Width [min]	Area	Height	Area%	Name
7.930	PM	0.0769	5793.6343	1255.0706	100.0000	
		Sum	5793.6343			

MLJM Template short.rdl [Rev. 2] Printed: 2022-07-06 13:58:58+01:00 Page 1 of 1

(*E*)-2-(5-((4-(2-(4-(Dicyanomethylene)-4*H*-chromen-2-yl)vinyl)phenoxy)methyl)-2-nitro-1*H*-imidazol-1-yl)-*N*-(2,2,3,3,3-pentafluoropropyl)acetamide (4)

Area Percent Report

MLJM B50 EtOH 2_conc

Agilent

**Data file:**	Q:\Analytical HPLC\Data\Max Mallerman\MLJM B50 EtOH 2_conc 2021-11-30 14-02-46\001-P1-E1-MLJM B50 EtOH 2_conc.D		
**Sample name:**	MLJM B50 EtOH 2_conc		
**Description:**			
**Sample amount:**	0.000	**Sample type:**	Blank
**Instrument:**	Analytical HPLC	**Location:**	P1-E1
**Injection date:**	2021-11-30 14:03:47+00:00	**Injection:**	1 of 1
**Acq. method:**	IOD_PURITY_TEST.M	**Injection volume:**	20.000
**Analysis method:**	IOD_PURITY_TEST.M	**Acq. operator:**	SYSTEM
**Last changed:**	2022-07-06 13:49:06+01:00		

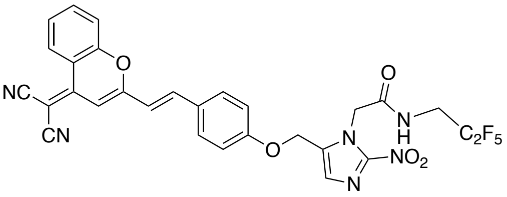

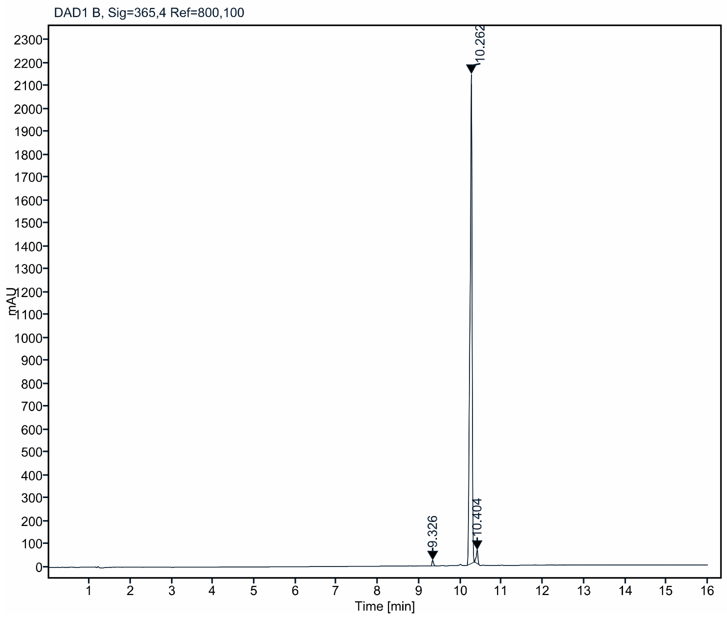

Signal: DAD1 B, Sig=365,4 Ref=800,100

RT [min]	Type	Width [min]	Area	Height	Area%	Name
9.326	MM	0.0415	62.7665	25.1850	0.8135	
10.262	MP	0.0584	7483.9697	2137.2378	96.9958	
10.404	PP	0.0469	169.0285	60.0828	2.1907	
		Sum	7715.7647			

MLJM Template short.rdl [Rev. 2] Printed: 2022-07-06 13:54:51+01:00 Page 1 of 1

tert-Butyl (_E_)-3-(2-((_tert_-butoxycarbonyl)(4-(3-(((2-nitro-1-(2-oxo-2-((2,2,3,3,3-pentafluoropropyl)amino)ethyl)-1_H_-imidazol-5-yl)methoxy)amino)-3-oxoprop-1-en-1-yl)benzyl)amino)ethyl)-2-methyl-1_H_-indole-1-carboxylate

Area Percent Report

MLJM C24 RP PPt

Agilent

**Data file:**	Q:\Analytical HPLC\Data\Max Mallerman\MLJM C24 RP PPt 2022-03-24 15-17-15\001-P2-E2-MLJM C24 RP PPt.D		
**Sample name:**	MLJM C24 RP PPt		
**Description:**			
**Sample amount:**	0.000	**Sample type:**	Sample
**Instrument:**	Analytical HPLC	**Location:**	P2-E2
**Injection date:**	2022-03-24 15:18:33+00:00	**Injection:**	1 of 1
**Acq. method:**	IOD_PURITY_TEST.M	**Injection volume:**	5.000
**Analysis method:**	IOD_PURITY_TEST.M	**Acq. operator:**	SYSTEM
**Last changed:**	2022-07-06 14:11:21+01:00 (modified after loading)		

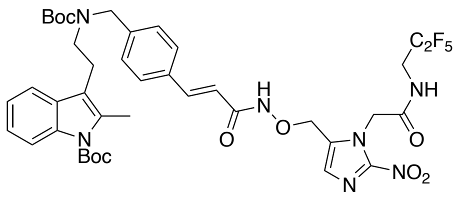

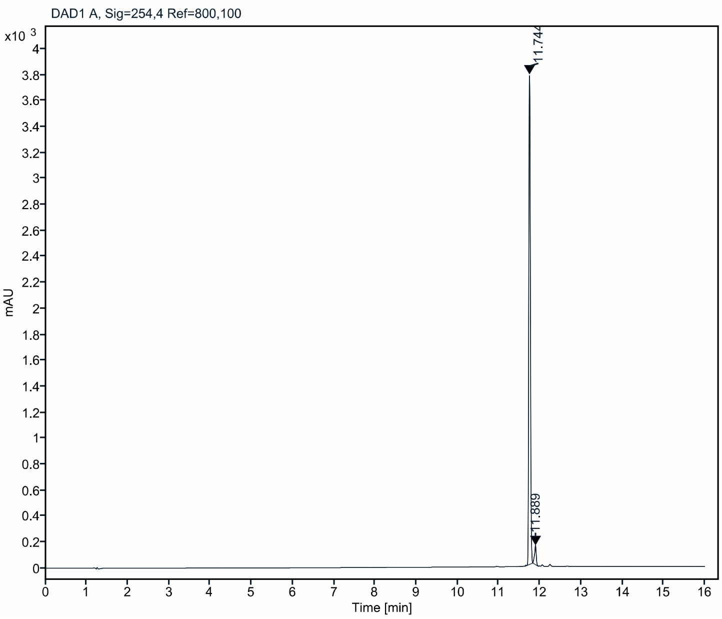

Signal: DAD1 A, Sig=254,4 Ref=800,100

RT [min]	Type	Width [min]	Area	Height	Area%	Name
11.744	MM	0.0483	10946.2119	3776.3796	96.1473	
11.889	MM	0.0488	438.6183	149.8897	3.8527	
		Sum	11384.8302			

(*E*)-3-(4-(((2-(2-Methyl-1*H*-indol-3-yl)ethyl)amino)methyl)phenyl)-*N*-((2-nitro-1-(2-oxo-2-((2,2,3,3,3-pentafluoropropyl)amino)ethyl)-1*H*-imidazol-5-yl)methoxy)acrylamide (2)

Area Percent Report

MLJM C70 Alumina V1

Agilent

**Data file:**	C:\Users\Public\Documents\ChemStation\1\Data\Max Mallerman\MLJM C70 Alumina V1 2022-06-01 16-14-04\001-P1-F4-MLJM C70 Alumina V1.D		
**Sample name:**	MLJM C70 Alumina V1		
**Description:**			
**Sample amount:**	0.000	**Sample type:**	Sample
**Instrument:**	Analytical HPLC	**Location:**	P1-F4
**Injection date:**	2022-06-01 16:16:17+01:00	**Injection:**	1 of 1
**Acq. method:**	IOD_PURITY_TEST.M	**Injection volume:**	20.000
**Analysis method:**	IOD_PURITY_TEST.M	**Acq. operator:**	SYSTEM
**Last changed:**	2022-07-06 14:18:34+01:00 (modified after loading)		

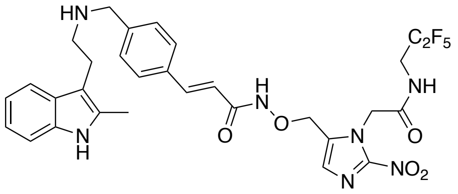

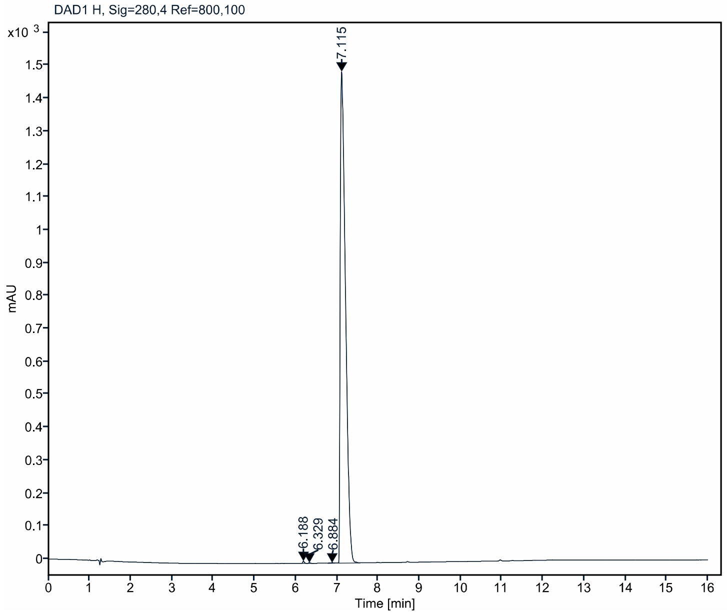

Signal: DAD1 H, Sig=280,4 Ref=800,100

RT [min]	Type	Width [min]	Area	Height	Area%	Name
6.188	PM	0.0466	19.7366	7.0612	0.1496	
6.329	MM	0.0482	7.3503	2.5390	0.0557	
6.884	MM	0.0841	10.2662	2.0345	0.0778	
7.115	MM	0.1467	13153.9951	1494.3236	99.7168	
		Sum	13191.3481			

MLJM Template short.rdl [Rev. 2] Printed: 2022-07-06 14:18:37+01:00 Page 1 of 1

(*E*)-3-(4-(((2-(2-Methyl-1*H*-indol-3-yl)ethyl)amino)methyl)phenyl)-*N*-((2-nitro-1-(2-oxo-2-((2,2,3,3,3-pentafluoropropyl)amino)ethyl)-1*H*-imidazol-5-yl)methoxy)acrylamide (2) (LCMS trace)

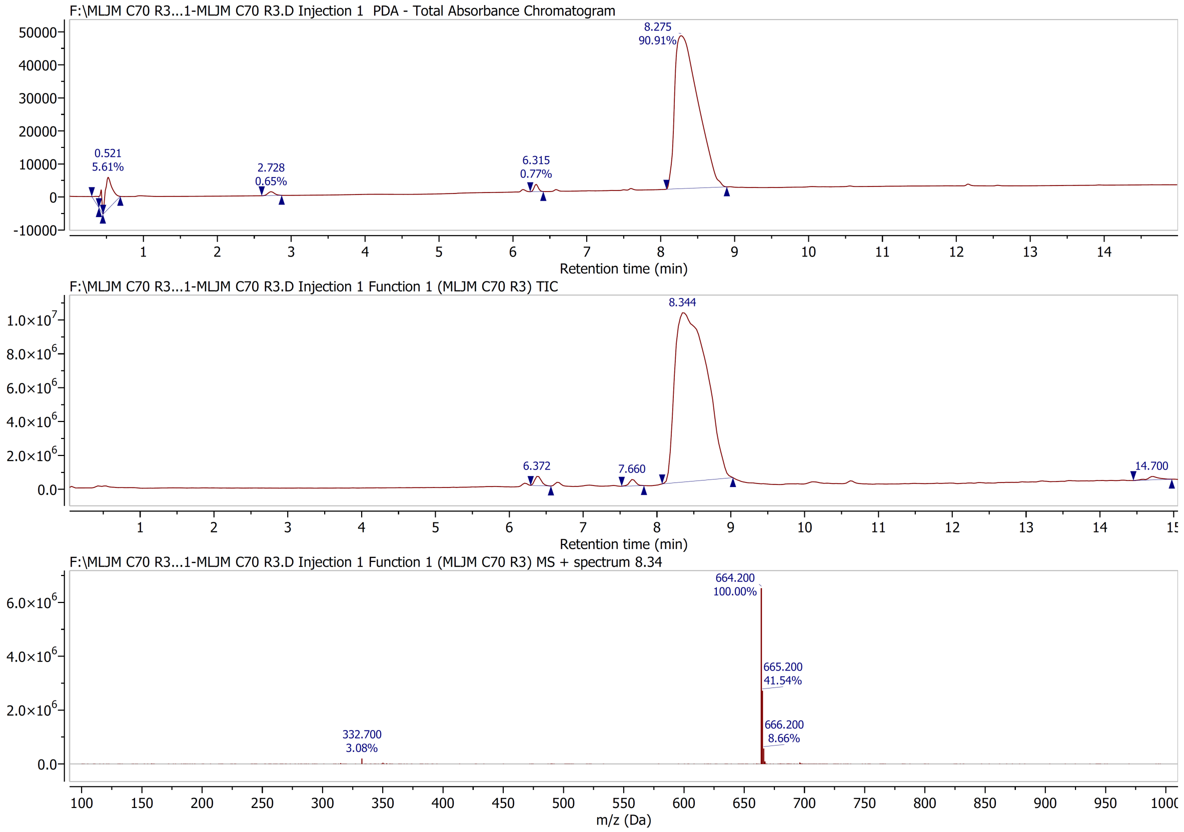

Ethyl 2-nitro-1-{2'-oxo-2'-[(2",2",3",3",3"-pentafluoropropyl)amino]ethyl}-1*H*-imidazole-5-carboxylate (13)

Area Percent Report
MLJM C31

Agilent

**Data file:**	C:\Users\Public\Documents\ChemStation\1\Data\Max Mallerman\MLJM C31 and 32 2022-03-31 15-59-33\002-P2-E3-MLJM C31.D		
**Sample name:**	MLJM C31		
**Description:**			
**Sample amount:**	0.000	**Sample type:**	Sample
**Instrument:**	Analytical HPLC	**Location:**	P2-E3
**Injection date:**	2022-03-31 16:21:16+01:00	**Injection:**	1 of 1
**Acq. method:**	MM_PURITY_CHECK_FA _254_220_280.M	**Injection volume:**	10.000
**Analysis method:**	MM_PURITY_CHECK_FA _254_220_280.M	**Acq. operator:**	SYSTEM
**Last changed:**	2022-07-18 14:14:49+01:00 (modified after loading)		

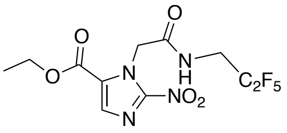

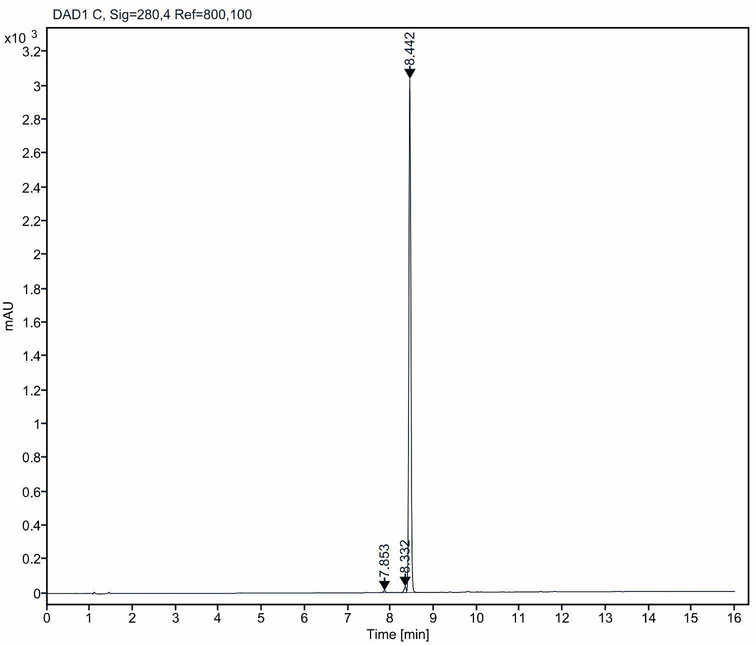

Signal: DAD1 C, Sig=280,4 Ref=800,100

RT [min]	Type	Width [min]	Area	Height	Area%	Name
7.853	BV R	0.0584	49.0929	12.3431	0.5001	
8.332	BV E	0.0480	113.7089	35.8559	1.1583	
8.442	VB R	0.0501	9654.1563	3037.8225	98.3416	
		Sum	9816.9580			

MLJM Template short.rdl [Rev. 2] Printed: 2022-07-18 14:14:52+01:00 Page 1 of 1

2'-[5-(Hydroxymethyl)-2-nitro-1*H*-imidazol-1-yl]-*N*-(2'',2'',3'',3'',3''-pentafluoropropyl)acetamide (14)

Area Percent Report

MLJM C42 recol T30

**Data file:**	C:\Users\Public\Documents\ChemStation\1\Data\Max Mallerman\MLJM C42 Recol T30 2022-04-28 12-14-56\001-P2-D10-MLJM C42 recol T30.D		
**Sample name:**	MLJM C42 recol T30		
**Description:**			
**Sample amount:**	0.000	**Sample type:**	Sample
**Instrument:**	Analytical HPLC	**Location:**	P2-D10
**Injection date:**	2022-04-28 12:15:51+01:00	**Injection:**	1 of 1
**Acq. method:**	IOD_PURITY_TEST.M	**Injection volume:**	10.000
**Analysis method:**	IOD_PURITY_TEST.M	**Acq. operator:**	SYSTEM
**Last changed:**	2022-07-18 14:10:57+01:00 (modified after loading)		

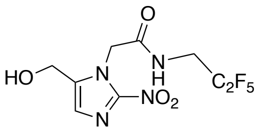

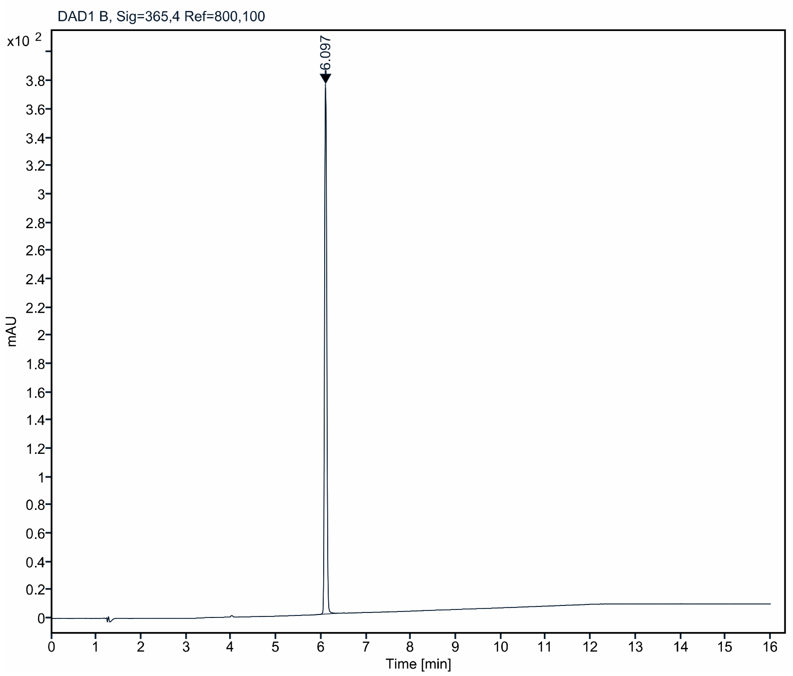

Signal: DAD1 B, Sig=365,4 Ref=800,100

RT [min]	Type	Width [min]	Area	Height	Area%	Name
6.097	BB	0.0528	1214.7408	374.9750	100.0000	
		Sum	1214.7408			

References

(1) Ahmad, P.; Mellors, A.; Steroid Biochem, J.; Fyfe, C. A.; Wolff, M. E.; Baxter, J.; Kollman, P. A.; Lee, D. L.; Kuntz, I. D.; Bloom, E.; Matulich, D.; Morris, J.; Kontula, K.; Vihko, R.; de Jager, E.; de Visser, J.; Zeelen, F.; Hansch, C.; Leo, A.; Unger, S. H.; Kim, K. H.; Nikaitani, D.; Lien, E. J.; Raynaud, J. P.; Philibert, D.; Hzadian-Boulanger, G.; Gund, P.; Shen, T. Y. *Applied Regression Analysis*; UTC, 1977; Vol. 20. https://pubs.acs.org/sharingguidelines.

(2) Lee, J. W.; Park, J.; Kim, J.; Kim, J.; Choi, C.; Min, K. H. Discovery of Potent Colony-Stimulating Factor 1 Receptor Inhibitors by Replacement of Hinge-Binder Moieties. *Eur. J. Med. Chem.* **2021**, *216,* 113298. https://doi.org/10.1016/j.ejmech.2021.113298.

(3) Molina-1996-Iminophosphorane-Mediated-Synthesis.

(4) Baird, I. R.; Patrick, B. O.; Skov, K. A.; James, B. R. Nitroimidazoles with a Halogen-Containing Side-Chain. *Can. J. Chem.* **2018**, *96*, 299–310. https://doi.org/10.1139/cjc-2017-0604.

(5) Skwarska, A.; Calder, E. D. D.; Sneddon, D.; Bolland, H.; Odyniec, M. L.; Mistry, I. N.; Martin, J.; Folkes, L. K.; Conway, S. J.; Hammond, E. M. Development and Pre-Clinical Testing of a Novel Hypoxia-Activated KDAC Inhibitor. *Cell Chem. Biol.* **2021**, *28*, 1258-1270.e13. https://doi.org/10.1016/J.CHEMBIOL.2021.04.004.
